# In pursuit of larger lipophilicity enhancement: an investigation of sugar deoxychlorination†

**DOI:** 10.1039/d5ob00037h

**Published:** 2025-02-05

**Authors:** Jonas Van De Velde, Ariadna Calderón Rodríguez, Zhong Wang, David E. Wheatley, Bruno Linclau

**Affiliations:** a Department of Organic and Macromolecular Chemistry, Ghent University Ghent 9000 Belgium Bruno.linclau@ugent.be; b School of Chemistry, University of Southampton Southampton SO17 1BJ UK

## Abstract

The excessive hydrophilicity of carbohydrates hampers their application in drug discovery. Deoxyfluorination is one of the strategies to increase sugar lipophilicity. However, lipophilicities of dideoxy-difluorinated monosaccharides are still well below the desired range for oral drug candidates. Here we investigate the power of deoxychlorination to increase sugar lipophilicities. A series of dideoxygenated chloro-fluorosugars was synthesized and for these substrates it was shown that deoxychlorination increased the log *P* by an average of 1.37 log *P* units, compared to 0.83 log *P* units for analogous deoxyfluorination. This shows the potential of deoxychlorination of carbohydrates to increase lipophilicity while limiting the number of potentially important hydrogen bond donating groups to be sacrificed, and will be of interest for glycomimetic development.

## Abstract 2

Carbohydrate deoxychlorination is shown to be a powerful tool to significantly increase sugar lipophilicities compared to analogous deoxyfluorinated monosaccharides.

## Graphical abstract

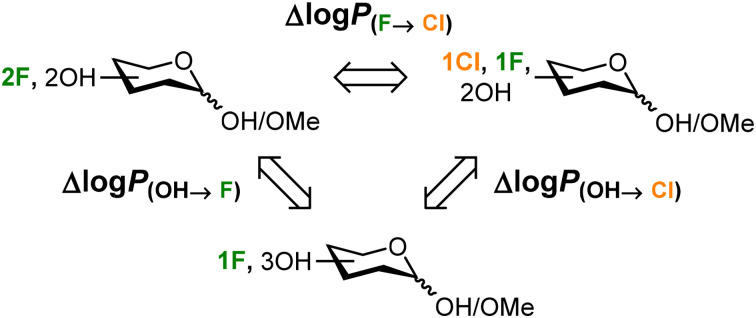

Given the pivotal role of carbohydrates in human health,^1^ there is much interest in investigating and manipulating protein–carbohydrate interactions or activities of carbohydrate-processing enzymes.^2^ The sugar scaffold itself is a very challenging starting point for drug development, with its very high hydrophilicity/very low lipophilicity (log *P*) as one of the main reasons.^3^ One of the strategies in glycomimetic design thus rests on reducing the hydrophilic character, for example by the functionalization of sugar alcohols with apolar groups, alcohol deoxygenation or deoxyfluorination.^4–7^ Our group reported a straightforward method for lipophilicity determination of the non UV-active fluorinated carbohydrates, and it was established that each successive deoxyfluorination increased the log *P* by an order of magnitude, with variations depending on fluorination position and stereochemistry.^8^ This latter aspect has been further investigated in detail by the Giguère group.^9,10^

While there are reports that chlorinated glycans bind to proteins, including examples with higher and lower affinity,^11^ it is remarkable that compared to sugar fluorination,^12^ sugar chlorination is much less investigated in glycomimetic design. This is surprising given that sucralose (Fig. 1), a trichlorinated sucrose derivative which is used as an artificial sweetener,^13,14^ is arguably the most synthesized halogenated sugar. It is resistant against enzymatic hydrolysis – hence its non-calorific properties – and generally possesses good chemical stability due to the strengthening of the C–Cl bonds by the combined effect of the many electronegative substituents.

**Fig. 1:**
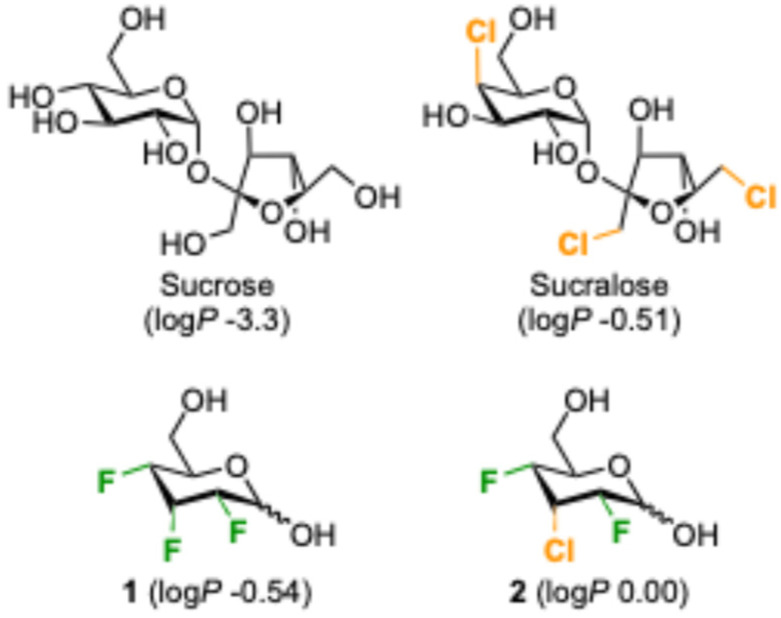
Examples of increase in lipophilicity upon deoxychlorination or fluorine-chlorine replacement of sugars.^17–19^

Chlorine introduction is also very well established in drug development, albeit mostly on aromatic rings, as a monovalent hydrophobic substituent. There is the possibility for beneficial halogen bonding effects, which in some cases contributes to marked affinity increases, and chlorination is typically associated with a lipophilicity increase on a par with methyl group introduction.^15,16^ Lipophilicity information for chlorinated sugars is scarce. The log *P* of sucralose (−0.51 log *P* units)^17^ is three orders of magnitude higher than that of sucrose (−3.3 units).^18^ Recently the Giguère group reported the higher lipophilicity of the chlorodifluoroallose analogue 2 compared to its trifluorinated analogue 1.^19^

The alcohol groups in sugars are often essential hydrogen bond donors and/or acceptors in a binding event, imposing limitations on the number of alcohol groups that can be sacrificed for increasing lipophilicity. Hence, methods to maximise the increase in lipophilicity without significant addition to the sugar conformation and steric footprint are of interest. In this context, we became interested in investigating sugar deoxychlorination and to quantify the effect of deoxychlorination on sugar lipophilicity. In this communication, we report on the effect of mono-deoxychlorination of sugars (Fig. 2).

**Fig. 2:**
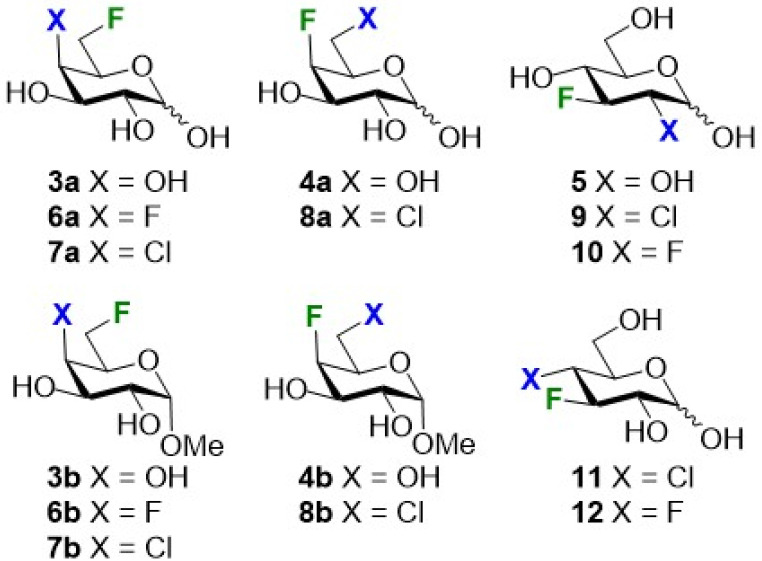
Substrates involved in this study.

The selection of the substrates was in the first instance guided both by synthetic and log *P* determination considerations. As starting points we used 6- and 4-deoxyfluorogalactose (3a and 4a), as well as 3-fluoroglucose 5, with the fluorine atom serving as handle for ^19^F NMR based log *P* determination.^8^ The corresponding methyl galactosides 3b and 4b were also investigated (Fig. 2).

The monofluorinated galactoses at C6 (3a–b) and C4 (4a–b) were obtained as described in the literature,^20–22^ and the difluorinated analogues 6a–b were synthesized starting from methyl α-d-glucopyranoside as reported by our group (not shown).^23^ The novel galactose derivatives 7a and 8a (Scheme 1) were synthesised from known 7b and 8b, both also obtained from commercially available methyl α-d-glucopyranoside,^23^ by anomeric hydrolysis in good yields.

**Scheme 1:**
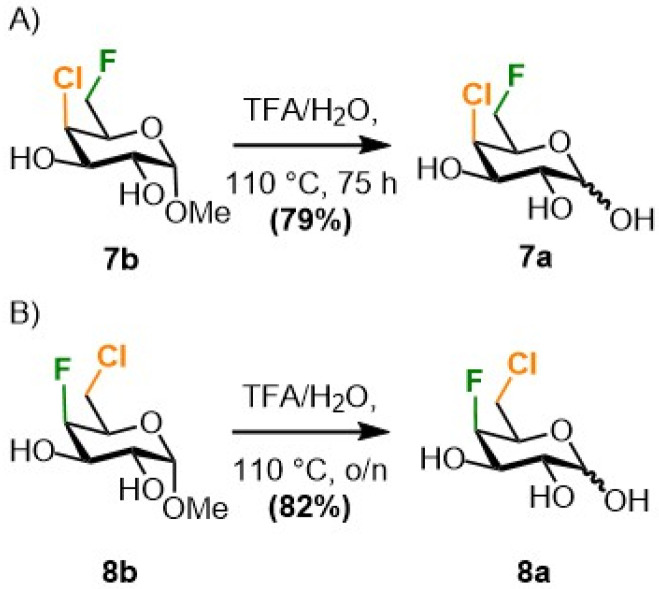
Anomeric hydrolysis of the methyl chloro-fluorogalctosides 7b^23^ and 8b^23^ towards the reducing sugars 7a and 8a.

The synthesis of 9 and 11 (Scheme 2) was achieved from levoglucosan, using a synthetic route that mirrored the known^8,10^ syntheses of the corresponding difluorinated sugars. The two required epoxide intermediates, 13 and 14, are easily available from levoglucosan on multigram scale,^24,25,26–28^ and were chosen as handles for chlorine introduction. Procter *et al.* had reported that reaction of 14 with *in situ* generated allyl magnesium chloride in THF as solvent delivered the 2-deoxy-2-chloro derivative 15 in 76% yield instead of the aniticipated allylation product.^24^ However, in our hands, reaction of a commercially available 2 M solution of allyl magnesium chloride in THF with 14 led to the allylation product. A procedure by Paulsen *et al.*, in which reaction of 13 with an ammonium fluoride and chloride mixture was reported to give 17,^25^ gave no conversion. In contrast, a method using lithium chloride, reported by Sofian and Lee on disaccharides,^29^ successfully afforded compound 15 from 14 in good yield. This reaction could easily be upscaled to a 3 g scale. The same method was then used to synthesize the 4-deoxy-4-chloro derivative 17 from 13. Treatment of the latter with base afforded the 2,3-anhydro group in 18,^25^ which allowed benzyloxy introduction at the 2-position. With 15 and 19 in hand, the stage was set for fluorine introduction at C3, which is typically effected by DAST or Deoxyfluor with retention of configuration.^30,31^ In both cases, this reaction was successful, delivering the 2,3-dideoxy-2-chloro-3-fluoro and 3,4-dideoxy-4-chloro-3-fluoro derivatives 16 and 20, both in 56% yield. A chlorine atom is a more powerful partner in neighboring group participation, potentially leading to a weaker bond between the chlorine and C2/C4, yet the epoxide opening remained fully regioselective, as dictated by the Fürst–Plattner effect^32^ (chairlike transition state). The regio- and stereoselective introduction of the C–F bond was easily established by ^1^H and ^19^F *J*-value analysis. Finally, preparation of the desired final compounds 9 and 11 could be established by hydrolytic cleavage of the 1,6-anhydro bridge with concomittent benzyl group removal in good yields.

**Scheme 2:**
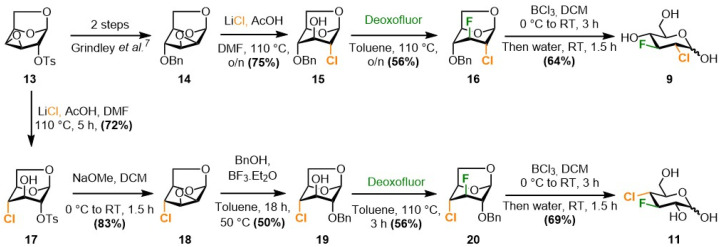
Synthesis of the two chloro-fluoro glycopyranoses 9 and 11.

The lipophilicity data of the chloro-fluorosugars is shown in Fig. 3. The chlorinated sugar derivatives invariably have a higher lipophilicity compared to the corresponding fluorinated derivatives. The log *P* values of the regioisomeric 4,6-dihalogenated galactoses 7b (log *P* −0.41) and 8b (log *P* −0.25), have an appreciable difference and are higher than the difluorinated analogue 6b (log *P* −0.90, average increase of 0.57 log *P* units). Compared to the corresponding monofluorinated saccharides 3b/4b (log *P* −1.61/−1.88) there is a significant increase of 1.20/1.63 log *P* units upon deoxychlorination at C4/C6. In contrast, analogous deoxyfluorination ‘only’ delivers a 0.71/0.98 log *P* increase. A similar picture is seen for the more polar reducing halogenated galactose equivalents 3a–8a, with similar differences between the difluorinated galactose 6a (log *P* −1.61)^33^ and the chlorofluorogalactoses 7a/8a (log *P* −1.20/−0.94), but with slightly reduced differences compared to the monofluorinated galactoses 3a and 4a (log *P* −2.16/−2.37). This is due to the lower increase in lipophilicity upon methyl glycosidation of the monofluorinated galactoses compared to the dihalogenated ones (the difference between 3a/4a with 3b/4b is ∼0.53 log *P* units, compared to ∼0.73 for the other derivatives). The reducing glucoses show larger lipophilicity differences. The log *P*-values of 9 (log *P* −0.68) and 11 (log *P* −0.72) are very similar, with a 1.5 log *P* increase compared to 3-fluoroglucose 5 (log *P* −2.19).^10^ The corresponding difluorinated glucoses 10 (log *P* −1.11)^8^ and 12 (log *P* −1.29)^10^ have a larger difference in lipophilicity but on average, the lipophilicity increase compared to 5 is ‘only’ 1.0 log *P* units.

**Fig. 3:**
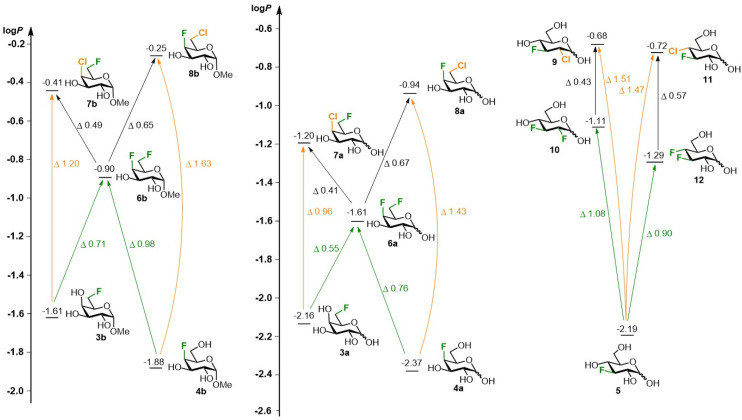
Experimentally determined log *P* values of dihalogenated glucoses and galactoses.

In summary, the synthesis of a series of dideoxygenated chloro-fluoro galactoses and glucoses has been achieved and their lipophilicities were determined. These values were compared to those measured for analogous difluorinated and monofluorinated monosaccharides. It was established that deoxychlorination leads to an increase of the log *P* with an average of 1.37 log *P* units, compared to 0.83 log *P* units for analogous deoxyfluorination. Substitution of fluorine for chlorine in carbohydrates thus results in a significant average increase in lipophilicity of 0.54 log *P* units. With these results, we show that deoxychlorination is a powerful tool to increase lipophilicity while limiting the number of potentially important hydrogen bond donating groups to be sacrificed, which will be of interest in glycomimetic design.

## Data availability

The data supporting this article have been included as part of the ESI.†

## Conflicts of interest

There are no conflicts to declare.

## Supplementary Material

OB-023-D5OB00037H-s001
