# Supplementary material for: In pursuit of larger lipophilicity enhancement: an investigation of sugar deoxychlorination
Source: Org Biomol Chem. 2025 Feb 5;23(11):2586–9. doi: 10.1039/d5ob00037h (PMC11811696; doi:10.1039/d5ob00037h)
Supplement: OB-023-D5OB00037H-s001 [file OB-023-D5OB00037H-s001.pdf]

Supplementary Information (SI) for Organic & Biomolecular Chemistry.
This journal is © The Royal Society of Chemistry 2025

SUPPORTING INFORMATION

In pursuit of larger lipophilicity enhancement: an investigation of sugar deoxychlorination

Jonas Van De Velde,[a] Ariadna Calderón Rodríguez,[a] Zhong Wang,[b] David E. Wheatley,[b] and Bruno Linclau[a,b]*

[a] Department of Organic and Macromolecular Chemistry, Ghent University, Ghent 9000, Belgium

[b] School of Chemistry, University of Southampton, Southampton SO17 1BJ (UK)

Corresponding author: Bruno.Linclau@UGent.be

Table of contents

1 Synthesis of the novel compounds 4
1.1 General Methods. 4
1.2 Synthesis of the chloro-fluoro galactopyranoses 4
1.2.1 Synthesis of 4,6-dideoxy-4-chloro-6-fluoro-D-galactose **(7a)** 4
1.2.2 Synthesis of 4,6-dideoxy-6-chloro-4-fluoro-D-galactose **(8a)** 5
1.3 Synthesis of the chloro-fluoro glucopyranoses 6
1.3.1 Synthesis of 2,3-dideoxy-2-chloro-3-fluoro-D-glucose **(9)** 6
1.3.2 Synthesis of 3,4-dideoxy-4-chloro-3-fluoro-D-glucose **(11)** 8
2 Experimental log*P* measurements 12
2.1 Determination of log*P* by ^{19}F-NMR 12
2.2 NMR experiments, NMR settings and calculation of the log*P* value 13
2.3 Raw data for all compounds 13
2.3.1 log*P* measurement of methyl 6-deoxy-6-fluoro-*α*-D-galactopyranoside **(3b)** 13
2.3.2 log*P* measurement of 6-deoxy-6-fluoro-*α*-D-galactose **(3a)** 13
2.3.3 log*P* measurement of methyl 4-deoxy-4-fluoro-*α*-D-galactopyranoside **(4b)** 14
2.3.4 log*P* measurement of 4-deoxy-4-fluoro-D-galactose **(4a)** 14
2.3.5 log*P* measurement of methyl 4,6-dideoxy-4,6-difluoro-α-D-galactopyranoside **(6a)** . 14
2.3.6 **(7b)** log*P* measurement of methyl 4,6-dideoxy-4-chloro-6-fluoro-α-D-galactopyranoside 15
2.3.7 log*P* measurement of 4,6-dideoxy-4-chloro-6-fluoro-D-galactose **(7a)** 15
2.3.8 **(8b)** log*P* measurement of methyl 4,6-dideoxy-6-chloro-4-fluoro-α-D-galactopyranoside 15
2.3.9 log*P* measurement of 4,6-dideoxy-6-chloro-4-fluoro-D-galactose **(8a)** 16

2.3.10 log*P* measurement of 2,3-dideoxy-2-chloro-3-fluoro-D-glucose **(9)** 16
2.3.11 log*P* measurement of 3,4-dideoxy-4-chloro-3-fluoro-D-glucose **(11)** 16
3 Example of NMR spectra for log*P* determination 17
3.1 Methyl 6-deoxy-6-fluoro-*α*-D-galactopyranoside (3b) 17
3.2 6-Deoxy-6-fluoro-D-galactose (3a) 19
3.3 Methyl 4-deoxy-4-fluoro-*α*-D-galactopyranoside (4b) 21
3.4 4-Deoxy-4-fluoro-D-galactose (4a) 23
3.5 4,6-Dideoxy-4,6-difluoro-α-D-galactose (6a) 25
3.6 Methyl 4,6-dideoxy-4-chloro-6-fluoro-α-D-galactospyranoside (7b) 27
3.7 4,6-Dideoxy-4-chloro-6-fluoro-D-galactose (7a) 29
3.8 Methyl 4,6-dideoxy-6-chloro-4-fluoro-α-D-galactopyranoside (8b) 31
3.9 4,6-Dideoxy-6-chloro-4-fluoro-D-galactose (8a) 33
3.10 2,3-Dideoxy-2-chloro-3-fluoro-D-glucose (9) 35
3.11 3,4-Dideoxy-4-chloro-3-fluoro-D-glucose (11) 37
4 ^{1}H NMR spectra for known compounds synthesised by a new method 39
4.1 1,6-Anhydro-4-*O*-benzyl-2-deoxy-2-chloro-*β*-D-glucopyranoside (15) (400 MHz, $CDCl_3$) 39
4.2 1,6-Anhydro-4-deoxy-4-chloro-2-*O*-tosyl-*β*-D-glucopyranoside (17) (400 MHz, $CDCl_3$) 40
4.3 1,6;2,3-Dianhydro-4-deoxy-4-chloro-*β*-D-mannopyranoside (18) (400 MHz, $CDCl_3$) 41
5 NMR spectra for novel compounds 42
5.1 NMR spectra of 4,6-dideoxy-4-chloro-6-fluoro-D-galactose (7a) 42
5.1.1 ^{1}H NMR (500 MHz, MeOH-d_4) 42
5.1.2 ^{19}F NMR (471 MHz, MeOH-d_4) 43
5.1.3 ^{19}F{^{1}H} NMR (471 MHz, MeOH-d_4) 44
5.1.4 ^{13}C{^{1}H} NMR + APT (101 MHz, MeOH-d_4) 45
5.2 NMR spectra of 4,6-dideoxy-6-chloro-4-fluoro-D-galactose (8a) 46
5.2.1 ^{1}H NMR (400 MHz, MeOH-d_4) 46
5.2.2 ^{19}F NMR (377 MHz, MeOH-d_4) 47
5.2.3 ^{19}F{^{1}H} NMR (471 MHz, MeOH-d_4) 48
5.2.4 ^{13}C{^{1}H} NMR + APT (101 MHz, MeOH-d_4) 49
5.3 NMR spectra of 1,6-anhydro-4-*O*-benzyl-2,3-dideoxy-2-chloro-3-fluoro-β-D-glucopyranoside (16) 50
5.3.1 ^{1}H NMR (400 MHz, $CDCl_3$) 50
5.3.2 ^{19}F NMR (377 MHz, $CDCl_3$) 51
5.3.3 ^{19}F{^{1}H} NMR (471 MHz, $CDCl_3$) 52
5.3.4 ^{13}C{^{1}H} NMR + APT (101 MHz, $CDCl_3$) 53
5.4 NMR spectra of 2,3-dideoxy-2-chloro-3-fluoro-D-glucose (9) 54

5.4.1 ^{1}H NMR (400 MHz, acetone-d_6) ... 54
5.4.2 ^{19}F NMR (377 MHz, acetone-d_6) ... 55
5.4.3 $^{19}F\{^{1}H\}$ NMR (471 MHz, acetone-d_6) ... 56
5.4.4 $^{13}C\{^{1}H\}$ NMR (101 MHz, acetone-d_6) ... 57
5.5 NMR spectra of 1,6-anhydro-2-*O*-benzyl-4-deoxy-4-chloro-β-D-glucopyranoside (19) ... 58
5.5.1 ^{1}H NMR (400 MHz, $CDCl_3$) ... 58
5.5.2 $^{13}C\{^{1}H\}$ NMR + APT (101 MHz, $CDCl_3$) ... 59
5.6 NMR spectra of 1,6-anhydro-2-*O*-benzyl-3,4-dideoxy-4-chloro-3-fluoro-β-D-glucopyranoside (20) ... 60
5.6.1 ^{1}H NMR (400 MHz, $CDCl_3$) ... 60
5.6.2 ^{19}F NMR (377 MHz, $CDCl_3$) ... 61
5.6.3 $^{19}F\{^{1}H\}$ NMR (471 MHz, $CDCl_3$) ... 62
5.6.4 $^{13}C\{^{1}H\}$ NMR + APT (101 MHz, $CDCl_3$) ... 63
5.7 NMR spectra of 3,4-dideoxy-4-chloro-3-fluoro-D-glucose (11) ... 64
5.7.1 ^{1}H NMR (400 MHz, acetone-d_6) ... 64
5.7.2 ^{19}F NMR (471 MHz, acetone-d_6) ... 65
5.7.3 $^{19}F\{^{1}H\}$ NMR (471 MHz, acetone-d_6) ... 66
5.7.4 $^{13}C\{^{1}H\}$ NMR + APT (101 MHz, acetone-d_6) ... 67
6 References ... 68

1 Synthesis of the novel compounds

1.1 General Methods.

All chemical reagents were obtained from commercial sources and used without further purification. Anhydrous solvents were purchased from commercial sources. When appropriate, glassware was flame-dried under vacuum and cooled under Ar prior to use. Water or air sensitive reactions were performed under inert atmosphere, using dry solvents. Reactions were monitored by TLC (Merck Kieselgel 60 F_{254}, aluminium sheet) and a described eluent system. TLC plates were visualized under UV light (254 nm) and by staining with sugar dip (0.3% (w/v) of *N*-(1- naphthyl)ethylenediamine and 10% (v/v) conc. H_2SO_4 in EtOH), followed by brief heating. Flash column chromatography was performed on silica gel (Merck silica gel 60, particle size 40–63 *μ*m). Nuclear magnetic resonance spectra were recorded using either a Bruker Ultrashield 400 MHz (101 and 376 MHz for ^{13}C and ^{19}F NMR spectra, respectively) or 500 MHz (470 MHz for ^{19}F NMR spectra) spectrometer. The chemical shifts (*δ*) are quoted in ppm relative to residual solvent peaks as appropriate. The coupling constants (*J*) were recorded in Hertz (Hz). The coupling constants have not been averaged. The signals are shown as s for singlet, d for doublet, t for triplet, q for quadruplet, quin for quintuplet and m for multiplet, or a combination of these: dd and dt means doublet of doublet and doublet of triplet *etc.*; “dm” refers to a doublet of multiplet. Atom numbering used for NMR attribution is different from the numbers used in nomenclature of compounds. NMR attributions were performed based on the following experiments: COSY, HSQC, HMBC. HRMS profiles were measured on a Bruker Daltonics MaXis time of flight (TOF) mass spectrometer. A tolerance of 5 ppm was applied between calculated and experimental values. Melting points ± 1 °C were measured on a Kofler heating bar apparatus (Heizbank, Reichert) calibrated with acetanilide (mp = 114.5 °C). Optical rotations [*α*] were measured, for reducing sugars after equilibrating for at least 16 h in the mentioned solvent, at 589 nm on a Perkin Elmer Polarimeter Model 241, and values reported are the average of 5 measurements. Fourier-transform infrared (FT-IR) spectra were recorded on a Perkin-Elmer Spectrum100 FTIR infrared spectrometer using neat samples (solid or liquid). Absorption peaks are reported in wavenumbers (cm^{-1}) and are described as br (broad), s (strong) and w (weak).

1.2 Synthesis of the chloro-fluoro galactopyranoses

1.2.1 Synthesis of 4,6-dideoxy-4-chloro-6-fluoro-D-galactose **(7a)**

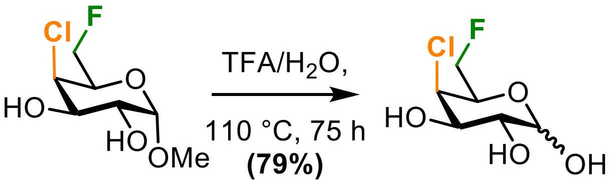

7b **7a**

A solution of crude compound **7b**[1] (145 mg, 0.675 mmol, 1 equiv.) in TFA/H_2O (1/1, 5 ml) was heated to 110 °C and stirred until full conversion (monitored via TLC, 10% MeOH in DCM) was observed

after 75 h. The mixture was allowed to cool to RT and concentrated *in vacuo*. The brown crude solid was purified by flash column chromatography (5 g, 5-15% MeOH in DCM) to afford 108 mg (0.538 mmol, 79 %) of **7a** as a mixture of the α- and β-anomers as a orange solid. The α-anomer could be isolated by precipitation in methanol as a white solid. The anomeric ratio in methanol-d_4 and D_2O proved to be 96/4 and 37/63 α/β, respectively, as determined by quantitative ^{19}F NMR and ^{1}H NMR.

R_f 0.27 (5 % MeOH in DCM); **$[\alpha]_D^{25}$** +169.6 (*at equilibrium*, c 0.5, acetone); **mp** 184-186 °C (MeOH); **IR** (neat) 3404 (w), 3343 (w), 3302 (w), 1428 (w), 1210 (w), 1156 (w), 1131 (w), 1086 (s), 1022 (s), 798 (s), 664 (w) cm^{-1}; **^{1}H NMR** (500 MHz, MeOH-d_4, *only α*) δ 5.16 (1 H, d, J = 3.7 Hz, $H_{1\alpha}$), 4.60-4.41 (3 H, m, $H_{5\alpha}$ + $H_{6\alpha}$), 4.39 (1 H, d, J = 3.5 Hz, $H_{4\alpha}$), 4.06 (1 H, dd, J = 9.9, 3.7 Hz, $H_{3\alpha}$), 3.76 (1 H, dd, J = 9.8, 3.7 Hz, $H_{2\alpha}$) ppm. **^{13}C{^{1}H} NMR + APT** (101 MHz, MeOH-d_4, *only α*) δ 94.4 (s, $C_{1\alpha}$), 84.3 (d, J = 168.2 Hz, $C_{6\alpha}$), 70.2 (s, $C_{2\alpha}$), 69.8 (s, $C_{3\alpha}$), 68.9 (d, J = 24.0 Hz, $C_{5\alpha}$), 64.5 (d, J = 5.8 Hz, $C_{4\alpha}$) ppm. **^{19}F NMR** (471 MHz, MeOH-d_4, *only α*) δ -231.4 (1 F, td, J = 46.9, 13.4 Hz, $F_{6\alpha}$) ppm. **^{19}F{^{1}H} NMR** (471 MHz, MeOH-d_4, *only α*) δ -231.4 (1 F, s, $F_{6\alpha}$) ppm. **HRMS** (ESI-) for $C_6H_9ClFO_4$ $[M - H]^-$ calcd 199.0179, found 199.0170. With isotopes: HRMS (ESI-) for $C_6H_9{}^{37}ClFO_4$ $[M - H]^-$ calcd 201.0149, found 201.0140.

1.2.2 Synthesis of 4,6-dideoxy-6-chloro-4-fluoro-D-galactose **(8a)**

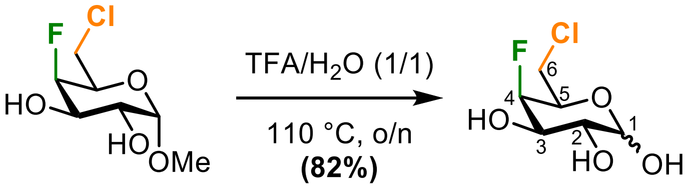

8b **8a**

A solution of compound **8b**[1] (1.00 g, 4.66 mmol, 1 equiv.) in TFA/H_2O (1/1, 20 ml) was heated to 110 °C and stirred until full conversion (monitored via TLC, 10% MeOH in DCM) was observed after overnight reaction. The mixture was allowed to cool to RT and concentrated *in vacuo*. The brown crude oil was purified by flash column chromatography (30 g, 5 - 15% MeOH in DCM) to afford 765 mg (3.81 mmol, 82%) of product **8a** as an orange solid, as a non-separable mixture of the α- and β-anomers (59/41 α/β in methanol-d_4 and 40/60 α/β in D_2O, determined by quantitative ^{19}F NMR).

R_f 0.27 (5% MeOH in DCM); **$[\alpha]_D^{25}$** +99.5 (*at equilibrium*, c 0.5, acetone); **mp** 174-176 °C (solids obtained after solvent evaporation); **IR** (neat) 3322 (br w), 2935 (w), 1449 (w), 1096 (s), 1066 (s), 1032 (s), 914 (w), 796 (s), 660 (s) cm^{-1}; **^{1}H NMR** (400 MHz, MeOH-d_4, *α/β 1/0.2 not at equilibrium*) δ 5.16 (1 H, d, J = 3.7 Hz, $H_{1\alpha}$), 4.83 (1 H, dd, J = 51.0, 2.7 Hz, $H_{4\alpha}$), 4.77 (0.2 H, dd, J = 50.8, 2.9 Hz, $H_{4\beta}$), 4.51 (0.17 H, dd, J = 7.7, 1.0 Hz, $H_{1\beta}$), 4.18 (1 H, dt, J = 28.7, 6.6 Hz, $H_{5\alpha}$), 3.88 (0.2 H, dd, J = 29.4, 2.6 Hz, $H_{3\beta}$), 3.85 (1 H, dd, J = 29.4, 2.6 Hz, $H_{3\alpha}$), 3.74 - 3.66 (2.33 H, m, $H_{2\alpha}$ + $H_{6\alpha}$ + $H_{6\beta}$), 3.64 (0.14 H, dd, J = 4.0, 1.1 Hz, $H_{5\beta}$), 3.57 (1 H, ddd, J = 11.0, 6.7, 1.2 Hz, $H_{6'\alpha}$), 3.47 (0.14 H, dd, J = 7.8, 1.6 Hz, $H_{2\beta}$) ppm. **^{13}C{^{1}H} NMR + APT** (101 MHz, MeOH-d_4) δ 98.7 (s, $C_{1\beta}$), 94.6 (s, $C_{1\alpha}$), 91.2 (d, J = 181.3 Hz, $C_{4\alpha}$), 90.4 (d, J = 181.7 Hz, $C_{4\beta}$), 75.0 (d, J = 18.2 Hz, $C_{3\beta}$), 73.6 (s, $C_{2\beta}$), 73.6 (d, J

= 18.2 Hz, $C_{5\beta}$), 70.8 (d, *J* = 17.8 Hz, $C_{5\alpha}$), 70.4 (d, *J* = 2.2 Hz, $C_{2\alpha}$), 69.9 (d, *J* = 18.2 Hz, $C_{3\alpha}$), 42.6 (d, *J* = 6.9 Hz, $C_{6\alpha}$), 42.4 (d, *J* = 6.5 Hz, $C_{6\beta}$) ppm. **^{19}F NMR** (377 MHz, MeOH-*d*$_4$, *α/β 85/15, not at equilibrium*) δ -220.1 (0.16 F, dt, *J* = 55.8, 29.6 Hz, $F_{4\beta}$), -223.2 (1 F, dt, *J* = 51.2, 29.0 Hz, $F_{4\alpha}$) ppm. **^{19}F{^{1}H} NMR** (471 MHz, MeOH-*d*$_4$, *α/β 1/0.29 not at equilibrium*) δ -220.1 (0.29 F, s, $F_{4\beta}$), -223.2 (1 F, s, $F_{4\alpha}$) ppm. **HRMS** (ESI-) for $C_6H_9ClFO_4$ $[M - H]^-$ calcd 199.0179, found 199.0175. With isotopes: HRMS (ESI-) for $C_6H_9{}^{37}ClFO_4$ $[M - H]^-$ calcd 201.0149, found 201.0145.

1.3 Synthesis of the chloro-fluoro glucopyranoses

1.3.1 Synthesis of 2,3-dideoxy-2-chloro-3-fluoro-D-glucose (9)

1.3.1.1 Synthesis of 1,6-anhydro-4-*O*-benzyl-2-deoxy-2-chloro-β-D-glucopyranoside (15)

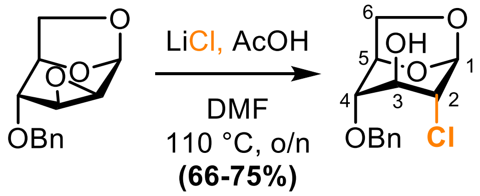

14 **15**

The following procedure was performed, using conditions as reported by Sofian *et al* for disaccharides.[2] To a solution of **14**[3, 4] (500 mg, 2.13 mmol, 1 equiv.) in DMF (14 mL) was added AcOH (0.24 mL, 4.2 mmol, 2 equiv.) and lithium chloride (2.17 g, 51.2 mmol, 24 equiv.) at RT. The mixture was heated to 110 °C and stirred overnight. After full conversion of the SM (monitored via TLC, 6/4 hexane/acetone), water (15 mL) and EtOAc (15 mL) were added. The phases were separated, and the aqueous phase was extracted with EtOAc (3 x 15 mL). The combined organic phases were washed with water (5 x 15 mL), dried over $MgSO_4$, filtered and concentrated *in vacuo*. The crude was purified by flash column chromatography (15 g, isocratic hexane/acetone 70/30) to afford 426 mg (1.59 mmol, 75 %) of product **15** as a white solid.

R_f 0.53 (hexane/acetone 65:35); **$[\alpha]_D^{25}$** -1.0 (c 1.0, $CHCl_3$); **mp** 72-74 °C (solids obtained after solvent evaporation); **IR** (neat) 3365 (w), 2895 (w), 1069 (s), 1001 (s), 809 (s), 750 (s); 696 (s) cm^{-1}; **^{1}H NMR** (400 MHz, $CDCl_3$) δ 7.41-7.30 (5 H, m, H_{Ar}), 5.56 (1 H, s, H_1), 4.76 (1 H, d, *J* = 12.2 Hz, H_{CH2OBn}), 4.68 (1 H, d, *J* = 12.3 Hz, H_{CH2OBn}), 4.64 (1 H, d, *J* = 5.2 Hz, H_5), 4.06 (1 H, dt, *J* = 6.7, 3.6 Hz, H_3), 3.97 (1 H, d, *J* = 7.5 Hz, H_6), 3.77-3.70 (2 H, m, $H_{6'}$ + H_4), 3.40 (1 H, d, *J* = 3.1 Hz, H_2), 2.45 (1 H, d, *J* = 6.0 Hz, OH) ppm. **^{13}C{^{1}H} NMR + APT** (101 MHz, $CDCl_3$) δ 137.5 (s, $C_{q,Ar}$), 128.6 (s, C_{Ar}) 128.0 (s, C_{Ar}), 127.9 (s, C_{Ar}), 102.3 (s, C_1), 78.7 (s, C_4), 75.4 (s, C_5), 72.4 (s, C_3), 71.8 (s, C_{CH2OBn}), 66.5 (s, C_6), 58.1 (s, C_2) ppm ; **HRMS** (ESI+) for $C_{13}H_{19}ClNO_4$ $[M + NH_4]^+$ calcd 288.0997, found 288.1002. With isotopes: HRMS (ESI+) for $C_{13}H_{19}{}^{37}ClNO_4$ $[M + NH_4]^+$ calcd 290.0968, found 290.0987. Data consistent with the literature.[5]

The reaction was successfully repeated on larger scale: 3.00 g of the SM, 13 g of LiCl, 1.44 ml of AcOH and 84 ml of DMF. 2.29 g (8.48 mmol, 66%) of the desired compound was obtained.

1.3.1.2 Synthesis of 1,6-anhydro-4-*O*-benzyl-2,3-dideoxy-2-chloro-3-fluoro-β-D-glucopyranoside **(16)**

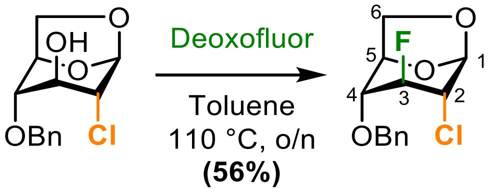

15 **16**

To a solution of **15** (500 mg, 1.85 mmol, 1 equiv.) in dry toluene (6.5 ml) at 0 °C was slowly added Deoxofluor (50 % in toluene, 1.37 ml, 3.70 mmol, 2 equiv.). The mixture was heated to 110 °C and stirred overnight. The mixture was allowed to cool down to RT, then to 0 °C and poured into H_2O (8 ml) at 0 °C. The phases were separated and the aqueous phase was extracted 3 more times with DCM (3 x 8 ml). The combined organic phases were washed with sat. aq. $NaHCO_3$ (8 ml) and brine (8 ml). The combined organic phases were dried over $MgSO_4$, filtered and concentrated *in vacuo*. The crude was purified by flash column chromatography (25 g, 80:20-60:40 hexane:EtOAc) to afford 276 mg (1.03 mmol, 56 %) of product **16** as a colourless oil.

R_f 0.58 (hexane/ethyl acetate 70:30); **$[\alpha]_D^{25}$** +12.7 (c 0.5, $CHCl_3$); **IR** (neat) 2967 (w), 2905 (w), 1113 (s), 1006 (s), 819 (s), 739 (s); 698 (s) cm^{-1}; **^{1}H NMR** (400 MHz, $CDCl_3$) δ 7.44-7.29 (5 H, m, H_{Ar}), 5.55 (1 H, s, H_1), 4.84 (1 H, apparent dquin, *J* = 44.3, 1.6 Hz, H_3), 4.78 (1 H, d, *J* = 12.3 Hz, H_{CH2OBn}), 4.68 (1 H, d, *J* = 12.1 Hz, H_{CH2OBn}), 4.66 (1 H, d, *J* = 4.4 Hz, H_5), 3.97 (1 H, dt, *J* = 7.6, 1.1 Hz, H_6), 3.90 (1 H, br dd, *J* = 17.7, 1.3 Hz, H_2), 3.79 (1 H, ddd, *J* = 7.7, 5.8, 2.0 Hz, $H_{6'}$), 3.50 (1 H, br dd, *J* = 15.7, 1.6 Hz, H_4) ppm. **^{13}C{^{1}H} NMR + APT** (101 MHz, $CDCl_3$) δ 137.0 (s, $C_{q, Ar}$), 128.6 (s, C_{Ar}), 128.2 (s, C_{Ar}), 127.9 (s, C_{Ar}), 101.0 (s, C_1), 89.7 (d, *J* = 183.1 Hz, C_3), 74.4 (d, *J* = 26.2 Hz, C_4), 74.1 (s, C_5), 71.8 (s, C_{CH2OBn}), 65.4 (d, *J* = 3.6 Hz, C_6), 53.1 (d, *J* = 25.8 Hz, C_2) ppm. **^{19}F NMR** (377 MHz, $CDCl_3$) δ -170.8 (1 F, dt, *J* = 44.4, 17.1 Hz, F_3) ppm. **^{19}F{^{1}H} NMR** (471 MHz, $CDCl_3$) δ -170.8 (1 F, s, F_3) ppm; **HRMS** (ESI+) for $C_{13}H_{18}ClNFO_3$ $[M + NH_4]^+$ calcd 290.0954, found 290.0958. With isotopes: HRMS (ESI+) for $C_{13}H_{18}{}^{37}ClNO_3$ $[M + NH_4]^+$ calcd 292.0924, found 292.0936.

1.3.1.3 Synthesis of 2,3-dideoxy-2-chloro-3-fluoro-D-glucose **(9)**

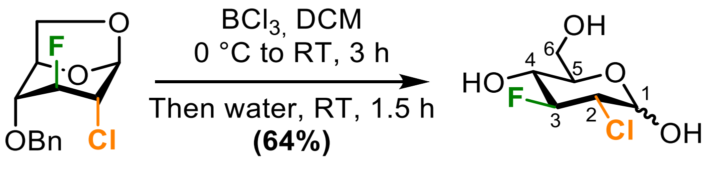

16 **9**

To a solution of **16** (276 mg, 1.03 mmol, 1 equiv.) in DCM (10 ml), was added BCl_3 (1 M in DCM, 6.18 ml, 6.18 mmol, 6 equiv.) dropwise at 0°C. The mixture was allowed to reach room temperature and stirred for 3 h. H_2O (10 ml) was added and the mixture was allowed to stir for an additional 30 minutes. The DCM was evaporated under reduced pressure and the aqueous phase was stirred for 1 h. The mixture was concentrated *in vacuo*. The brown oil crude was purified via flash column

chromatography (8 g, 5-10% MeOH in DCM) to afford 133 mg (0.664 mmol, 64%) of product **9**, as an orange wax, as a non-separable mixture of the α- and β-anomers (55/45 α/β in acetone-d_6 and 45/55 α/β in D_2O, determined by quantitative ^{1}H NMR and ^{19}F NMR).

R_f 0.21 (10% MeOH in DCM); **$[\alpha]_D^{25}$** +101.5 (*at equilibrium*, c 0.3, acetone); **IR** (neat) 3304 (br w), 2937 (w), 1697 (w), 1646 (w), 1371 (w), 1017 (s), 821 (s), cm^{-1}; **^{1}H NMR** (400 MHz, acetone-*d_6, α/β 1/0.84 not at equilibrium*) δ 6.30 (0.67 H, d, *J* = 6.7 Hz, $OH_{1β}$), 6.13 (1 H, dd, *J* = 4.6, 0.9 Hz, $OH_{1α}$), 5.29 (1 H, apparent q, *J* = 4.0 Hz, $H_{1α}$), 4.97 (0.73 H, d, *J* = 5.2 Hz, $OH_{4β}$), 4.90 (1 H, d, *J* = 5.6 Hz, $OH_{4α}$), 4.79 (0.84 H, br t, *J* = 8.0 Hz, $H_{1β}$), 4.63 (1 H, ddd, *J* = 53.0, 10.1, 8.4 Hz, $H_{3α}$), 4.43 (0.85 H, ddd, *J* = 51.2, 9.8, 8.6 Hz, $H_{3β}$), 4.00-3.86 (2 H, m, $H_{2α}$ and $H_{5α}$), 3.85-3.68 (6 H, m, $H_{6α}$ and $H_{6β}$ and $H_{4α}$ and $H_{4β}$), 3.66 (0.86 H, dt, *J* = 9.6, 1.5 Hz, $H_{2β}$), 3.58 (1 H, t, 6.3 Hz, $OH_α$), 3.41 (0.84 H, ddd, *J* = 9.7, 4.9, 1.2 Hz, $H_{5β}$), 3.34 (0.59 H, d, *J* = 12.7 Hz, $OH_β$) ppm. **^{13}C{^{1}H} NMR** (101 MHz, acetone-*d_6)* δ 98.1 (d, *J* = 186.0 Hz, $C_{3β}$), 97.0 (d, *J* = 9.1 Hz, $C_{1β}$), 95.7 (d, *J* = 184.2 Hz, $C_{3α}$), 93.6 (d, *J* = 8.4 Hz, $C_{1α}$), 76.3 (d, *J* = 8.0 Hz, $C_{5β}$) 72.7 (d, *J* = 6.9 Hz, $C_{5α}$), 70.7 (d, *J* = 18.2 Hz, $C_{4α}$), 70.3 (d, *J* = 18.2 Hz, $C_{4β}$), 62.3 (d, *J* = 16.7 Hz, $C_{2β}$), 62.0 (s, $C_{6α}$ and $C_{6β}$), 59.5 (d, *J* = 16.4 Hz, $C_{2α}$) ppm.; **^{19}F NMR** (377 MHz, acetone-*d_6*, *α/β 1/0.85 not at equilibrium)* δ -188.2 (0.85 F, br dt, *J* = 50.7, 12.8 Hz, $F_{3β}$), -193.6 (1 F, dddd, *J* = 52.8, 14.7, 10.0, 4.6 Hz, $F_{3α}$) ppm. **^{19}F{^{1}H} NMR** (471 MHz, acetone-*d_6*, *α/β 1/0.83 not at equilibrium*) δ -188.2 (0.83 F, s, $F_{3β}$), -193.5 (1 F, s, $F_{3α}$) ppm.; **HRMS** (ESI-) for $C_6H_9ClFO_4$ [M - H]$^-$ calcd 199.0179, found 199.0170. With isotopes: HRMS (ESI-) for $C_6H_9{}^{37}ClFO_4$ [M - H]$^-$ calcd 201.0149, found 201.0140.

1.3.2 Synthesis of 3,4-dideoxy-4-chloro-3-fluoro-D-glucose **(11)**

1.3.2.1 Synthesis of 1,6-anhydro-4-deoxy-4-chloro-2-*O*-*p*-toluenesulfonyl-β-D-glucopyranoside **(17)**

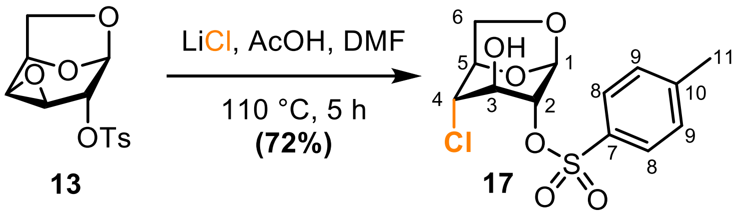

To a solution of **13**[3, 4, 6] (3.00 g, 10.1 mmol, 1 equiv.) in DMF (70 mL) was added AcOH (1.20 mL, 21.0 mmol, 2 equiv.) and lithium chloride (10.23 g, 241.3 mmol, 24 equiv.) at RT. The mixture was heated to 110°C and stirred at this temperature. Full conversion of the SM (monitored via TLC, 60/40 hexane/acetone) was observed after 5 h and, after cooling down to RT, water (70 mL) and EtOAc (70 mL) were added. The phases were separated, and the aqueous phase was extracted with EtOAc (3 x 70 mL). The combined organic phases were washed with water (5 x 70 mL), dried over $MgSO_4$, filtered and concentrated *in vacuo*. The crude was purified by flash column chromatography (100 g, isocratic hexane/acetone 70/30) to afford 2.46 g (7.34 mmol, 72%) of product **17** as a white solid.

R_f 0.31 (hexane/acetone 70:30); **$[\alpha]_D^{25}$** -63.5 (c 1.0, $CHCl_3$); **mp** 106-108 °C (solids obtained after solvent evaporation); **IR** (neat) 3512 (w), 2973 (w), 1596 (w), 1349 (s), 1169 (s), 964 (s), 812 (s), 666 (s) cm^{-1}; **^{1}H NMR** (400 MHz, $CDCl_3$) δ 7.85 (2 H, d, *J* = 8.4 Hz, H_8), 7.38 (2 H, d, *J* = 8.1 Hz, H_9), 5.43 (1 H, br t, *J* = 1.8 Hz, H_1), 4.68 (1 H, d, *J* = 5.1 Hz, H_5), 4.28 (1 H, br t, *J* = 1.2 Hz, H_2), 4.10 (2 H, m, $H_{6'}$ + H_3), 3.86 (1 H, br t, *J* = 1.4 Hz, H_4), 3.76 (1 H, dd, *J* = 7.8, 5.3 Hz, H_6), 2.79 (1 H, d, *J* = 6.3 Hz, OH), 2.47 (3 H, s, H_{11}) ppm. **^{13}C{^{1}H} NMR + APT** (101 MHz, $CDCl_3$) δ 145.5 (s, C_7), 133.0 (s, C_{10}), 130.1 (s, C_9), 128.0 (s, C_8), 100.0 (s, C_1), 76.7 (s, C_2), 76.7 (s, C_5), 72.2 (s, C_3), 67.2 (s, C_6), 57.5 (s, C_4), 21.7 (s, C_{11}) ppm.; **HRMS** (ESI+) for $C_{13}H_{19}ClNO_6S$ $[M + NH_4]^+$ calcd 352.0616, found 352.0629. With isotopes: HRMS (ESI+) for $C_{13}H_{19}{}^{37}ClNO_6S$ $[M + NH_4]^+$ calcd 354.0587, found 354.0599. Data consistent with the literature.[7]

1.3.2.2 Synthesis of 4-deoxy-4-chloro-1,6:2,3-dianhydro-β-D-mannopyranoside **(18)**

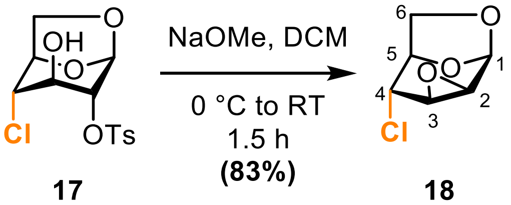

17 **18**

The following procedure was adapted from Paulsen *et al.*[7] A solution of **17** (2.00 g, 5.97 mmol, 1 equiv.) in CH_2Cl_2 (16 mL) was cooled to 0 ºC and NaOMe (25 w% in MeOH, 2.05 ml, 8.96 mmol, 1.5 equiv.) was added dropwise at 0 °C. The reaction mixture was allowed to reach RT. Full conversion of the SM (monitored via TLC, 6/4 hexane/acetone) was observed after 1.5 h and the RM was diluted with CH_2Cl_2 (8 mL) and H_2O (25 mL). The phases were separated, and the organic layer was washed with H_2O (3 x 16 mL). The combined organic layers were dried over $MgSO_4$, filtered, and concentrated *in vacuo* to give crude **18** as a yellow oil (814 mg, 5.00 mmol, 83 %). The crude was used without further purification. An analytical sample was purified by flash column chromatography (isocratic, hexane/acetone 80/20) for characterization.

R_f 0.70 (hexane/acetone 60:40); **$[\alpha]_D^{25}$** -21.4 (c 0.5, $CHCl_3$); **IR** (neat) 2970 (w), 1349 (w), 1249 (w), 1149 (w), 1118 (s) , 974 (s), 911 (s), 746 (s) cm^{-1}; **^{1}H NMR** (400 MHz, $CDCl_3$) δ 5.77 (1 H, d, *J* = 3.1 Hz, H_1), 4.58 (1 H, ddt, *J* = 4.8, 3.5, 1.3 Hz, H_5), 4.16 (1 H, br t, *J* = 0.5 Hz, H_4), 3.82-3.76 (2 H, m, H_6), 3.53 (1 H, ddd, *J* = 4.2, 3.1, 0.8 Hz, H_2), 3.35 (1 H, ddd, *J* = 3.7, 1.4, 0.6 Hz, H_3) ppm. **^{13}C{^{1}H} NMR + APT** (101 MHz, $CDCl_3$) δ 97.8 (s, C_1), 73.8 (s, C_5), 67.1 (s, C_6), 54.2 (s, C_2), 54.0 (s, C_4), 49.6 (s, C_3) ppm.; **HRMS** (ESI+) for $C_6H_8ClO_3$ $[M + H]^+$ calcd 163.0157, found 163.0152. With isotopes: HRMS (ESI+) for $C_6H_8{}^{37}ClO_3$ $[M + H]^+$ calcd 165.0127, found 165.0122. Data consistent with the literature.[7]

1.3.2.3 Synthesis of 1,6-anhydro-2-*O*-benzyl-4-deoxy-4-chloro-β-D-glucopyranoside **(19)**

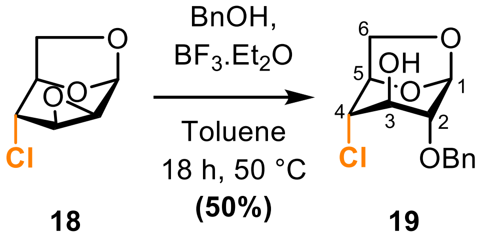

18 **19**

To a solution of **18** (711 mg, 4.37 mmol, 1 equiv.) in toluene (10 mL) was added benzyl alcohol (1.41 g, 1.36 ml, 13.1 mmol, 3 equiv.) and $BF_3 \cdot Et_2O$ (251 mg, 0.220 ml, 1.77 mmol, 0.4 equiv.) sequentially. The mixture was stirred at 50 °C for 24 h and then allowed to cool to RT. The RM was diluted with CH_2Cl_2 (20 mL) and extracted with sat. aq. $NaHCO_3$ (1 x 10 mL), and brine (1 x 10 mL). The organic phase was dried over $MgSO_4$, filtered, and concentrated *in vacuo*. The yellow oil crude was purified via flash column chromatography (66 g, 85/15-80/20 hexane/acetone) to afford 591 mg (2.18 mmol, 50 %) of product **19** as a white solid.

$\mathbf{R_f}$ 0.25 (hexane/acetone 80:20); $\mathbf{[\alpha]_D^{25}}$ -76.5 (c 0.5, $CHCl_3$); **mp** 106-108 °C (solids obtained after solvent evaporation); **IR** (neat) 3370 (w), 2978 (w), 2897 (w), 1138 (s), 1091 (s), 1064 (s), 997 (s), 923 (s), 743 (s) cm^{-1}; ^{1}**H NMR** (400 MHz, $CDCl_3$) δ 7.44-7.30 (5 H, m, H_{Ar}), 5.53 (1 H, br t, *J* = 1.5 Hz, H_1), 4.76 (1 H, d, *J* = 11.9 Hz, H_{CH2OBn}), 4.71 (1 H, d, *J* = 5.7 Hz, H_5), 4.65 (1 H, d, *J* = 12.3 Hz, H_{CH2OBn}), 4.08 - 4.01 (2 H, m, H_6 + H_3), 3.90 (1 H, br t, *J* = 1.7 Hz, H_4), 3.77 (1 H, dd, *J* = 7.8, 5.2 Hz, $H_{6'}$), 3.33 (1 H, br t, *J* = 1.6 Hz, H_2), 2.56 (1 H, br d, *J* = 6.8 Hz, OH) ppm. ^{13}**C{^{1}H} NMR + APT** (101 MHz, $CDCl_3$) δ 137.3 (s, $C_{q,Ar}$), 128.6 (s, C_{Ar}), 128.1 (s, C_{Ar}), 128.0 (s, C_{Ar}), 101.4 (s, C_1), 77.5 (s, C_2), 77.0 (s, C_5), 72.2 (s, C_{CH2OBn}), 71.9 (s, C_3), 67.2 (s, C_6), 58.7 (s, C_4) ppm.; **HRMS** (ESI+) for $C_{13}H_{15}ClNaO_4$ $[M + Na]^+$ calcd 293.0551, found 293.0552. With isotopes: HRMS (ESI+) for $C_{13}H_{15}{}^{37}ClNaO_4$ $[M + Na]^+$ calcd 295.0522, found 295.0522.

1.3.2.4 Synthesis of 1,6-anhydro-2-*O*-benzyl-3,4-dideoxy-4-chloro-3-fluoro-β-D-glucopyranoside **(20)**

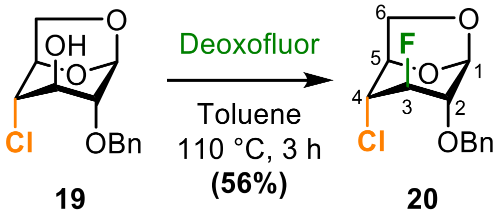

19 **20**

To a solution of **19** (200 mg, 0.738 mmol, 1 equiv.) in dry toluene (2.5 ml) at 0°C was slowly added Deoxofluor (50% in toluene, 0.550 ml, 1.48 mmol, 2 equiv.). The mixture was heated to 110 °C and stirred for 3 h (reaction monitored via TLC, hexane/EA 80/20). The mixture was allowed to cool down to RT, then to 0°C and poured into H_2O (3 ml) at 0°C. The phases were separated and the aqueous phase was extracted 3 more times with DCM (3 x 4 ml). The combined organic phases were washed with sat. aq. $NaHCO_3$ (4 ml) and brine (4 ml). The combined organic phases were dried over $MgSO_4$,

filtered and concentrated *in vacuo*. The crude was purified by flash column chromatography (5.0 g, 95:5-90:10 hexane:acetone) to afford 100 mg (1.03 mmol, 56 %) of product **20** as a colourless oil.

R_f 0.30 (hexane/ethyl acetate 80:20); **$[\alpha]_D^{25}$** -101.6 (c 0.6, $CHCl_3$); **IR** (neat) 2963 (w), 2901 (w), 1454 (w), 1334 (w), 1144 (w), 1088 (s), 1008 (s), 923 (w), 894 (w) 738 (s); 698 (s) cm^{-1}; **^1H NMR** (400 MHz, $CDCl_3$) δ 7.41-7.29 (5 H, m), 5.50 (1 H, br t, *J* = 1.5 Hz, H_1), 4.79 (1 H, apparent dquin, *J* = 44.5, 1.6 Hz, H_3), 4.75 (1 H, d, *J* = 12.1 Hz, H_{CH2OBn}), 4.69 (1 H, br dd, *J* = 5.7, 1.1 Hz, H_5), 4.64 (1 H, d, *J* = 12.2 Hz, CH_{CH2OBn}), 4.02 (1 H, dd, *J* = 17.3, 1.5 Hz, H_4), 4.02 (1 H, dt, *J* = 7.8, 0.9 Hz, H_6), 3.82 (1 H, ddd, *J* = 7.6, 5.7, 1.3 Hz, $H_{6'}$), 3.46 (1 H, dd, *J* = 15.8, 1.5 Hz, H_2) ppm. **$^{13}C\{^1H\}$ NMR + APT** (101 MHz, $CDCl_3$) δ 137.0 (s, $C_{q, Ar}$), 128.6 (s, C_{Ar}), 128.2 (s, C_{Ar}), 128.0 (s, C_{Ar}), 100.4 (s, C_1), 89.7 (d, *J* = 183.8 Hz, C_3), 75.7 (s, C_5), 73.8 (d, *J* = 24.0 Hz, C_2), 72.5 (s, C_{CH2OBn}), 66.4 (d, *J* = 2.9 Hz, C_6), 54.4 (d, *J* = 28.3 Hz, C_4) ppm. **^{19}F NMR** (377 MHz, $CDCl_3$) δ -169.6 (1 F, dt, *J* = 44.4, 16.5 Hz, F_3) ppm. **$^{19}F\{^1H\}$ NMR** (471 MHz, $CDCl_3$) δ -169.6 (1 F, s, F_3) ppm.; **HRMS** (ESI+) for $C_{13}H_{18}ClNFO_3$ [M + NH_4]$^+$ calcd 290.0954, found 290.0946. With isotopes: HRMS (ESI+) for $C_{13}H_{18}{}^{37}ClNO_3$ [M + NH_4]$^+$ calcd 292.0924, found 292.0918.

1.3.2.5 Synthesis of 3,4-dideoxy-4-chloro-3-fluoro-D-glucose **(11)**

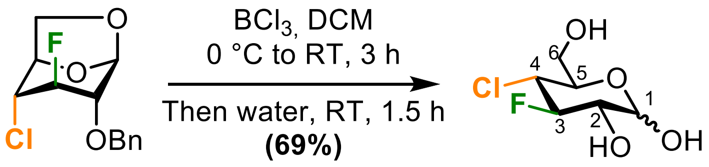

To a solution of **20** (100 mg, 0.367 mmol, 1 equiv.) in DCM (4 ml), was added BCl_3 (1 M in DCM, 2.20 ml, 2.20 mmol, 6 equiv.) dropwise at 0°C. The mixture was allowed to reach room temperature and stirred for 3 h. H_2O (4 ml) was added and the mixture was allowed to stir an additional 30 min. The DCM was evaporated under reduced pressure and the aqueous phase was stirred for 1 h. The mixture was concentrated *in vacuo*. The brown oil crude was purified via flash column chromatography (5 g, 2-10% MeOH in DCM) to afford 50 mg (0.25 mmol, 69 %) of product **11** as an orange wax, as a non-separable mixture of the α- and β-anomers (65/35 α/β in acetone-d_6 and 50.2/49.8 α/β in D_2O, determined by quantitative ^1H NMR and ^{19}F NMR).

R_f 0.29 (5% MeOH in DCM); **$[\alpha]_D^{25}$** +48.8 (*at equilibrium*, c 0.3, acetone); **IR** (neat) 3327 (br w), 2924 (w), 1452 (w), 1376 (w), 1069 (s), 1020 (s), 801 (s) cm^{-1}; **^1H NMR** (400 MHz, acetone-*d_6*, *α/β 1/0.6 not at equilibrium*) δ 6.12 (0.47 H, br d, *J* = 6.3 Hz $OH_{1\beta}$), 5.98 (1 H, br d, *J* = 4.4 Hz, $OH_{1\alpha}$), 5.24 (1 H, apparent t, *J* = 3.7 Hz, $H_{1\alpha}$), 4.83 (0.46 H, br d, *J* = 4.3 Hz, $OH_{2\beta}$), 4.65 (0.6 H, dd, *J* = 7.5, 5.7 Hz, $H_{1\beta}$), 4.64 (1 H, dt, *J* = 52.5, 9.1 Hz, $H_{3\alpha}$), 4.45 (0.78 H, ddd, *J* = 51.1, 9.4, 8.1 Hz, $H_{3\beta}$), 4.33 (1 H, d, *J* = 8.2 Hz, $OH_{2\alpha}$), 4.08-3.97 (3 H, m, H, $H_{4\alpha}$ + $H_{4\beta}$ + $H_{5\alpha}$), 3.89-3.75 (5 H, m, $H_{6\alpha}$ + $H_{6\beta}$ + $OH_{6\alpha}$ + $OH_{6\beta}$), 3.64 (1 H, apparent qd, *J* = 8.3, 4.4 Hz, $H_{2\alpha}$), 3.56 (0.66 H, ddt, *J* = 10.4, 3.8, 1.4 Hz, $H_{5\beta}$), 3.42 (0.73 H, dt, *J* = 15.3, 7.8 Hz, $H_{2\beta}$) ppm. **$^{13}C\{^1H\}$ NMR + APT** (101 MHz, acetone-*d_6*) δ 97.7 (d, *J* = 186.4 Hz, $C_{3\beta}$), 97.5 (d, *J* = 12.0 Hz, $C_{1\beta}$), 96.0 (d, *J* = 184.9 Hz, $C_{3\alpha}$), 94.0 (d, *J* = 10.5 Hz, $C_{1\alpha}$),

76.4 (d, J = 5.5 Hz, $C_{5\beta}$), 75.4 (d, J = 17.1 Hz, $C_{2\beta}$), 72.7 (d, J = 17.1 Hz, $C_{2\alpha}$), 72.7 (d, J = 4.4 Hz, $C_{5\alpha}$), 61.7 (d, J = 1.8 Hz, $C_{6\beta}$), 61.5 (d, J = 1.1 Hz, $C_{6\alpha}$), 56.8 (d, J = 17.8 Hz, $C_{4\alpha}$), 56.6 (d, J = 17.8 Hz, $C_{4\beta}$) ppm. **^{19}F NMR** (471 MHz, acetone-d_6, *α/β 1/0.6 not at equilibrium)* δ -188.5 (0.6 F, dt, J = 51.1, 12.7 Hz, $F_{3\beta}$), -193.5 (1 F, dt, J = 52.7, 10.9 Hz, $F_{3\alpha}$) ppm. **^{19}F{^{1}H} NMR** (471 MHz, acetone-d_6, *α/β 1/0.6 not at equilibrium*) δ -188.5 (0.6 F, s, $F_{3\beta}$), -193.5 (1 F, s, $F_{3\alpha}$) ppm; **HRMS** (ESI-) for $C_6H_{10}Cl_2FO_4$ [M + Cl]$^-$ calcd 234.9946, found 234.9953. With isotopes: HRMS (ESI-) for $C_6H_{10}{}^{35/37}Cl_2FO_4$ [M + Cl]$^-$ calcd 236.9916, found 236.9923 and HRMS (ESI-) for $C_6H_{10}{}^{37/37}Cl_2FO_4$ [M + Cl]$^-$ calcd 238.9886, found 238.9893.

2 Experimental log*P* measurements

2.1 Determination of log*P* by ^{19}F-NMR

Lipophilicities were determined using a previously published protocol, which was slightly adapted:[8, 9] The compound of interest (= COI) is weighed (15-25 mg) in a vial (= vial 1). The reference, with known log*P* close to the clog*P* of the compound, is weighed in a second vial (= vial 2). Beware of volatility. Seal the vial(s) between steps if necessary to minimize loss.

If the COI is more soluble in octanol, 6.5 ml of octanol is added to vial 1 and stirred between 10-15 minutes to ensure complete dissolution. Vial 1 is added over vial 2 and this is stirred for 15 min. Three oven dried pear shaped flasks, containing an oven dried stirrer bar, were sealed with a septum and allowed to cool down to 25 °C. To each flask, 2 ml of the mixture of COI and reference in octanol is transferred. Similarly, 2 ml of water is transferred to each flask. The resulting three, biphasic mixtures are stirred (at 600 rpm) for 2 h at 25 °C, and then left without stirring for at least 16 h at 25 °C to allow phase separation.

If the COI is more soluble in water, 6.5 ml of water is added to vial 1 and stirred between 10-15 minutes to ensure complete dissolution. Vial 1 is added over vial 2 and this is stirred for 15 min. Three oven dried pear shaped flasks, containing an oven dried stirrer bar, were sealed with a septum and allowed to cool down to 25 °C. To each flask, 2 ml of octanol is transferred. Similarly, 2 ml of the mixture of COI and reference in water is transferred to each flask. The resulting three, biphasic mixtures are stirred (at 600 rpm) for 2 h at 25 °C, and then left without stirring for at least 16 h at 25 °C to allow phase separation.

NMR sample preparation: For each flask, an aliquot of 0.5 mL was taken from each phase using 1 mL syringes with long needles and added to two separate NMR tubes (6 tubes in total: 3 x water and 3 x octanol). When non-interconverting species are analyzed: 0.1 ml of acetone-d_6 was added to each NMR tube and mixed by inversion (20-30 times). When dealing with interconverting species: A capillary tube, containing 0.1 ml of acetone-d_6, was added to the NMR tubes to enable signal locking. The NMR tubes were sealed with a cap and parafilm to prevent evaporation of the solvent and compounds.

As such, a statistically relevant number of measurements are performed: three per phase.

Any changes to this general protocol are mentioned in section 2.3.

2.2 NMR experiments, NMR settings and calculation of the log*P* value

For $^{19}F\{^{1}H\}$ NMR experiments, first, 1 octanol and 1 water sample were analysed with the following NMR parameters as calibration of the signal-to-noise ratio (S/N ratio): SW, 200 ppm; centered O1P between two diagnostic fluorine peaks; NS 16; D1 30 sec (octanol sample), D1 60 sec (water sample). If needed, an increased number of transients (NS) and/or narrower spectral window (SW) for a good S/N ratio (typically >200) was applied. All six NMR tubes were now analyzed with the, adapted NMR parameters. After NMR data processing, integration ratios ρ_{oct} (the three values were averaged) and ρ_{aq} (the three values were averaged) were determined. ρ_{oct} is defined as the integration ratio between the compound and the reference compound in the octanol sample, likewise for ρ_{aq}. These were used in the equation ($\log(P^{X}) = \log(P^{ref}) + \log(\rho_{oct}/\rho_{aq})$) to obtain the log*P* value of the compound.

2.3 Raw data for all compounds

2.3.1 log*P* measurement of methyl 6-deoxy-6-fluoro-*α*-D-galactopyranoside **(3b)**

Compound	Nr	Experiments[a,b] (octanol/water)	ρ_{oct}/ ρ_{wat}	log*P*	Average log*P*	Error
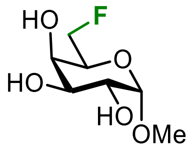	**3b**	jy2619zw3/ jy2619zw4	1.0979/ 1.1910	-1.615	-1.61	-1.613 (±0.002)
		jy2619zw5/ jy2619zw6	1.1095/ 1.1906	-1.611		

[a] Reference compound: methyl 6-deoxy-6-fluoro-α-D-glucopyranoside (log*P*: -1.58).

[b] Change from standard in NMR parameter setting: SW (140 ppm); octanol sample, NS (512).

2.3.2 log*P* measurement of 6-deoxy-6-fluoro-*α*-D-galactose **(3a)**

Compound	Nr	Experiments[a,b] (octanol/water)	ρ_{oct}/ ρ_{wat}	log*P*	Average log*P*	Error
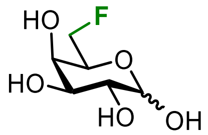	**3a**	JVlogP20903/ JVlogP20902	0.0038/ 0.2044	-2.16	-2.16	/

[a] Reference compound: 1,3-difluoro-propan-2-ol (log*P*: -0.42).

[b] Change from standard procedure: 4 mg of the compound and 7 mg of reference were weighed in the pear-shaped flask prior to adding water and octanol for stirring and equilibration. Long measurement times are needed for the octanol aliquot, due to the high hydrophilicity (low lipophilicity). Only one sample was measured to limit machine time. No capillary tubes were used to ensure solvent locking, shimming and no failed measurement.

[c] Change from standard in NMR parameter setting: octanol sample, D1 (15 s), NS (4096).

2.3.3 log*P* measurement of methyl 4-deoxy-4-fluoro-*α*-D-galactopyranoside **(4b)**

Compound	Nr	Experiments[a,b] (octanol/water)	ρ_{oct}/ ρ_{wat}	log*P*	Average log*P*	Error
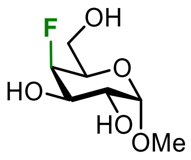	**4b**	oc2519zw7/ oc2519zw8	0.4789/ 0.9488	-1.877	-1.88	-1.880 (±0.011)
		oc2519zw9/ oc2519zw10	0.4908/ 0.9548	-1.869		
		oc2519zw11/ oc2519zw12	0.4616/ 0.9527	-1.895		

[a] Reference compound: methyl 6-deoxy-6-fluoro-α-D-glucopyranoside (log*P*: -1.58).

[b] Change from standard in NMR parameter setting: SW (140 ppm); octanol sample, NS (512).

2.3.4 log*P* measurement of 4-deoxy-4-fluoro-D-galactose **(4a)**

Compound	Nr	Experiments[a,b] (octanol/water)	ρ_{oct}/ ρ_{wat}	log*P*	Average log*P*	Error
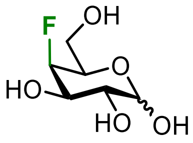	**4a**	JVlogP21003/ JVlogP21001	0.0074/ 0.6659	-2.368	-2.37	0.005
		JVlogP21004/ JVlogP21006	0.0096/ 0.8399	-2.362		

[a] Reference compound: 1,3-difluoro-propan-2-ol (log*P*: -0.42).

[b] Change from standard procedure: Experiment 1: 4 mg of the compound and 1 mg of reference were weighed in the pear-shaped flask prior to adding water and octanol for stirring and equilibration. Long measurement times are needed for the octanol aliquot, due to the high hydrophilicity (low lipophilicity). Only one sample was measured to limit machine time. No capillary tubes were used to ensure solvent locking, shimming and no failed measurement; Experiment 2: 3 mg of the compound and 1 mg of reference were weighed in the pear-shaped flask prior to adding water and octanol for stirring and equilibration. Long measurement times are needed for the octanol aliquot, due to the high hydrophilicity (low lipophilicity). Only one sample was measured to limit machine time.

[c] Change from standard in NMR parameter setting: Experiment 1: octanol sample, D1 (15 s), NS (2048); Experiment 2: octanol sample, D1 (15 s), NS (4096).

2.3.5 log*P* measurement of methyl 4,6-dideoxy-4,6-difluoro-α-D-galactopyranoside **(6a)**

Compound	Nr	Experiments[a,b] (octanol/water)	ρ_{oct}/ ρ_{wat}	log*P*	Average log*P*	Error
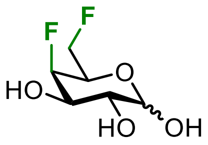	**6a**	ju1917zw2/ ju1917zw3	0.1141/ 0.8085	-1.600	-1.61	-1.610 (±0.009)
		ju1917zw8/ ju1917zw9	0.1114/ 0.8280	-1.621		
		ju1917zw10/ ju1917zw11	0.1141/ 0.8212	-1.607		

[a] Reference compound: : 2-fluoroethanol (log*P*: -0.75).

[b] Change from standard in NMR parameter setting: SW (120 ppm); octanol sample, NS (128).

2.3.6 log*P* measurement of methyl 4,6-dideoxy-4-chloro-6-fluoro-α-D-galactopyranoside **(7b)**

Compound	Nr	Experiments[a,b] (octanol/water)	ρ_{oct}/ ρ_{wat}	log*P*	Average log*P*	Error
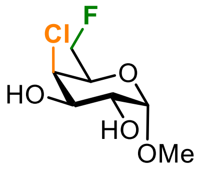	**7b**	ma0919zw2/ ma0919zw3	1.5166/ 0.6873	-0.406	-0.41	-0.408 (±0.008)
		ma0919zw4/ ma0919zw5	1.5060/ 0.6889	-0.410		
		ma0919zw6/ ma0919zw7	1.5450/ 0.7007	-0.407		

[a] Reference compound: 2-fluoroethanol (log*P*: -0.75).

[b] Change from standard in NMR parameter setting: SW (120 ppm); octanol sample, NS (256).

2.3.7 log*P* measurement of 4,6-dideoxy-4-chloro-6-fluoro-D-galactose **(7a)**

Compound	Nr	Experiments[a,b] (octanol/water)	ρ_{oct}/ ρ_{wat}	log*P*	Average log*P*	Error
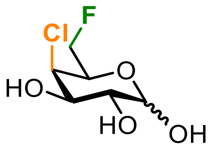	**7a**	JVlogP20703/ JVlogP20706	0.0808/ 0.7053	-1.201	-1.20	-1.197 (±0.007)
		JVlogP20704/ JVlogP20707	0.0787/ 0.6861	-1.200		
		JVlogP20705/ JVlogP20708	0.0807/ 0.6866	-1.188		

[a] Reference compound: 3-fluoro-propan-1-ol (log*P*: -0.26).

[b] Change from standard in NMR parameter setting: octanol sample, NS (256).

2.3.8 log*P* measurement of methyl 4,6-dideoxy-6-chloro-4-fluoro-α-D-galactopyranoside **(8b)**

Compound	Nr	Experiments[a,b] (octanol/water)	ρ_{oct}/ ρ_{wat}	log*P*	Average log*P*	Error
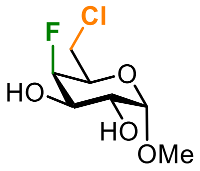	**8b**	JVlogP20803/ JVlogP20806	0.9543/ 0.6513	-0.254	-0.25	-0.253 (±0.002)
		JVlogP20804/ JVlogP20807	0.9646/ 0.6532	-0.251		
		JVlogP20805/ JVlogP20808	0.9491/ 0.6478	-0.254		

[a] Reference compound: 1,3-difluoro-propan-2-ol (log*P*: -0.42).

[b] Change from standard in NMR parameter setting: /.

2.3.9 log*P* measurement of 4,6-dideoxy-6-chloro-4-fluoro-D-galactose **(8a)**

Compound	Nr	Experiments[a,b] (octanol/water)	ρ_{oct}/ ρ_{wat}	log*P*	Average log*P*	Error
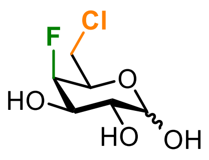	**8a**	JVlogP20603/ JVlogP20606	0.2192/ 0.7306	-0.943	-0.94	-0.943 (±0.001)
		JVlogP20604/ JVlogP20607	0.2221/ 0.7413	-0.943		
		JVlogP20605/ JVlogP20508	0.2157/ 0.7209	-0.944		

[a] Reference compound: 1,3-difluoro-propan-2-ol (log*P*: -0.42).

[b] Change from standard in NMR parameter setting: octanol sample, NS (256).

2.3.10 log*P* measurement of 2,3-dideoxy-2-chloro-3-fluoro-D-glucose **(9)**

Compound	Nr	Experiments[a,b] (octanol/water)	ρ_{oct}/ ρ_{wat}	log*P*	Average log*P*	Error
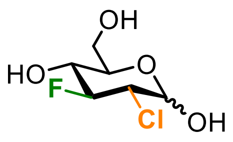	**9**	JVlogP20403/ JVlogP20406	0.1250/ 0.3322	-0.684	-0.68	-0.683 (±0.001)
		JVlogP20404/ JVlogP20407	0.1245/ 0.3288	-0.682		
		JVlogP20405/ JVlogP20408	0.1197/ 0.3174	-0.684		

[a] Reference compound: 3-fluoro-propan-1-ol (log*P*: -0.26).

[b] Change from standard in NMR parameter setting: octanol sample, NS (256).

2.3.11 log*P* measurement of 3,4-dideoxy-4-chloro-3-fluoro-D-glucose **(11)**

Compound	Nr	Experiments[a,b] (octanol/water)	ρ_{oct}/ ρ_{wat}	log*P*	Average log*P*	Error
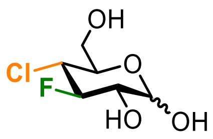	**11**	JVlogP20504/ JVlogP20505	0.1616/ 0.3219	-0.719	-0.72	-0.723 (±0.006)
		JVlogP20506/ JVlogP20507	0.1654/ 0.3306	-0.721		
		JVlogP20408/ JVlogP20409	0.1606/ 0.3282	-0.730		

[a] Reference compound: 1,3-difluoro-propan-2-ol (log*P*: -0.42).

[b] Change from standard in NMR parameter setting: octanol sample, D1 (13s), NS (1024).

3 Example of NMR spectra for log*P* determination

3.1 Methyl 6-deoxy-6-fluoro-*α*-D-galactopyranoside (3b)

<u>Octanol sample:</u>

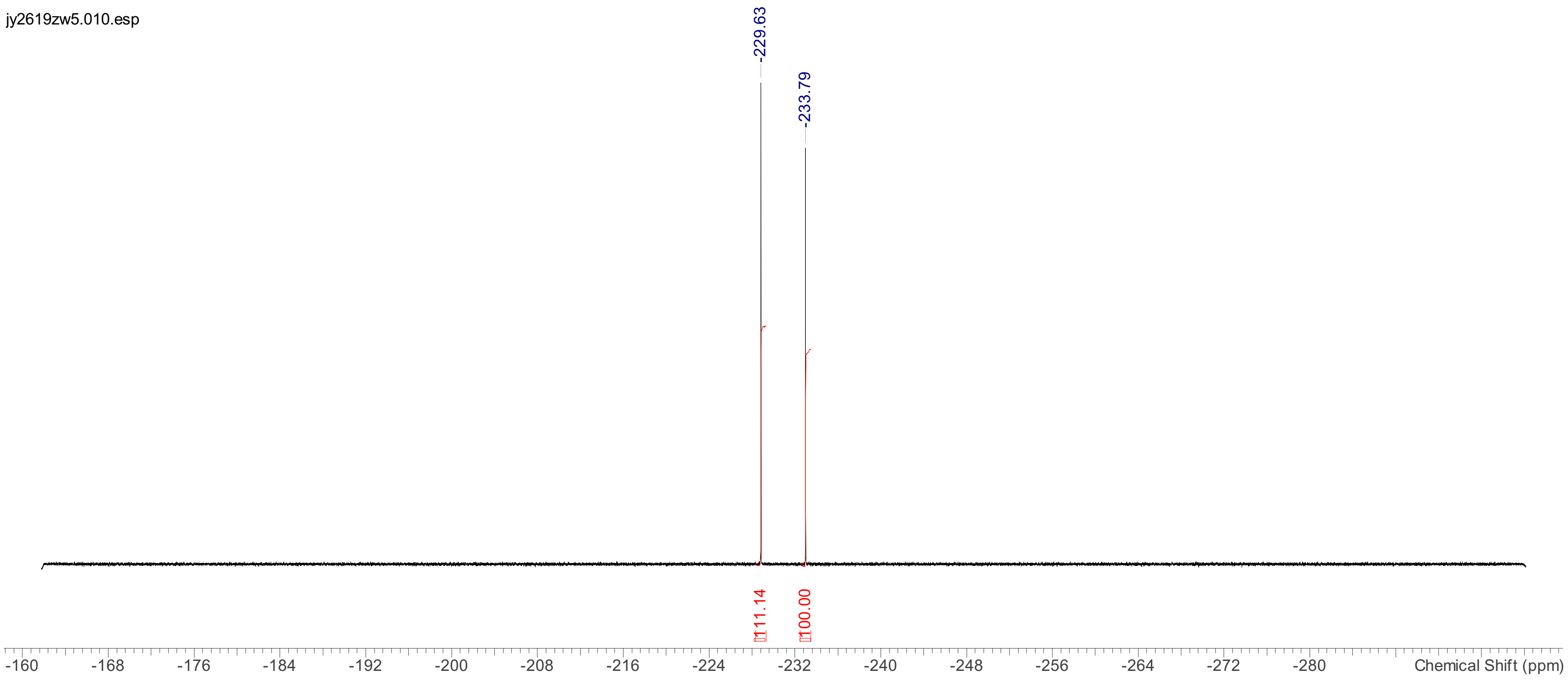

Water sample:

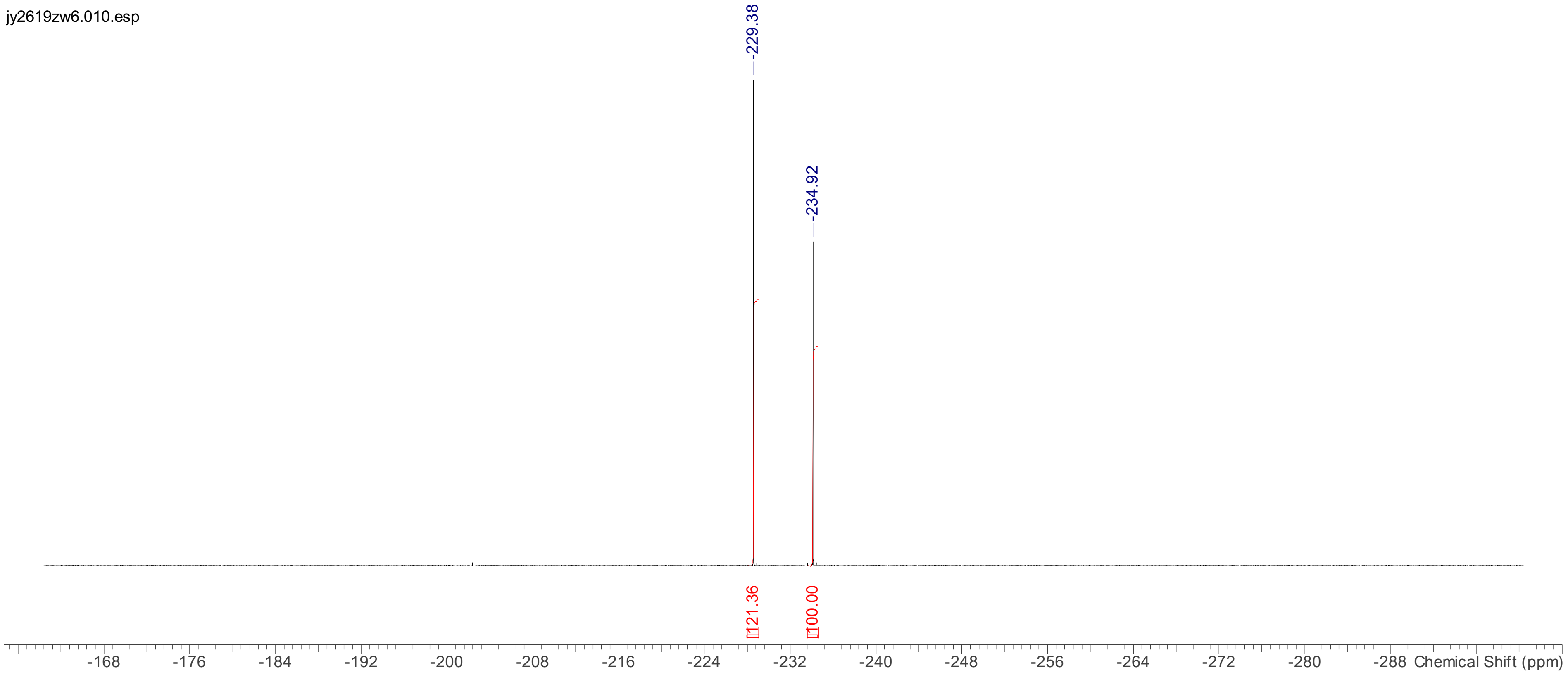

3.2 6-Deoxy-6-fluoro-D-galactose (3a)

Octanol sample:

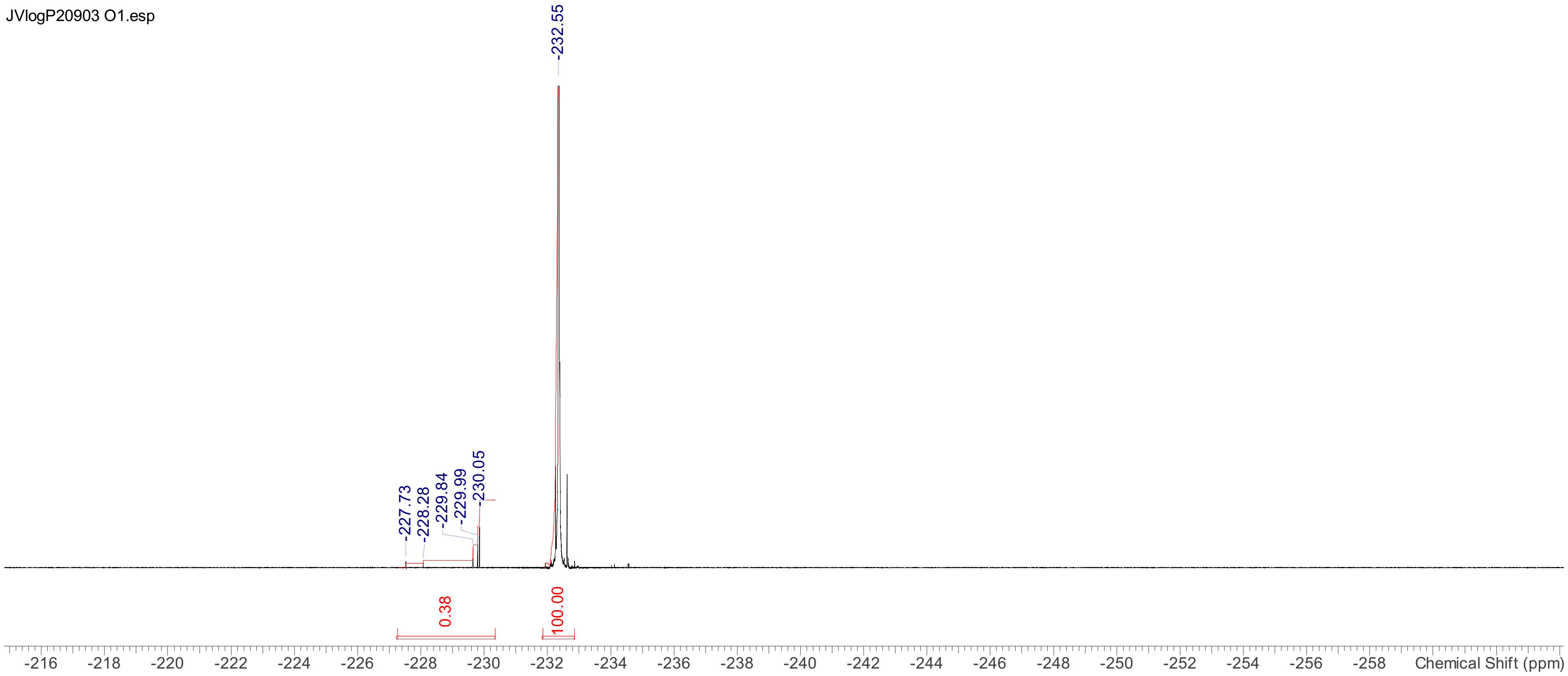

Water sample:

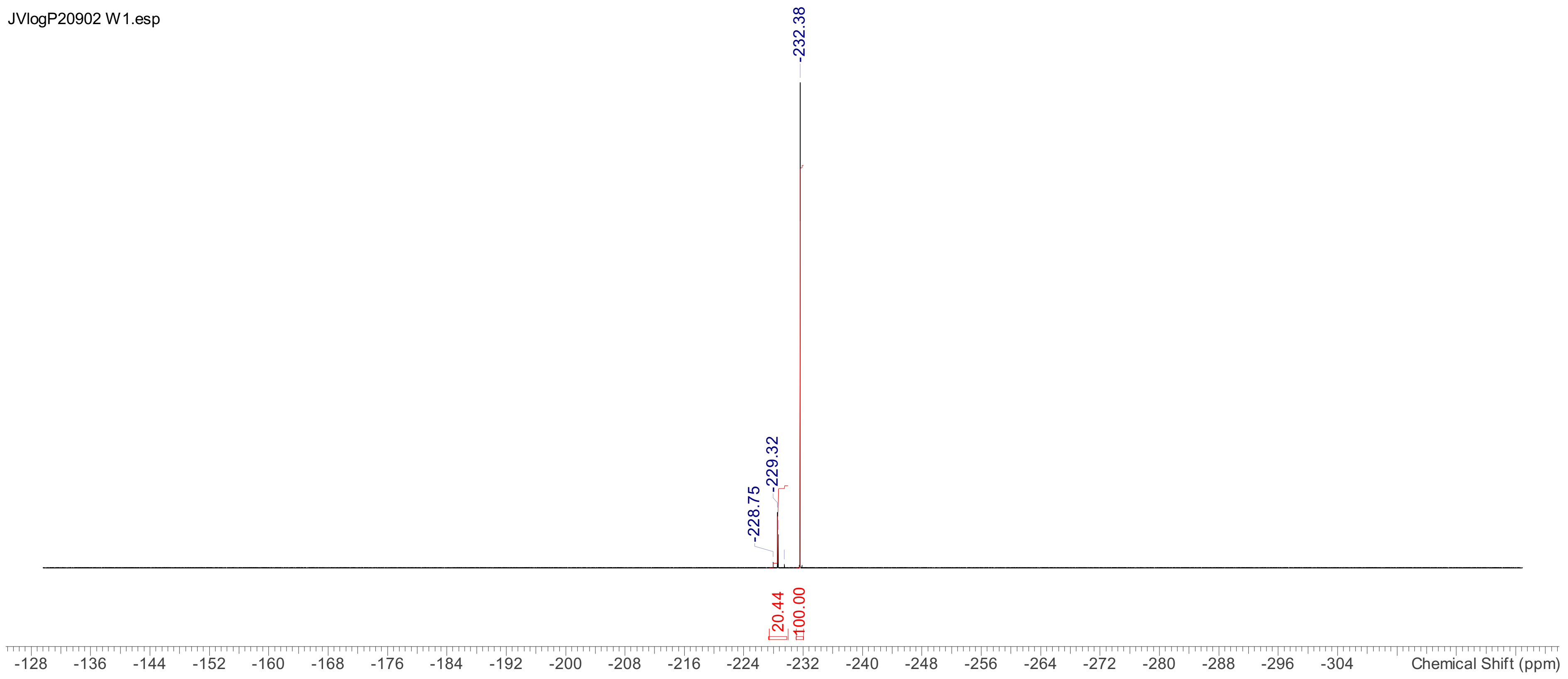

3.3 Methyl 4-deoxy-4-fluoro-*α*-D-galactopyranoside (4b)

Octanol sample:

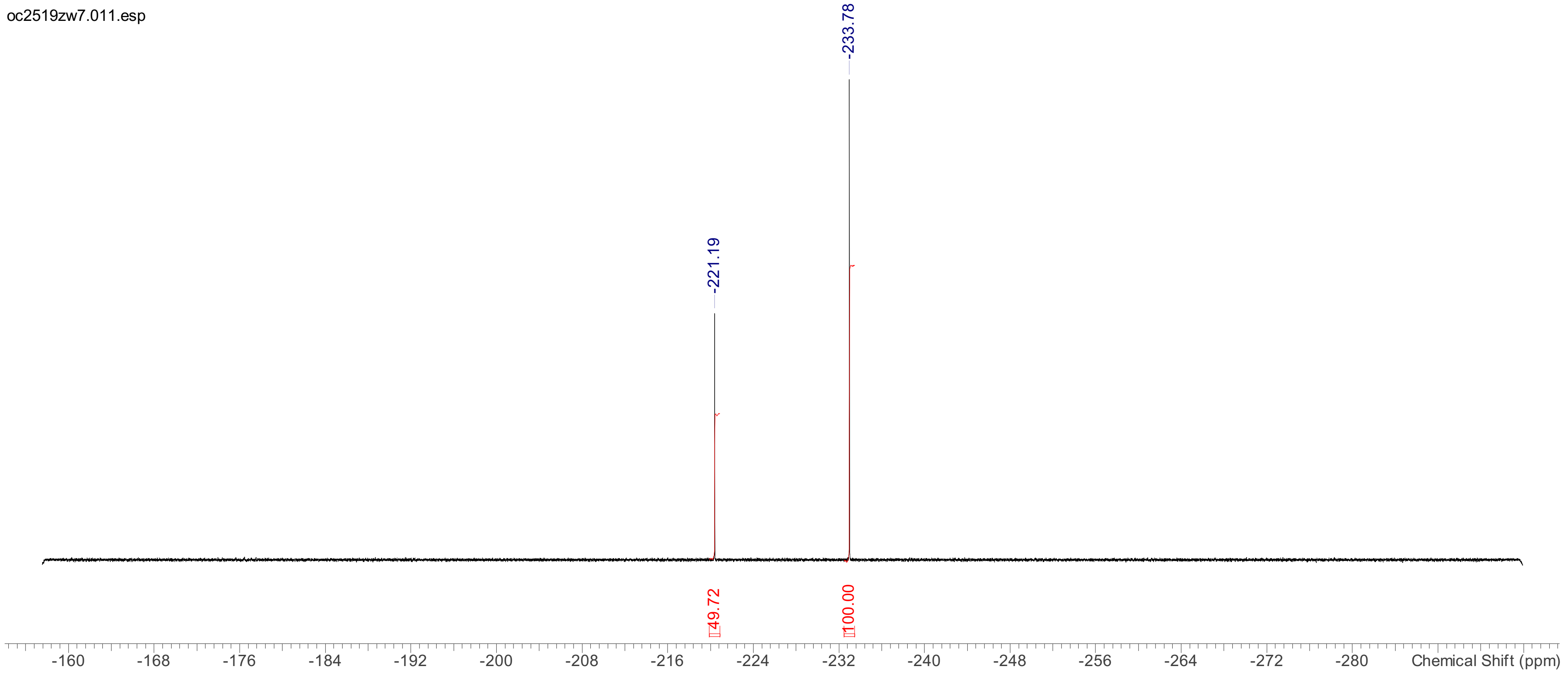

Water sample:

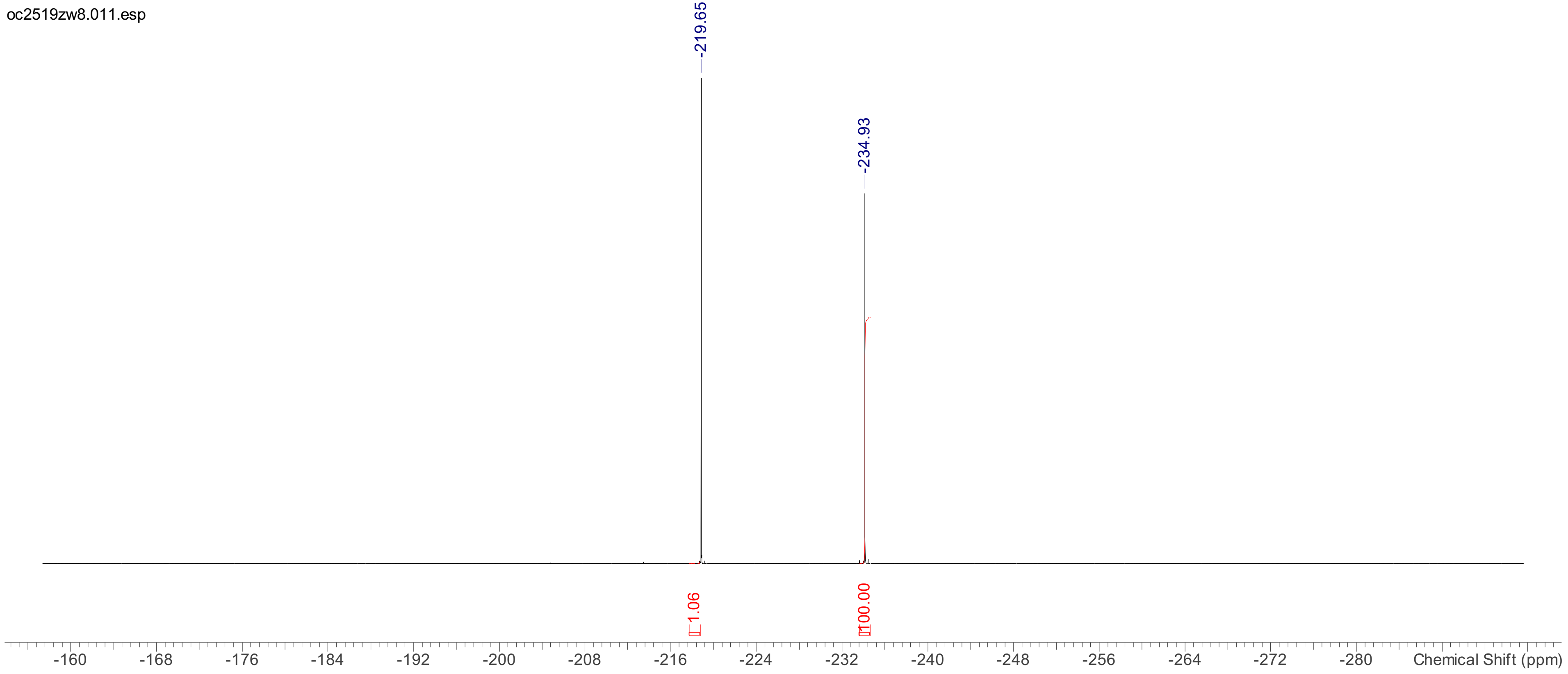

3.4 4-Deoxy-4-fluoro-D-galactose (4a)

<u>Octanol sample:</u>

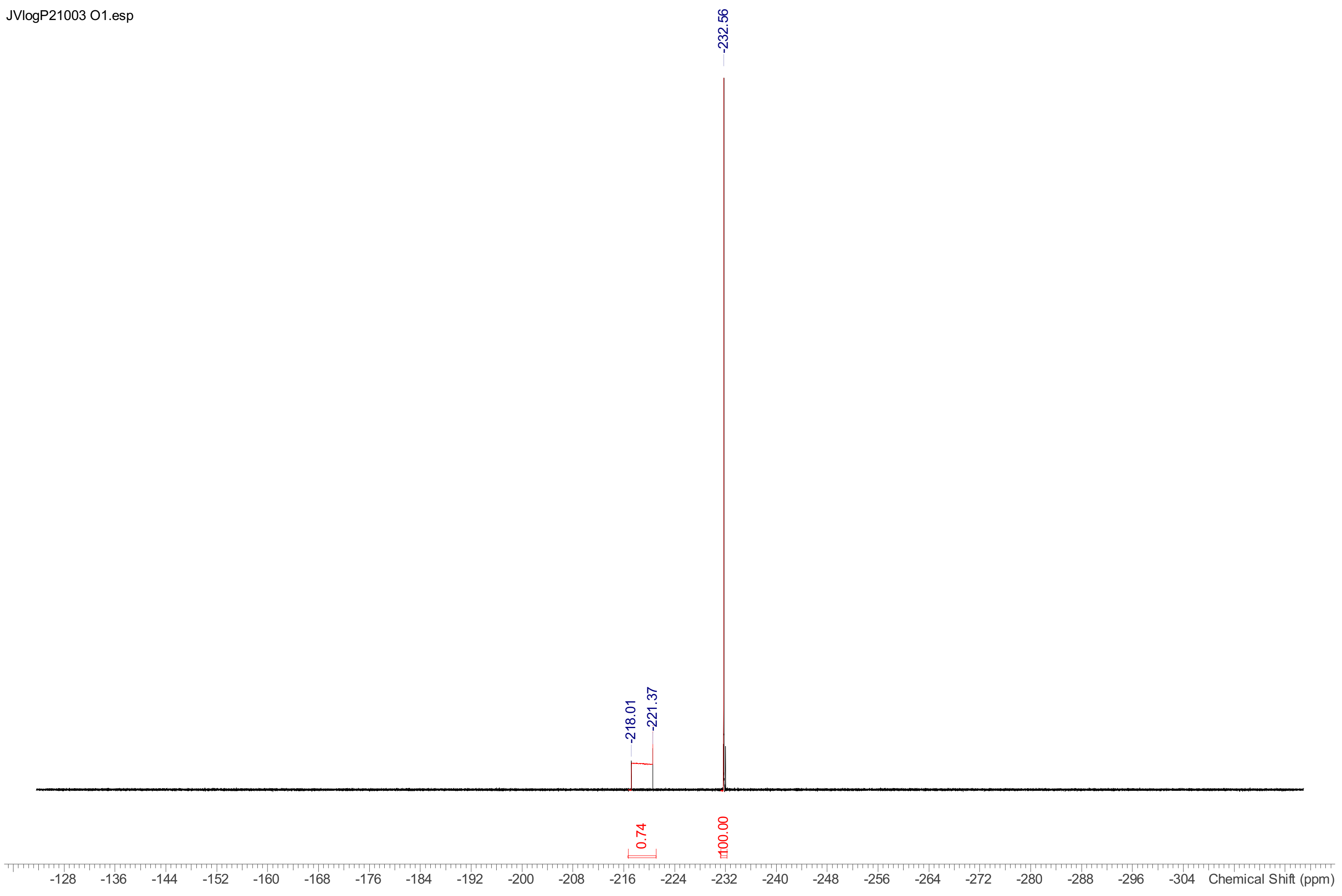

Water sample:

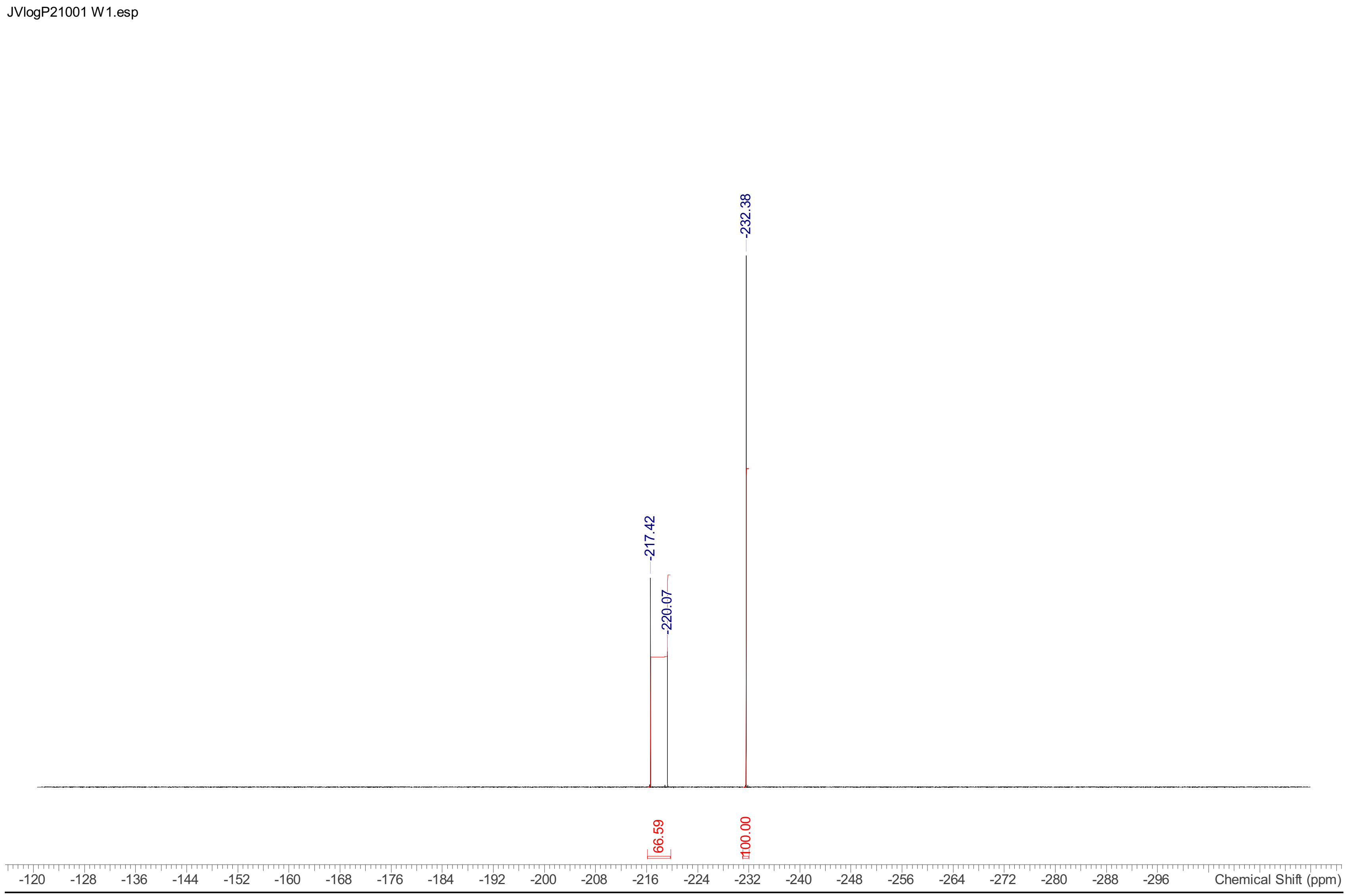

3.5 4,6-Dideoxy-4,6-difluoro-α-D-galactose (6a)

Octanol sample:

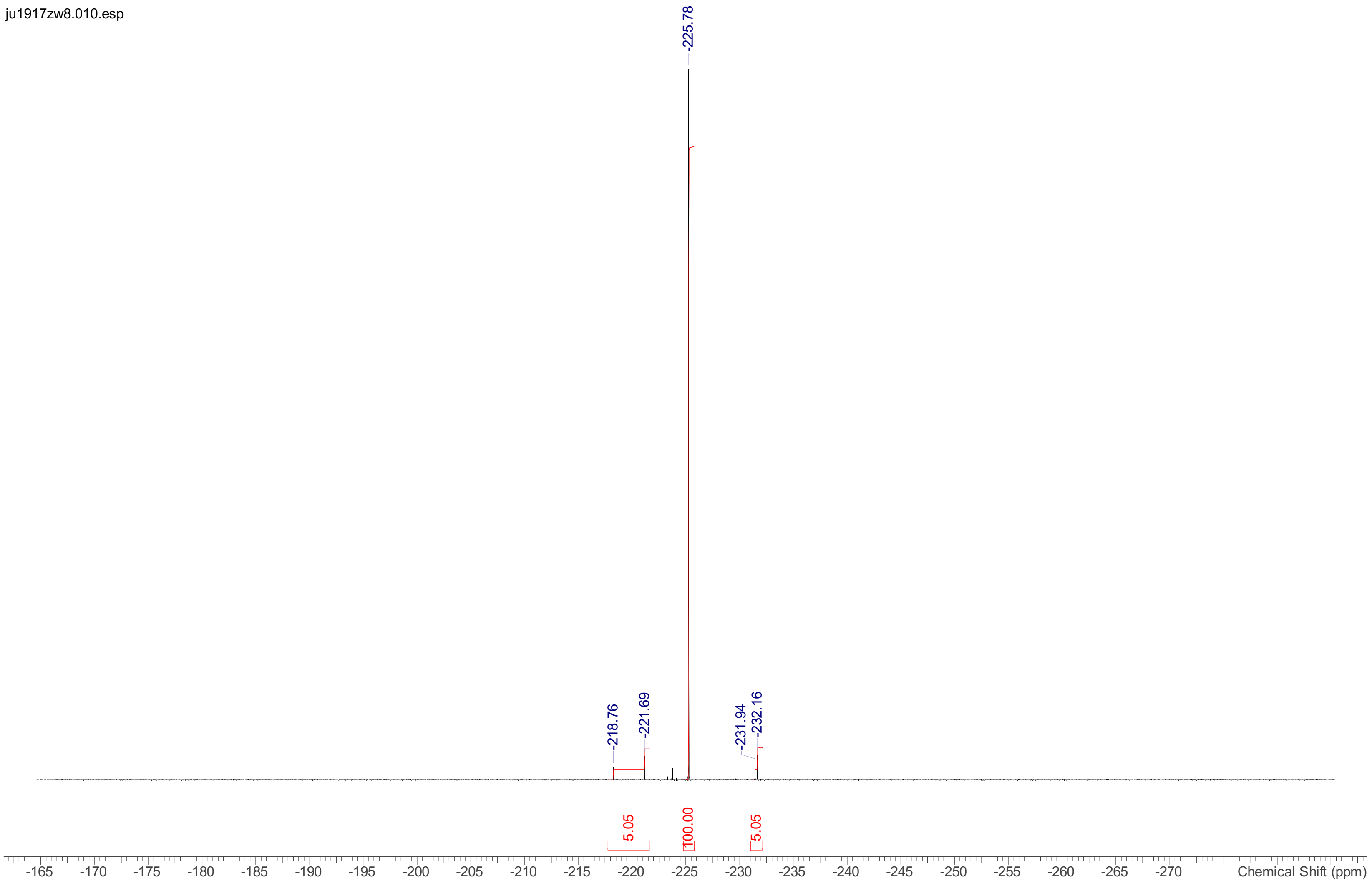

Water sample:

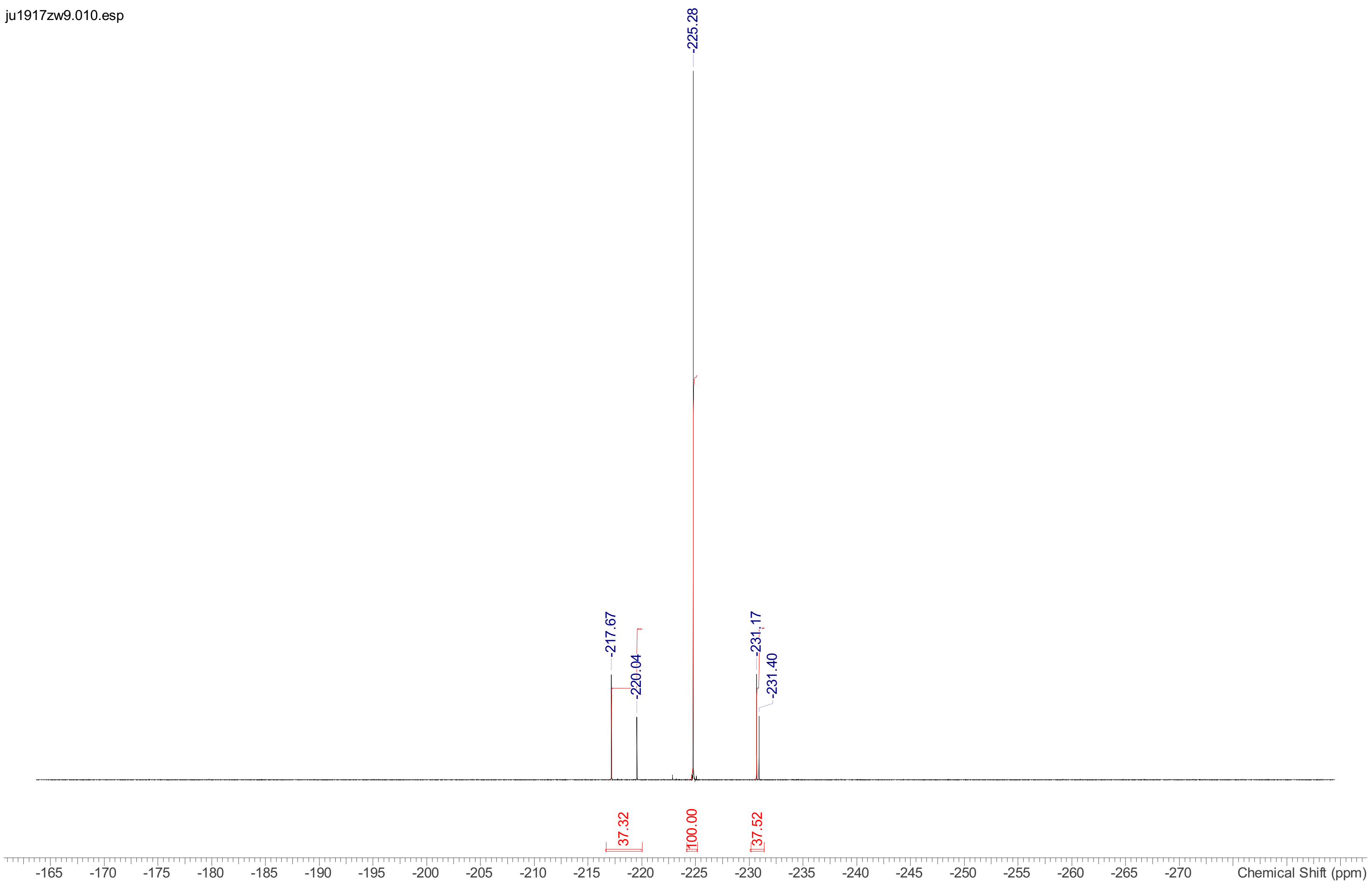

3.6 Methyl 4,6-dideoxy-4-chloro-6-fluoro-α-D-galactospyranoside (7b)

Octanol sample:

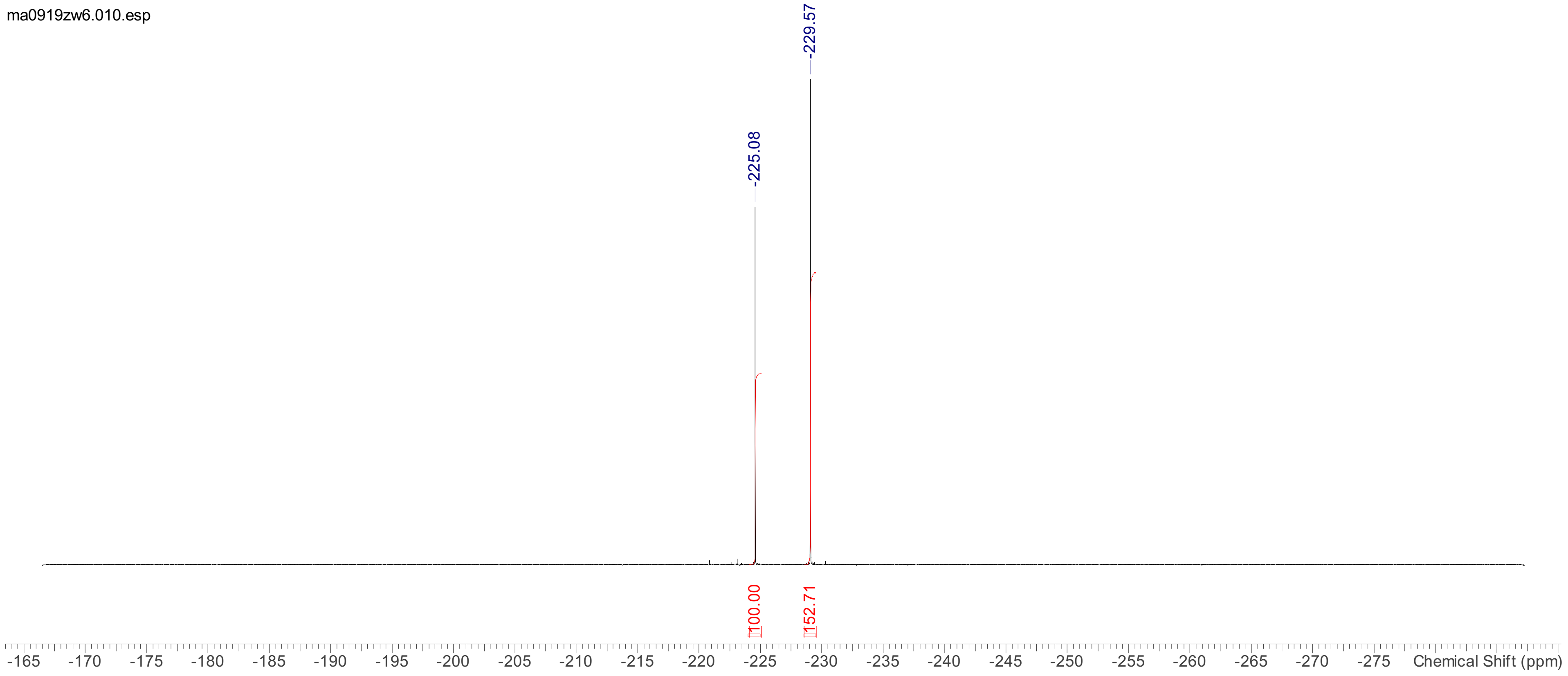

Water sample:

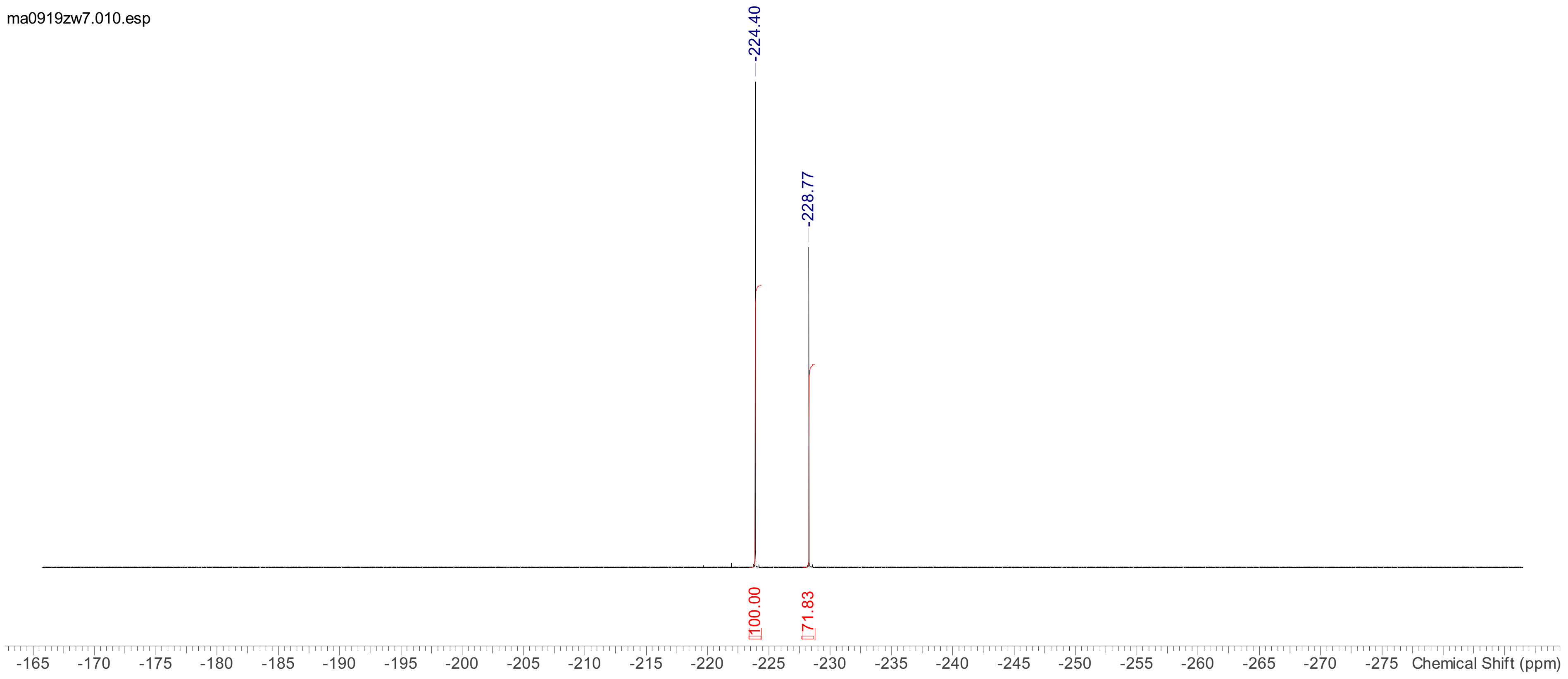

3.7 4,6-Dideoxy-4-chloro-6-fluoro-D-galactose (7a)

Octanol sample:

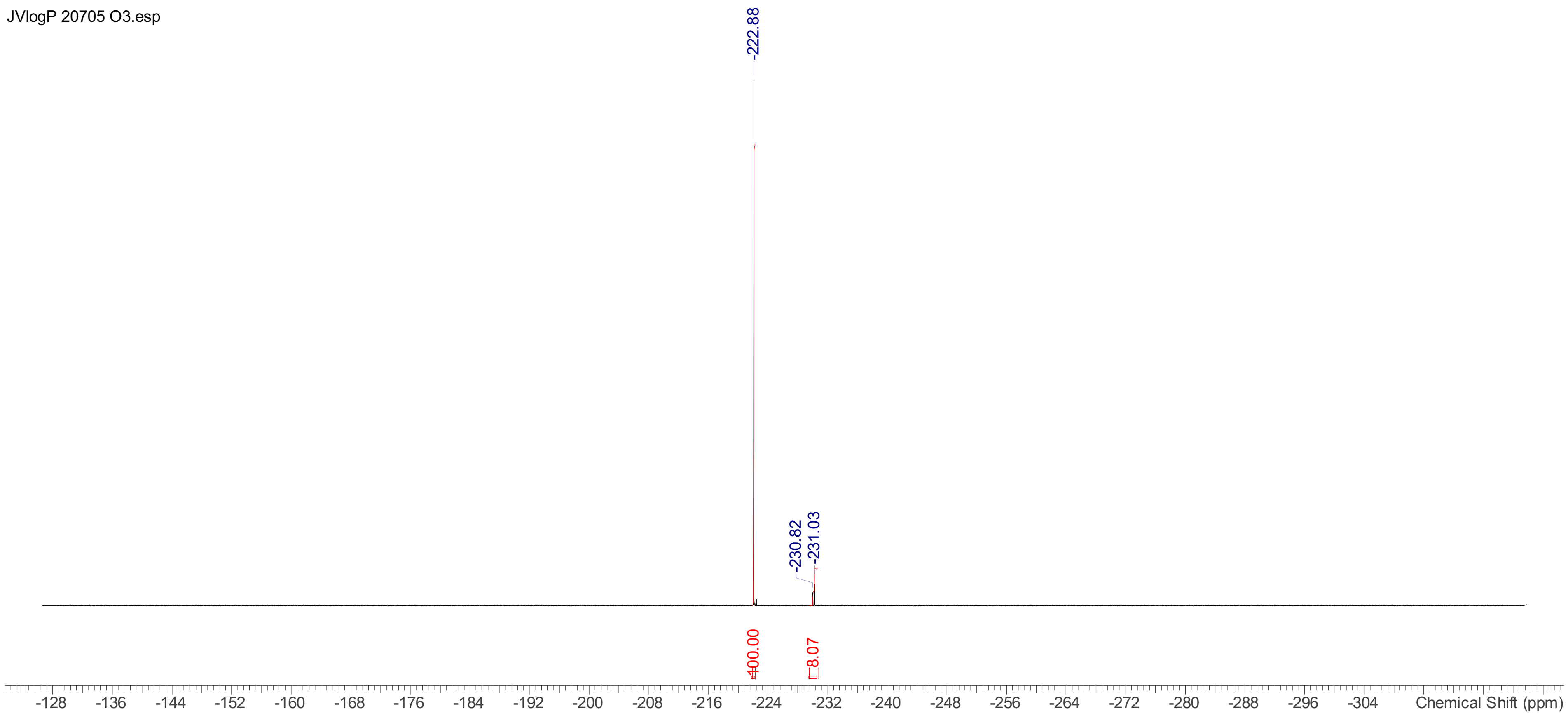

Water sample:

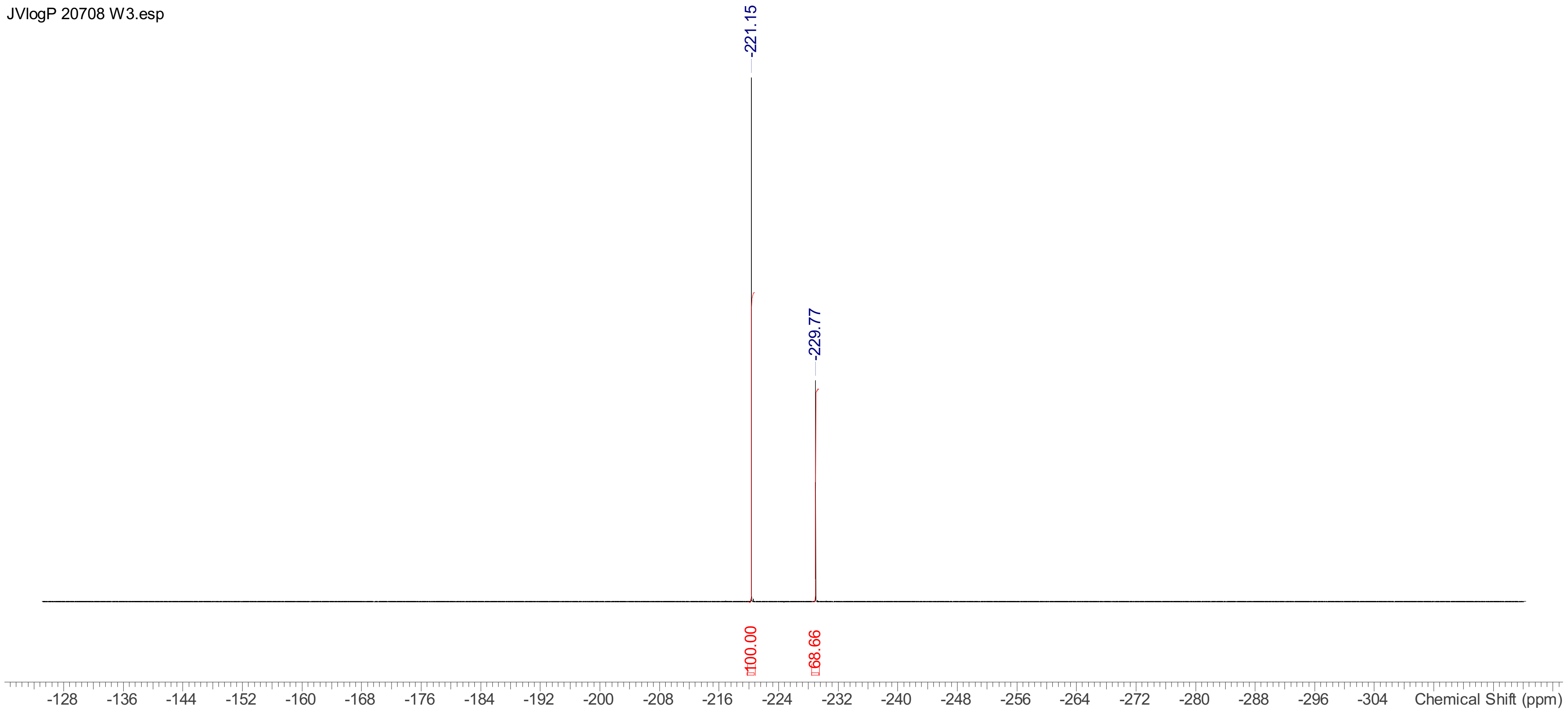

3.8 Methyl 4,6-dideoxy-6-chloro-4-fluoro-α-D-galactopyranoside (8b)

Octanol sample:

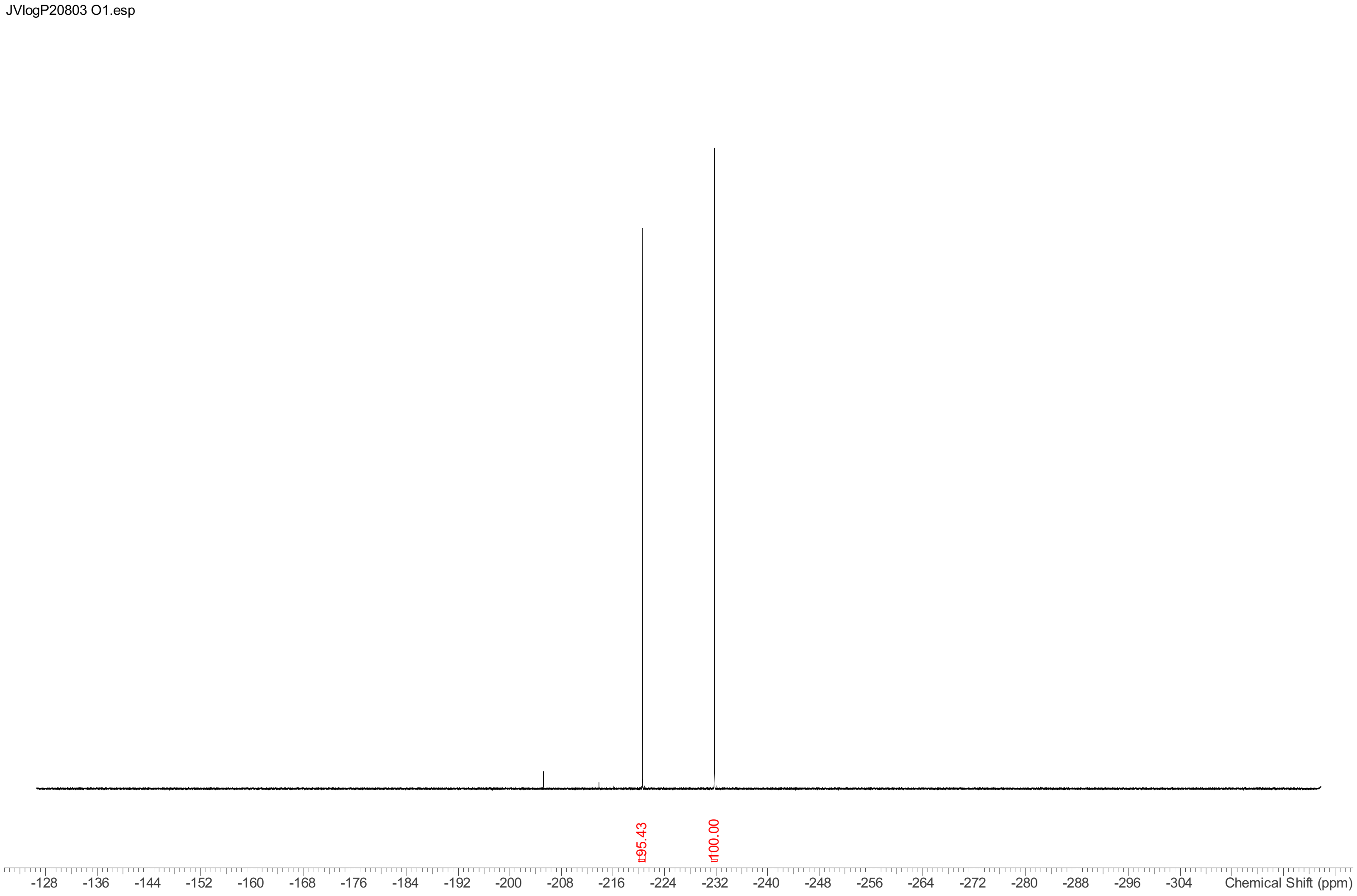

Water sample:

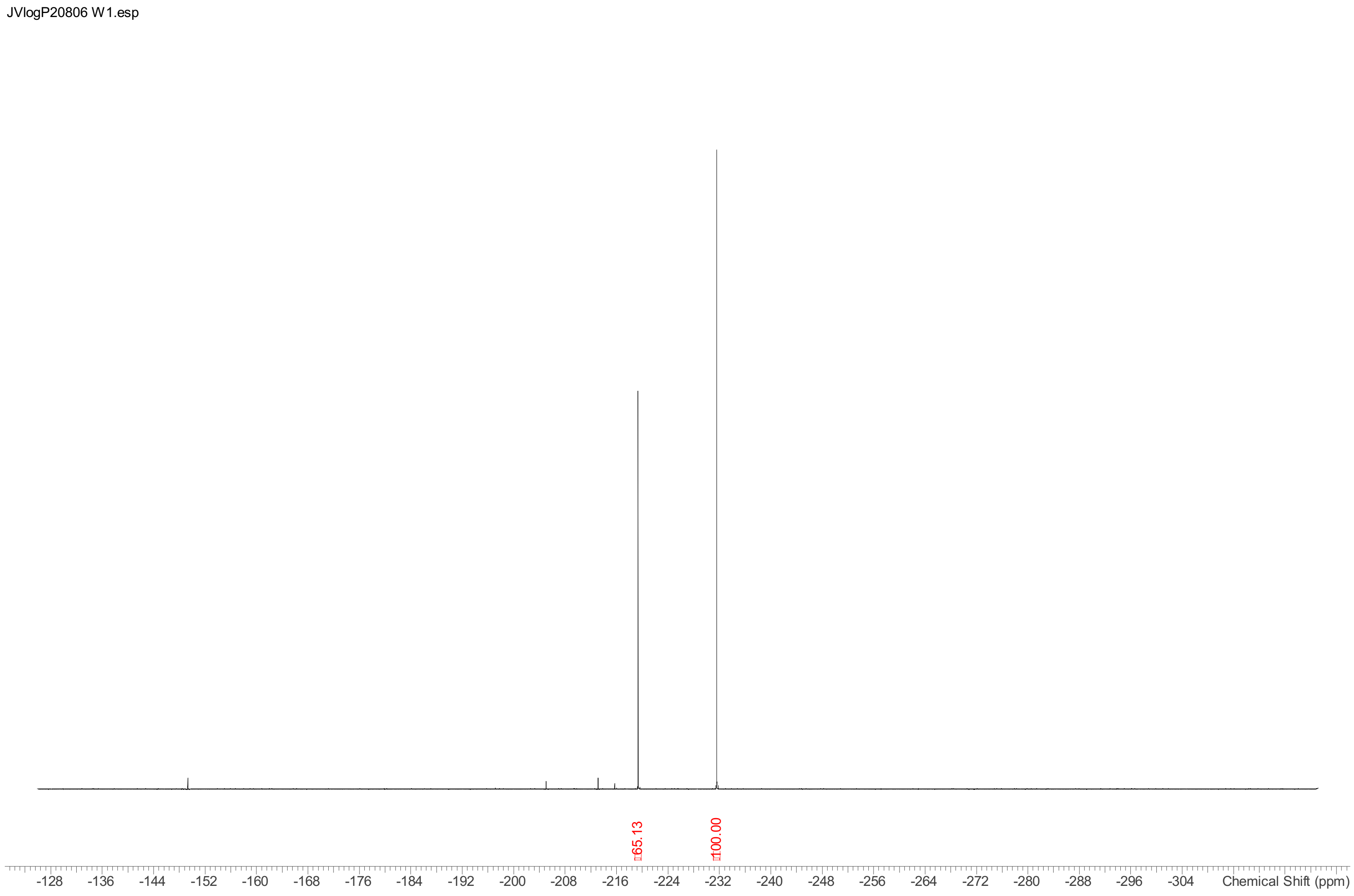

3.9 4,6-Dideoxy-6-chloro-4-fluoro-D-galactose (8a)

Octanol sample:

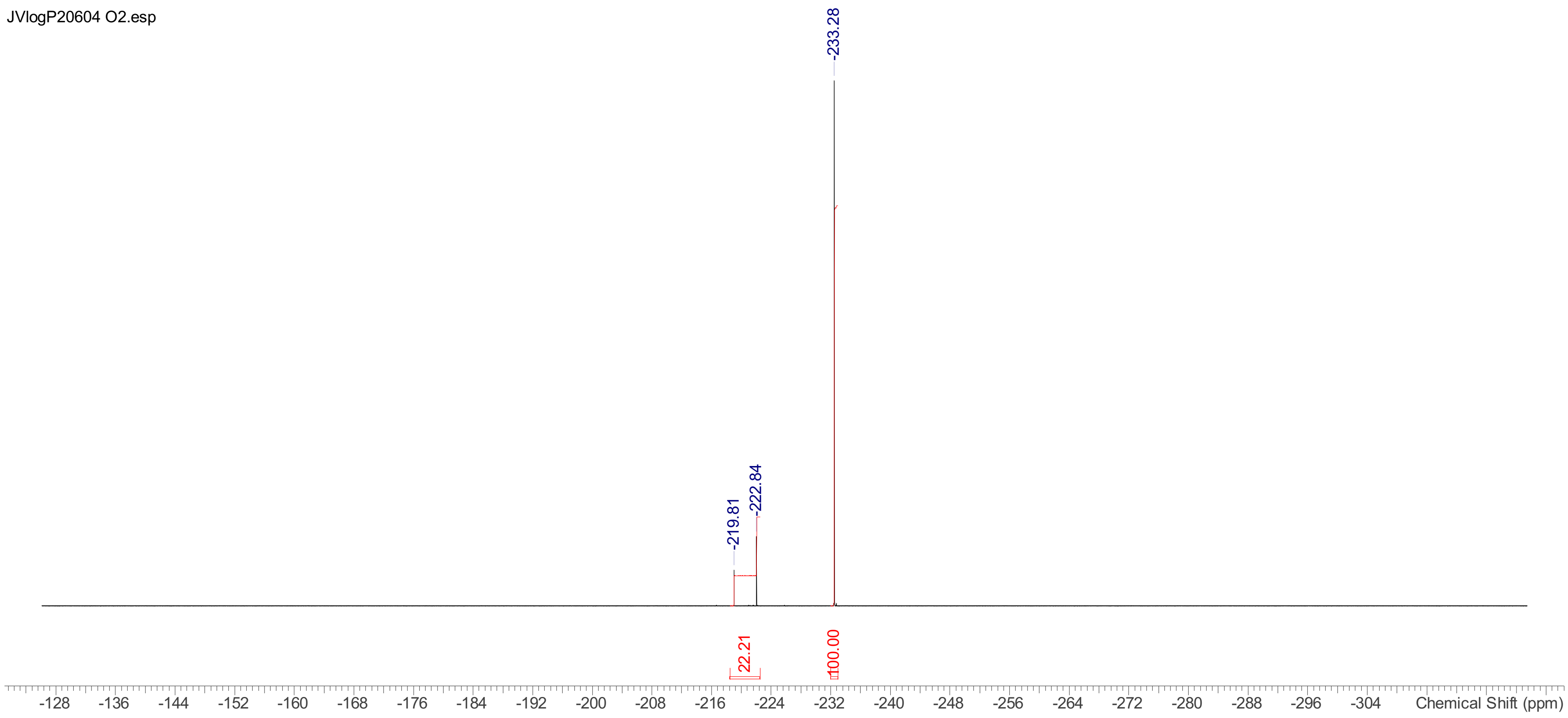

Water sample:

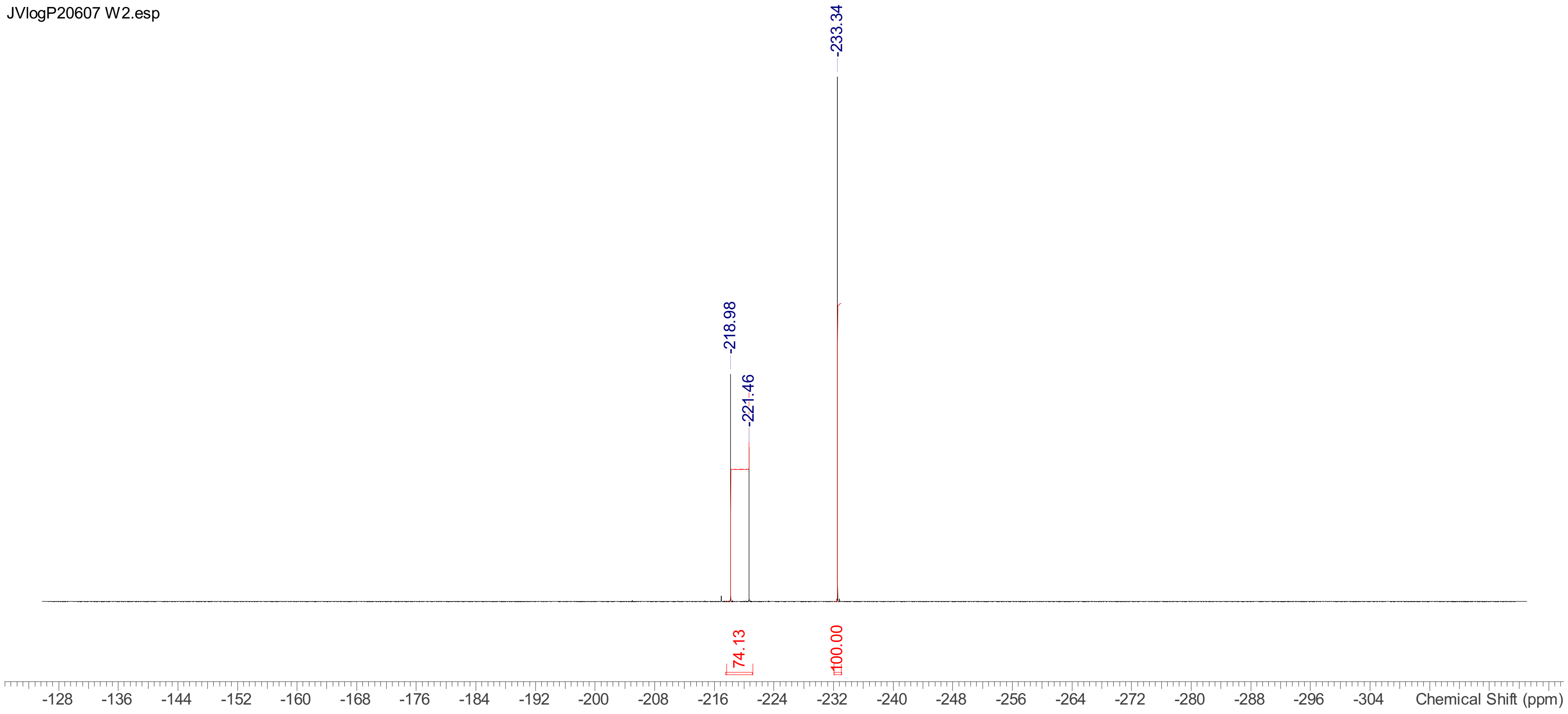

3.10 2,3-Dideoxy-2-chloro-3-fluoro-D-glucose (9)

Octanol sample:

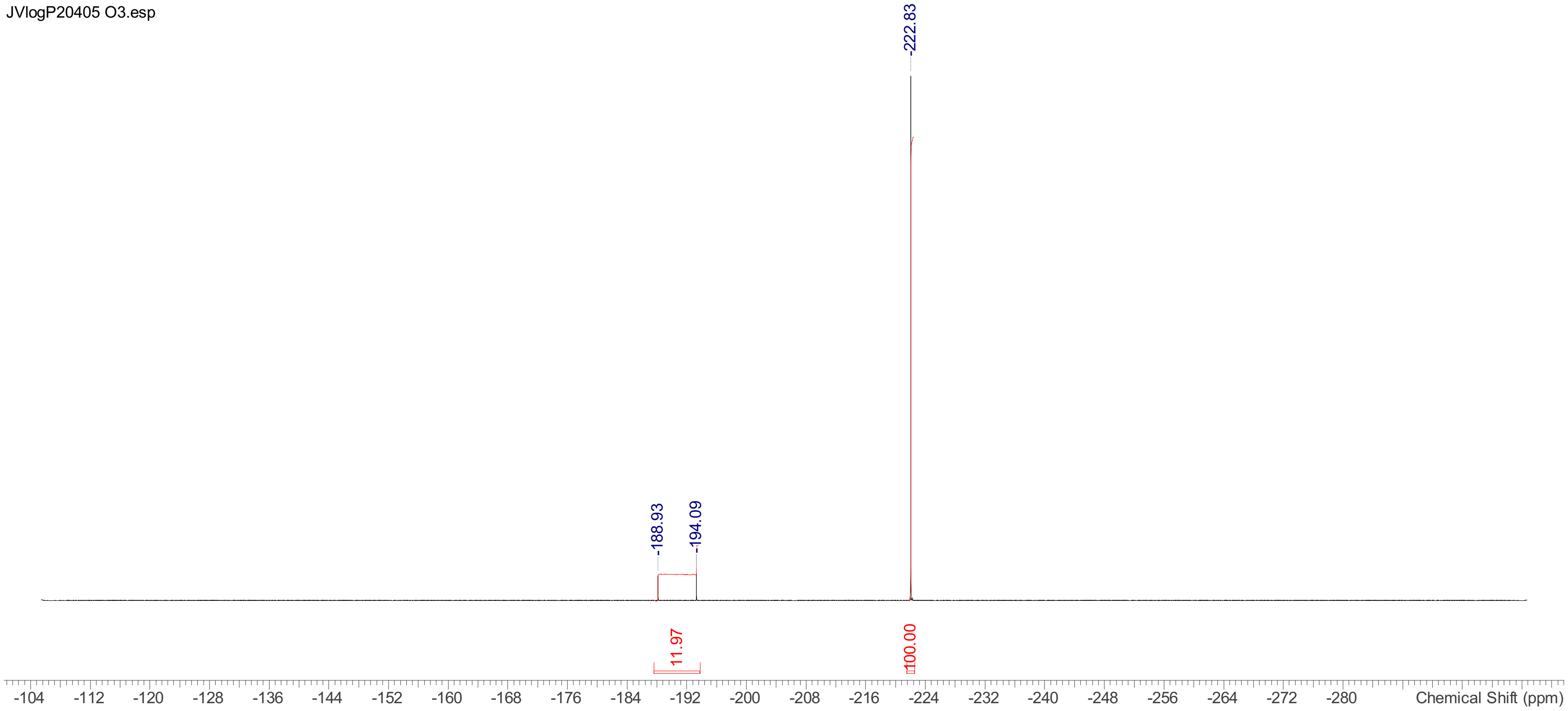

Water sample:

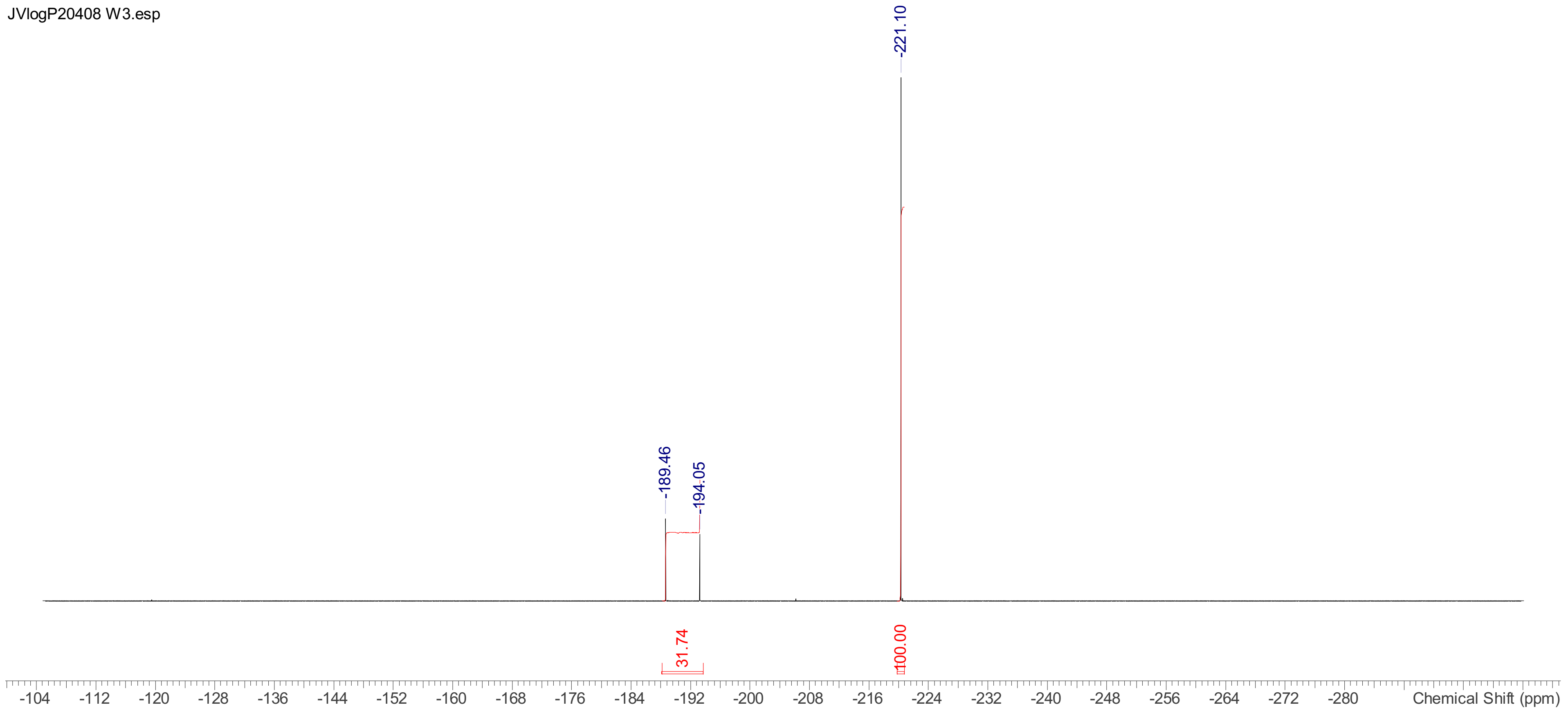

3.11 3,4-Dideoxy-4-chloro-3-fluoro-D-glucose (11)

Octanol sample:

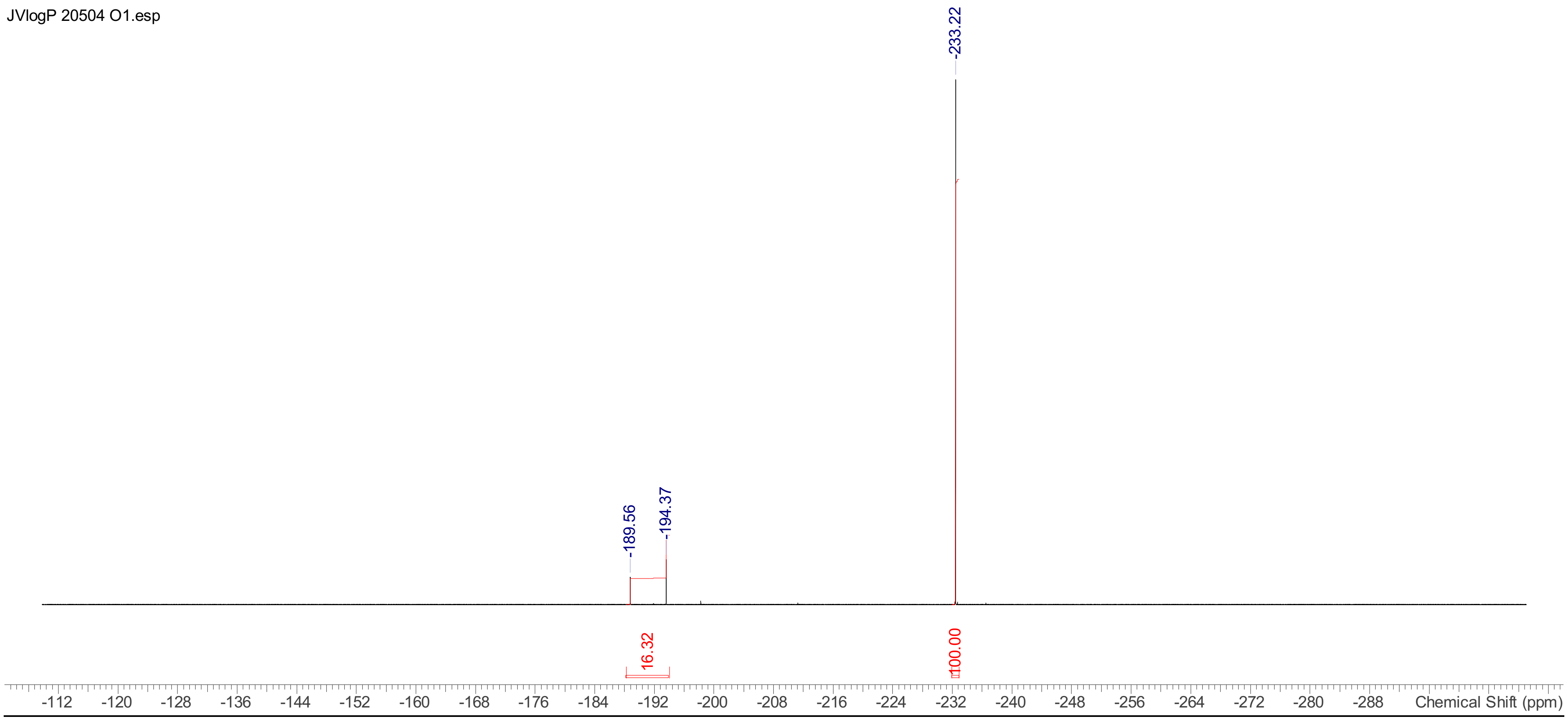

Water sample:

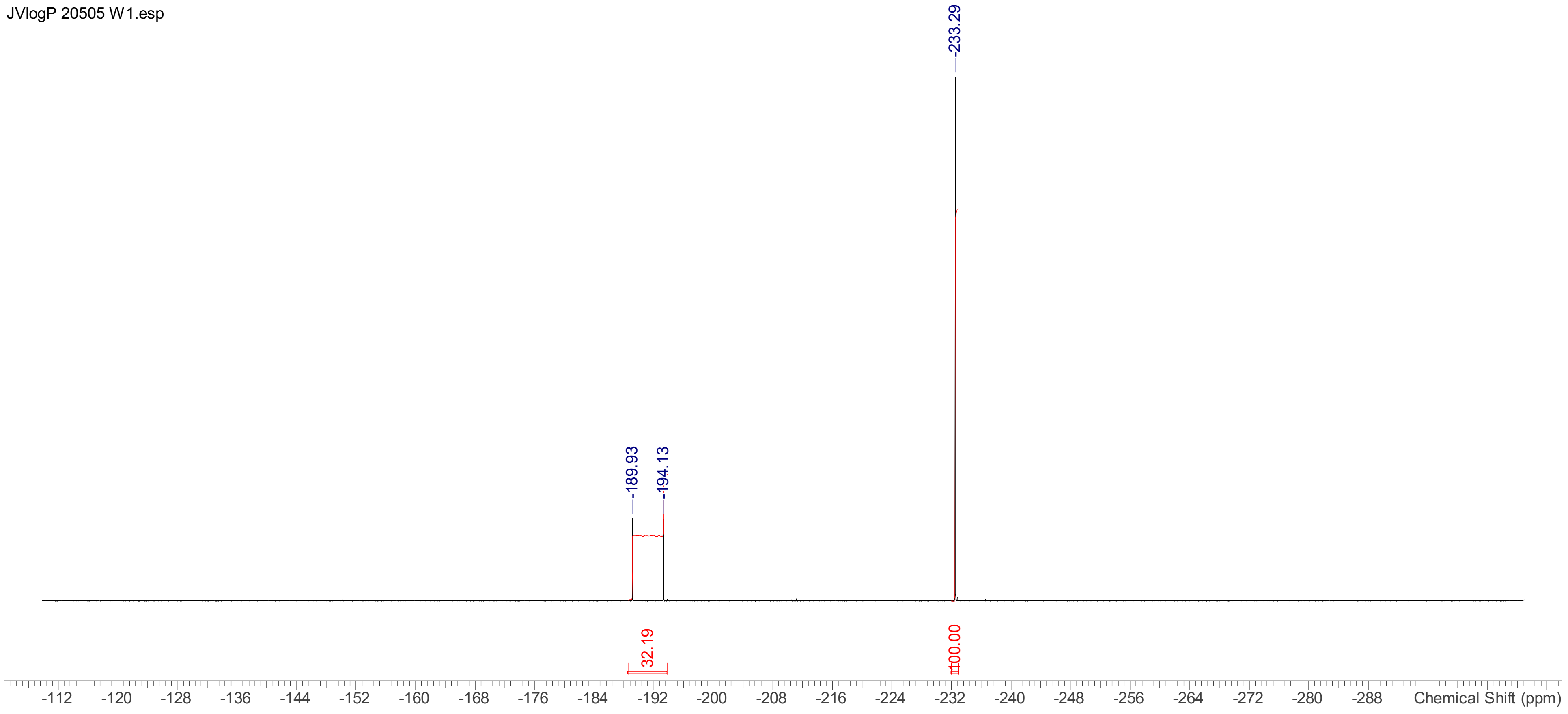

4 ^{1}H NMR spectra for known compounds synthesised by a new method

4.1 1,6-Anhydro-4-*O*-benzyl-2-deoxy-2-chloro-*β*-D-glucopyranoside (15) (400 MHz, $CDCl_3$)

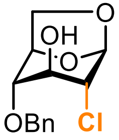

15

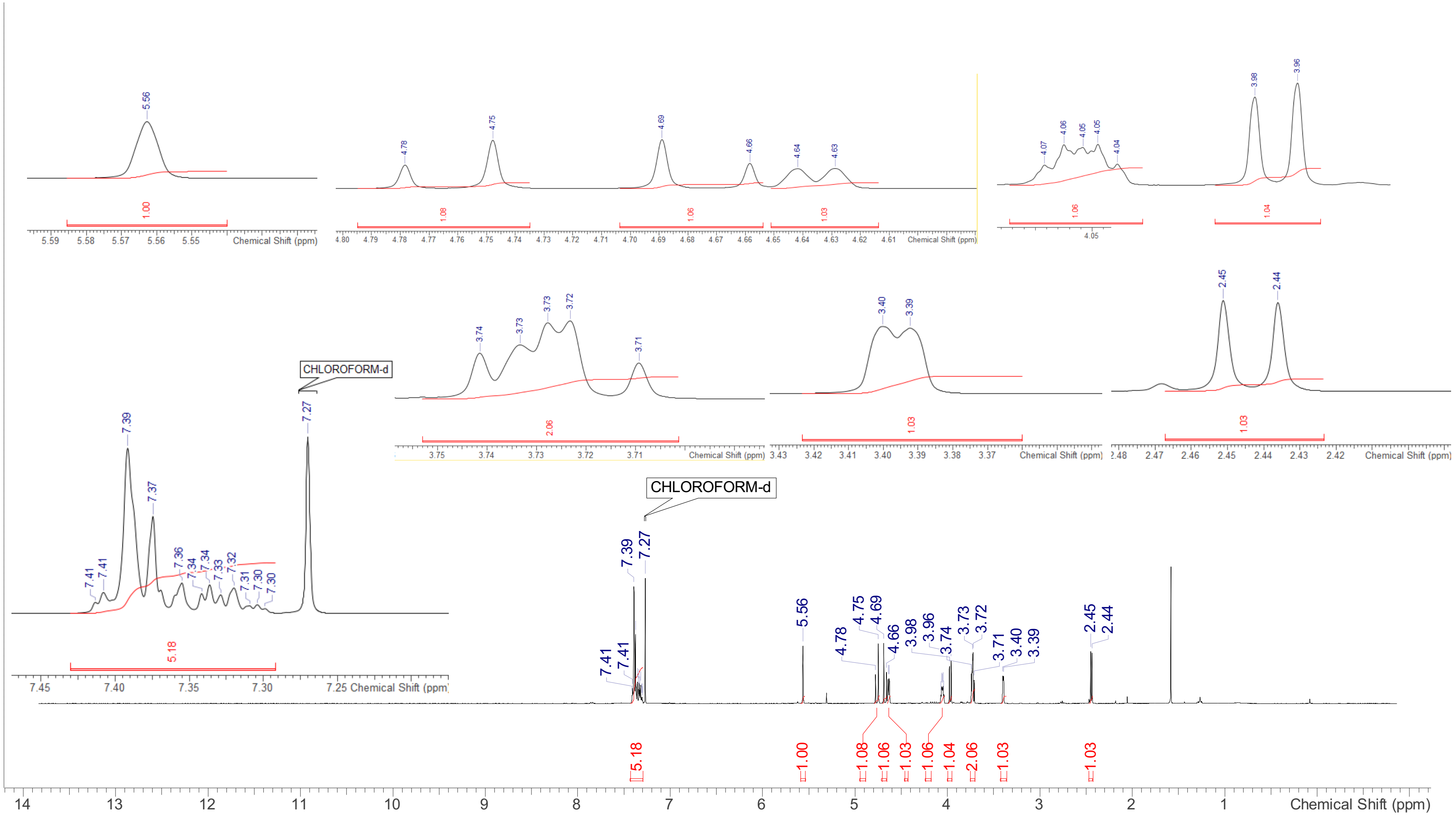

4.2 1,6-Anhydro-4-deoxy-4-chloro-2-*O*-tosyl-*β*-D-glucopyranoside (17) (400 MHz, $CDCl_3$)

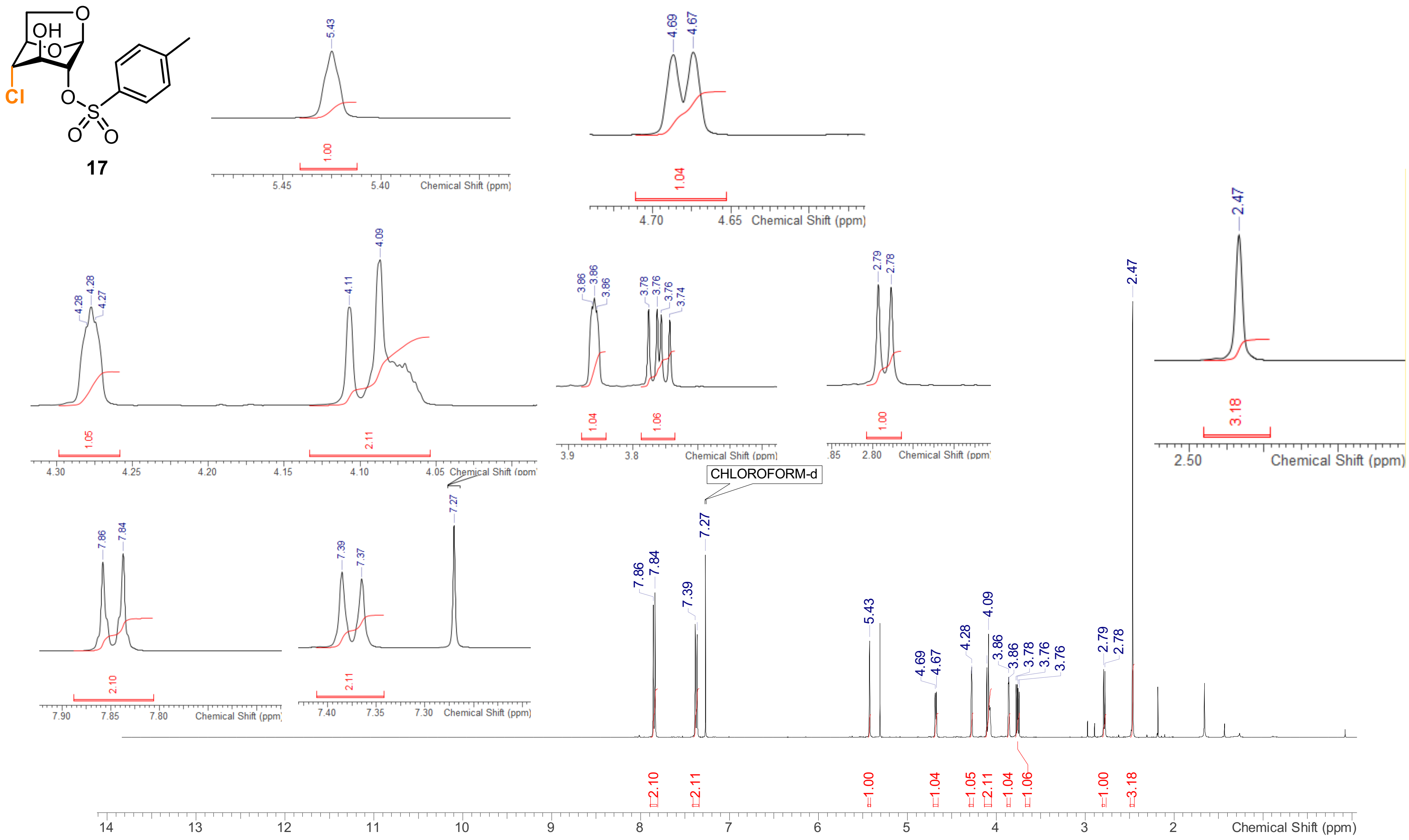

4.3 1,6;2,3-Dianhydro-4-deoxy-4-chloro-*β*-D-mannopyranoside (18) (400 MHz, $CDCl_3$)

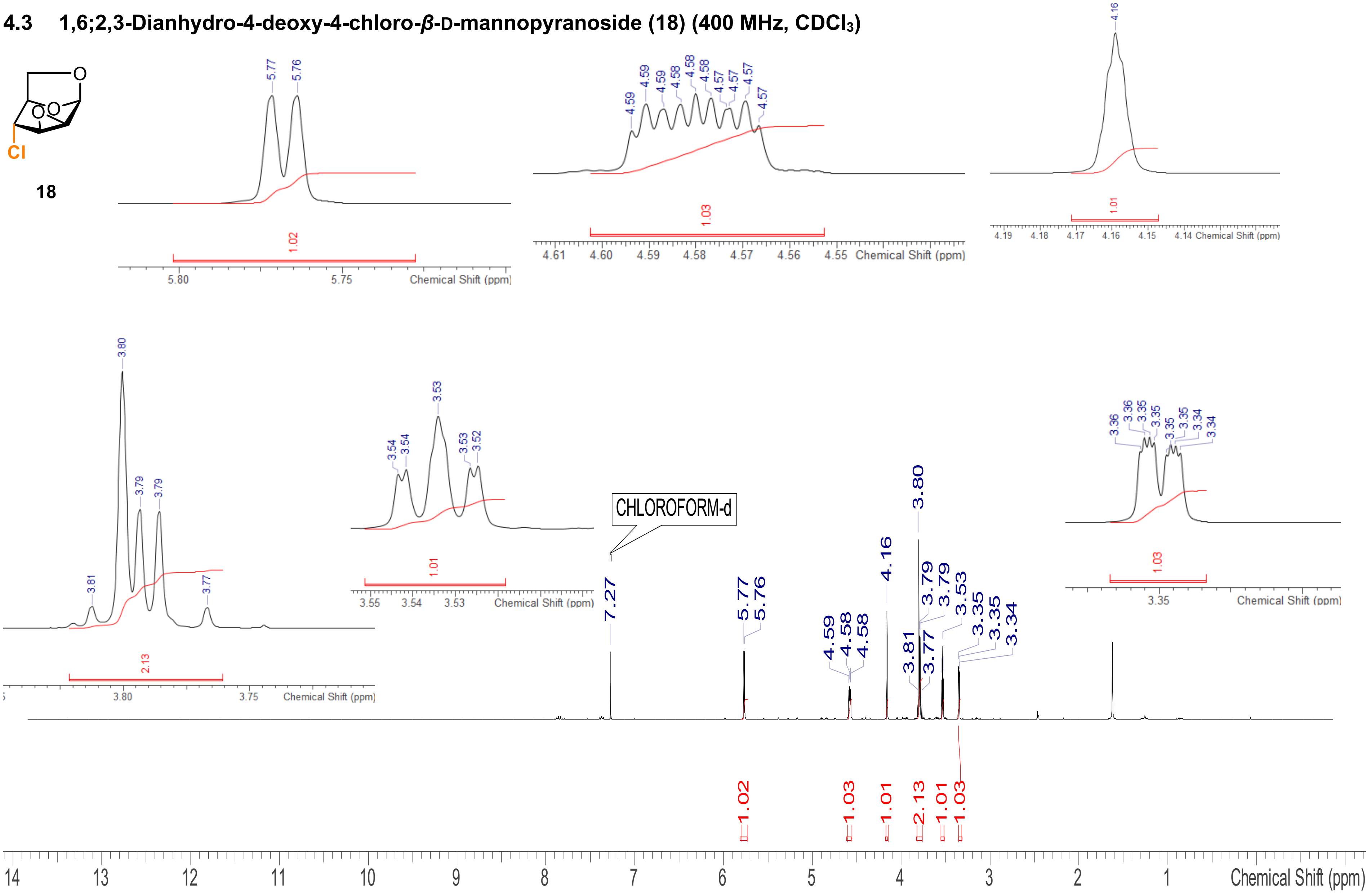

18

5 NMR spectra for novel compounds

5.1 NMR spectra of 4,6-dideoxy-4-chloro-6-fluoro-D-galactose (7a)

5.1.1 ^{1}H NMR (500 MHz, MeOH-d_4)

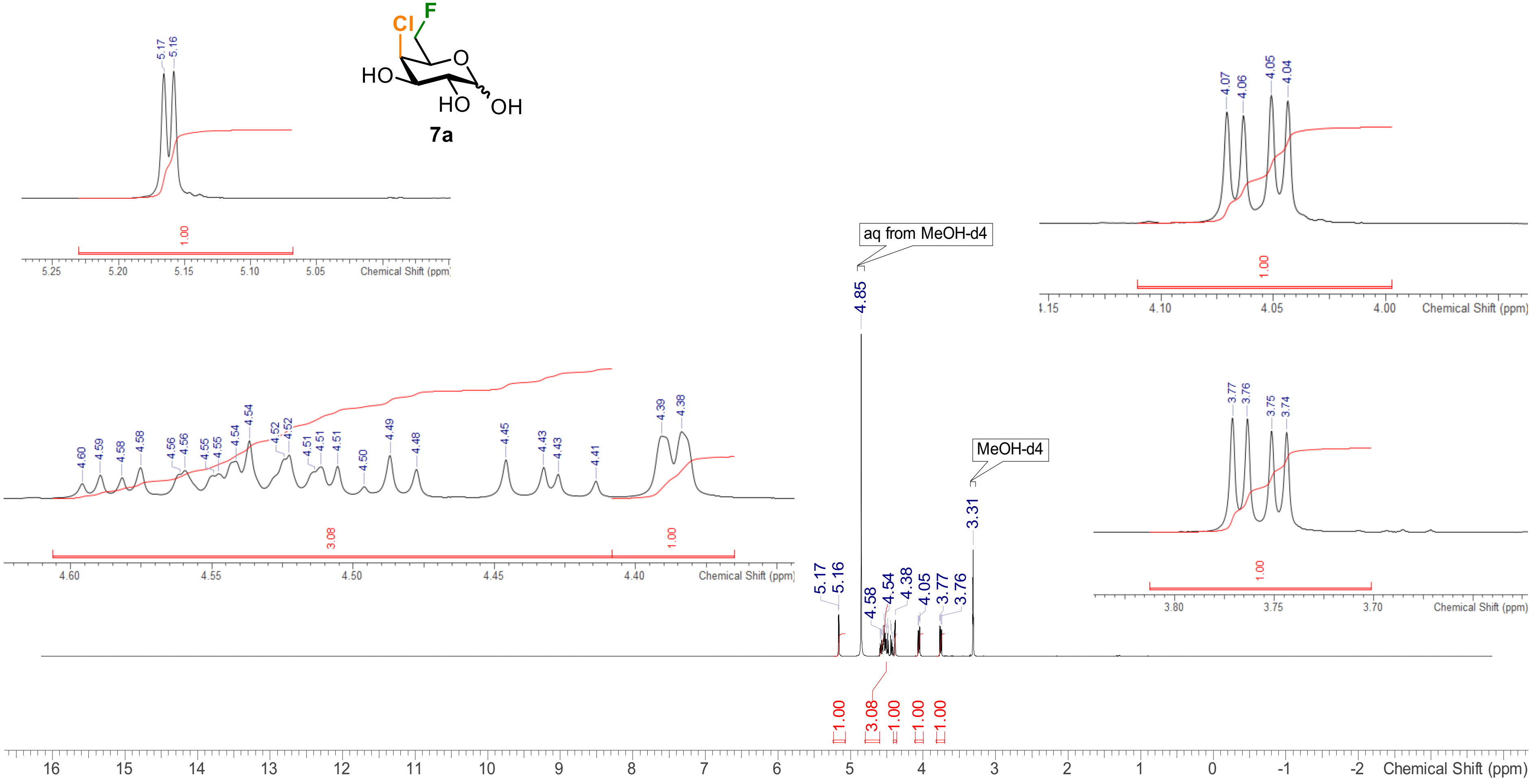

5.1.2 ^{19}F NMR (471 MHz, MeOH-d_4)

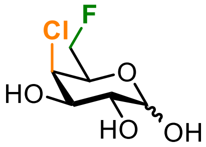

7a

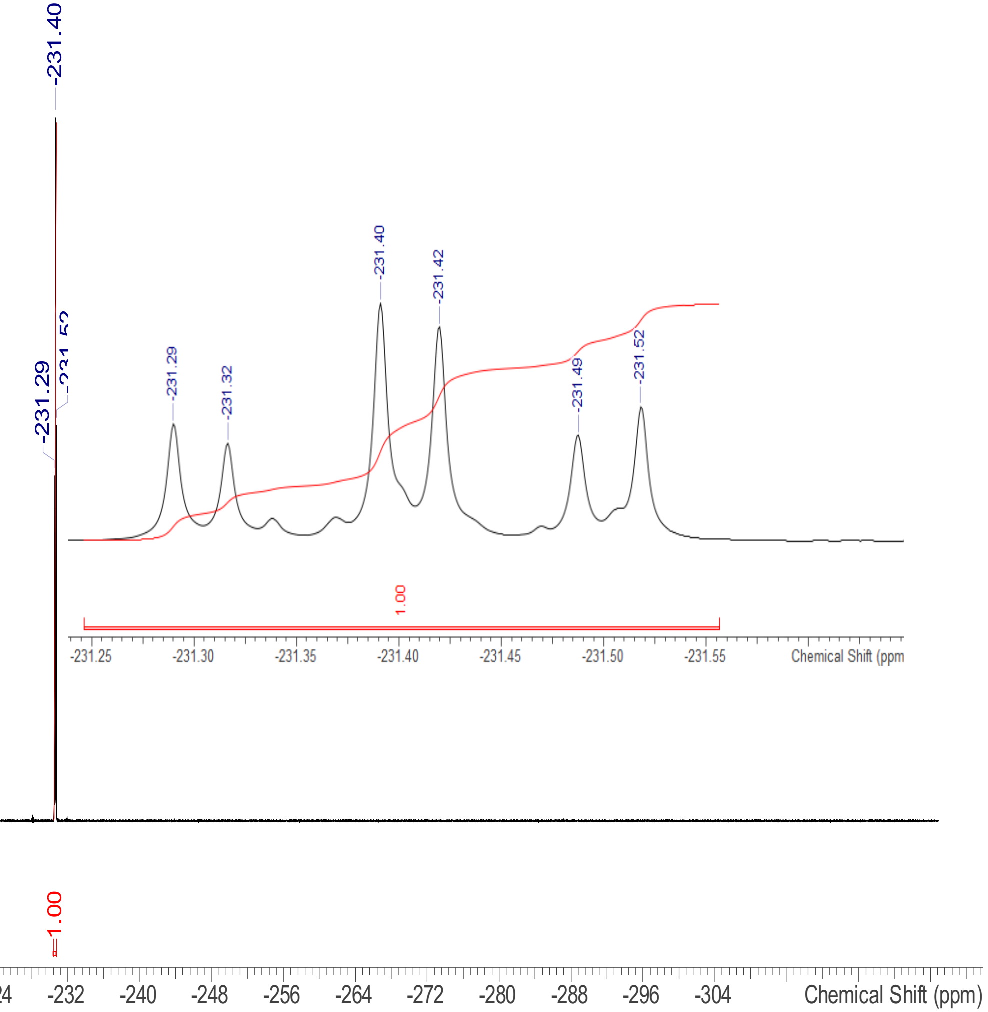

5.1.3 $^{19}F\{^{1}H\}$ NMR (471 MHz, MeOH-d_4)

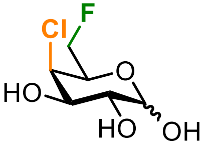

7a

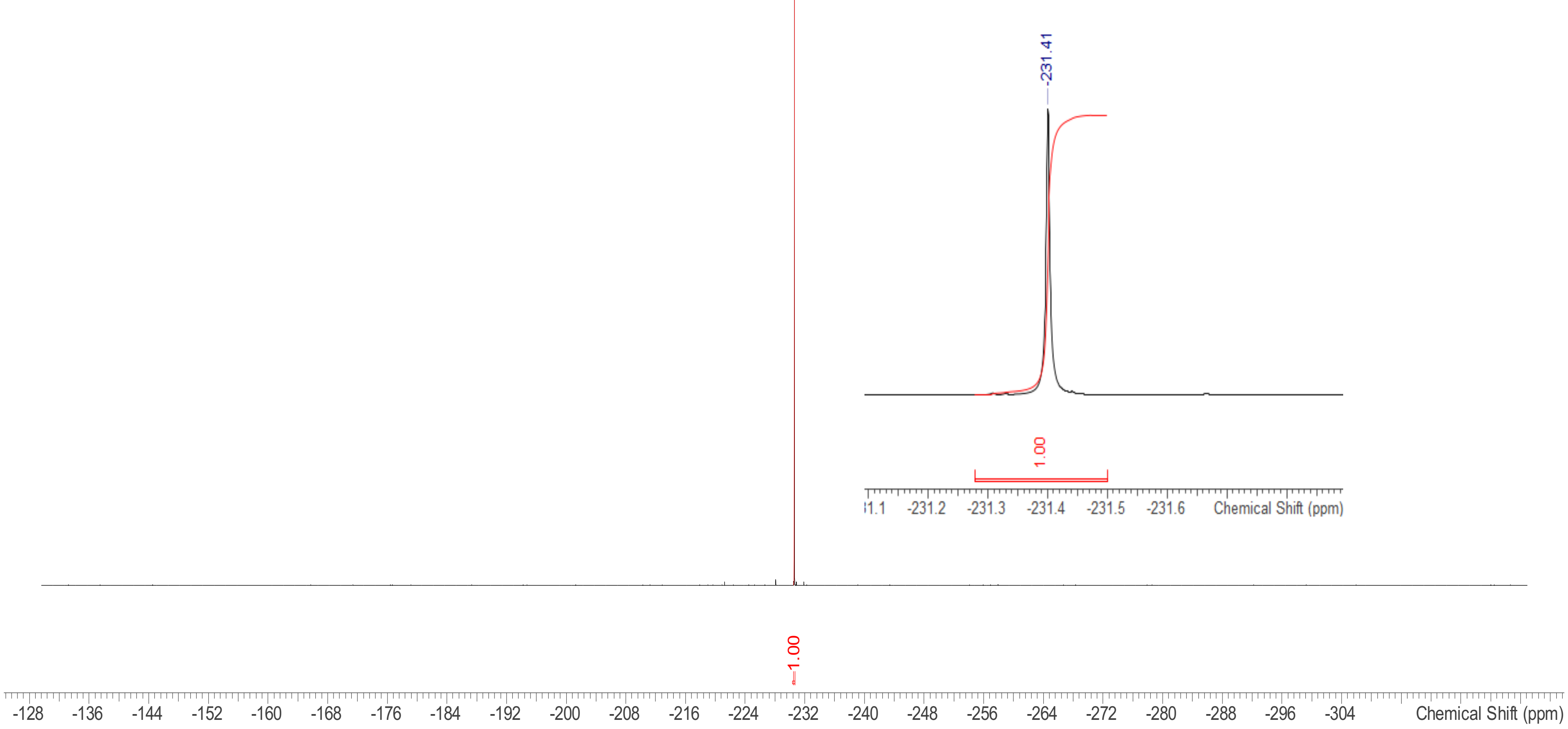

5.1.4 $^{13}C\{^1H\}$ NMR + APT (101 MHz, MeOH-d_4)

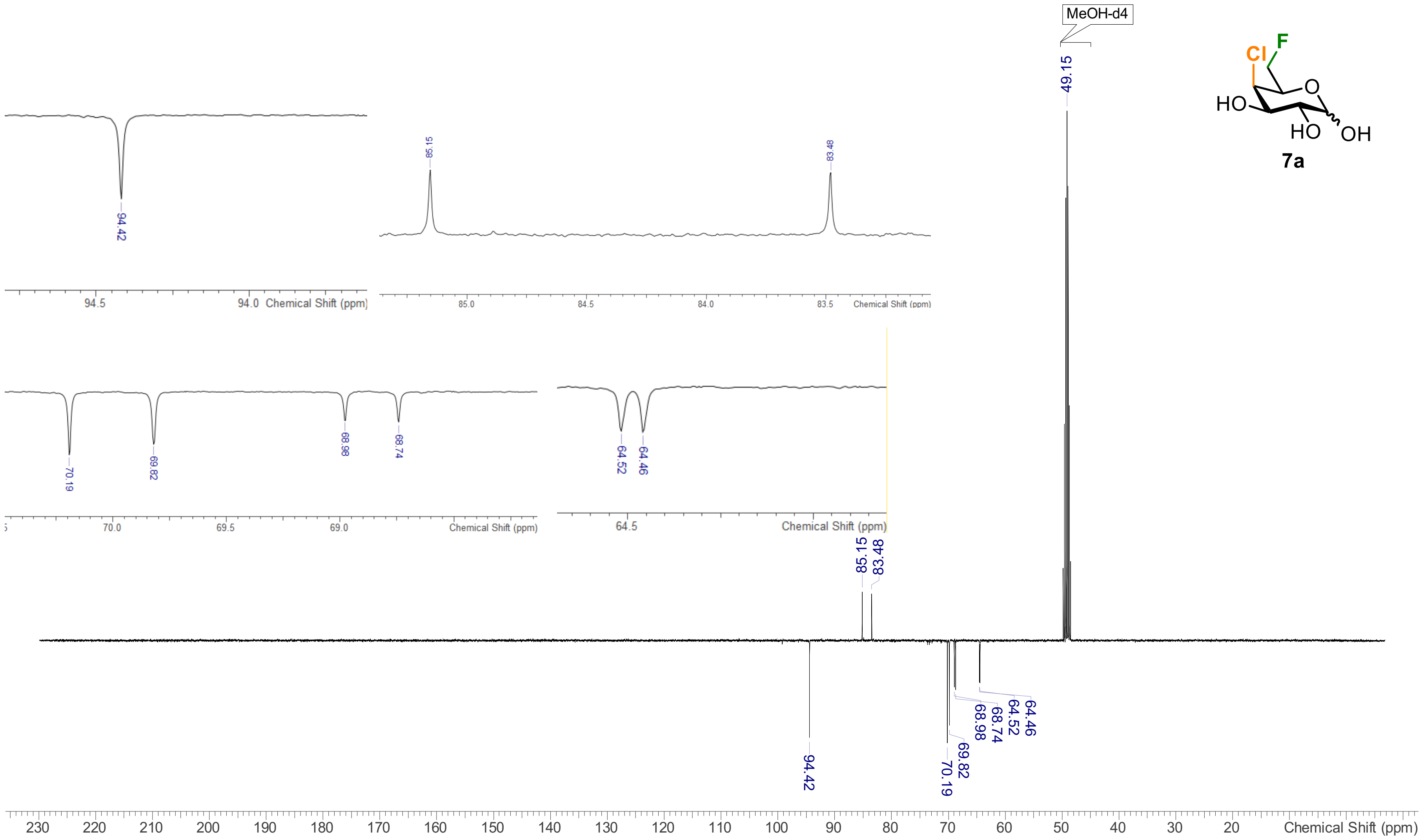

7a

5.2 NMR spectra of 4,6-dideoxy-6-chloro-4-fluoro-D-galactose (8a)

5.2.1 ^{1}H NMR (400 MHz, MeOH-d_4)

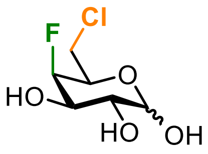

8a

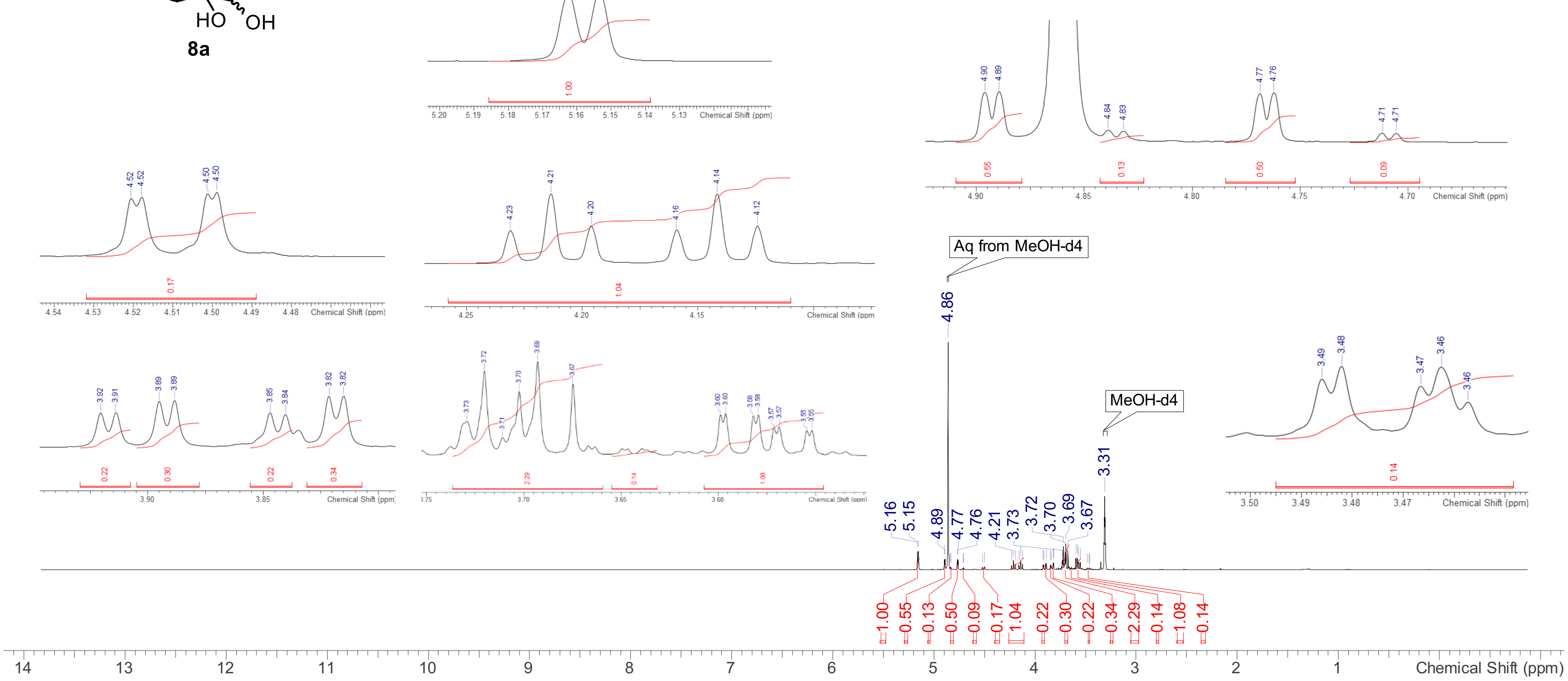

5.2.2 ^{19}F NMR (377 MHz, MeOH-d_4)

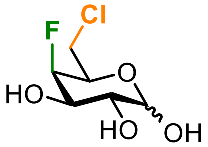

8a

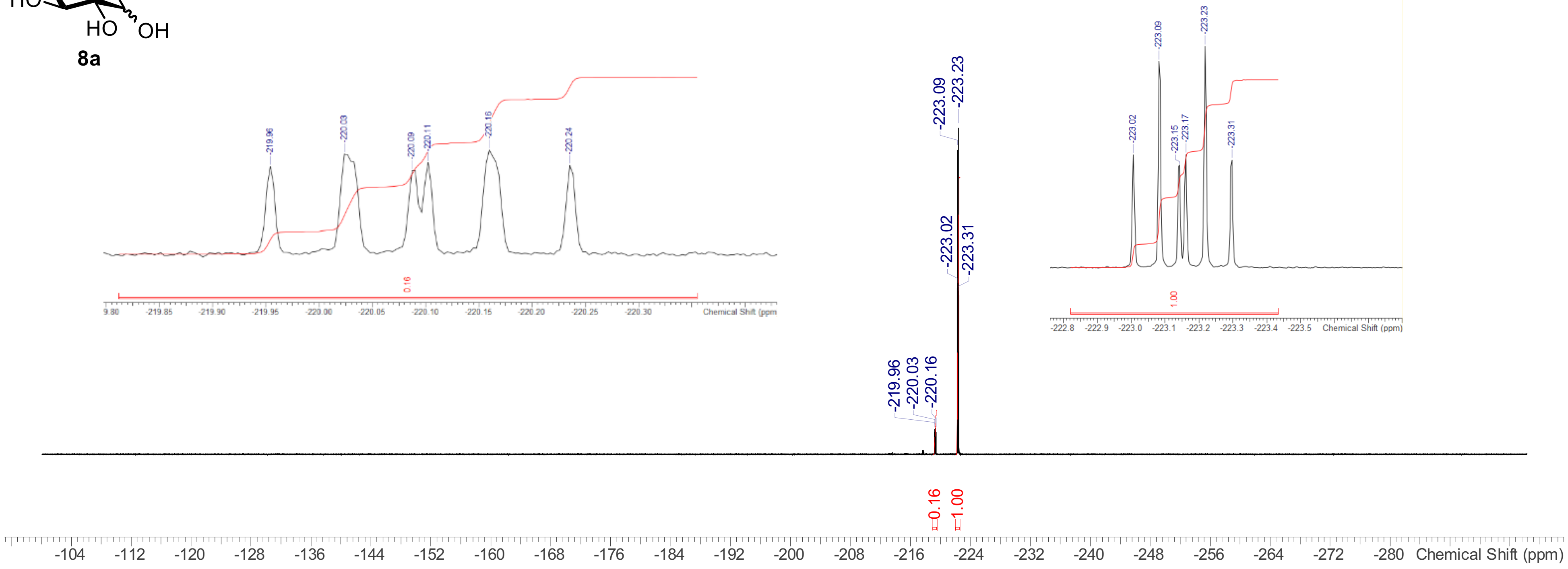

5.2.3 $^{19}F\{^{1}H\}$ NMR (471 MHz, MeOH-d_4)

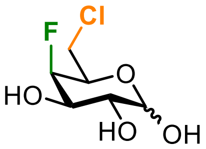

8a

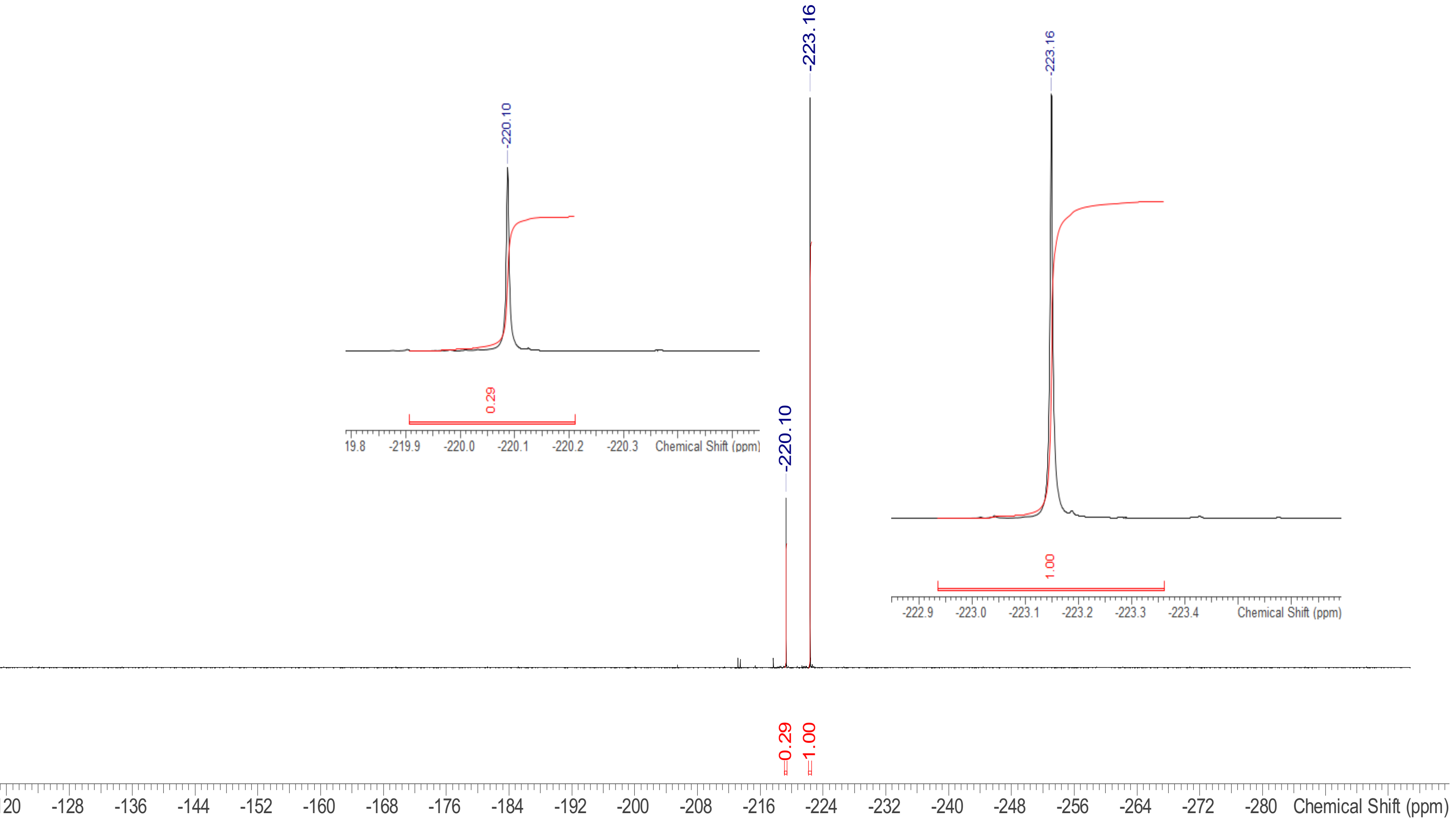

5.2.4 $^{13}C\{^{1}H\}$ NMR + APT (101 MHz, MeOH-d_4)

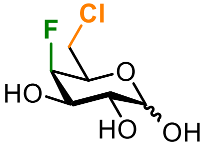

8a

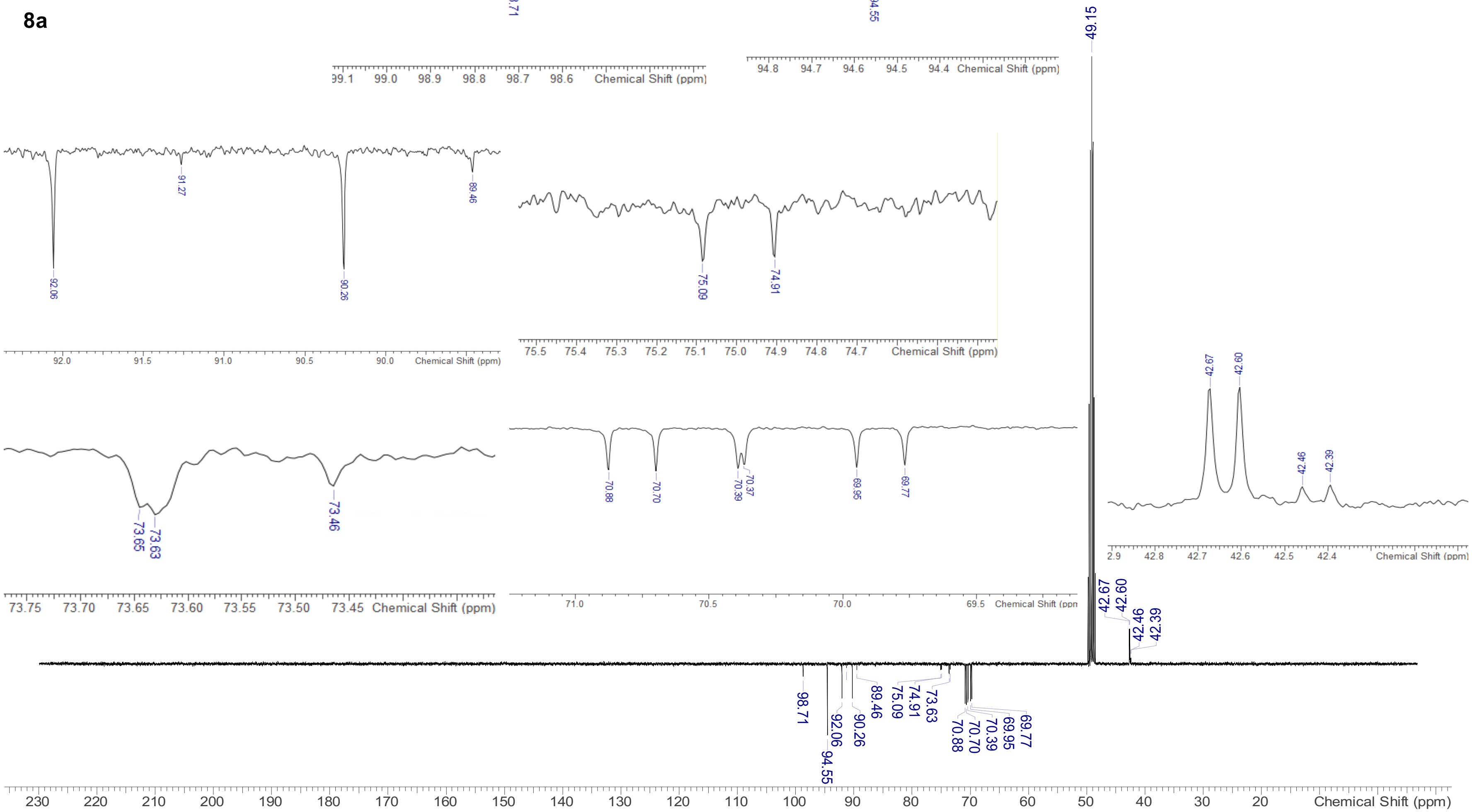

5.3 NMR spectra of 1,6-anhydro-4-*O*-benzyl-2,3-dideoxy-2-chloro-3-fluoro-β-D-glucopyranoside (16)

5.3.1 ^{1}H NMR (400 MHz, $CDCl_3$)

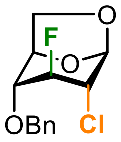

16

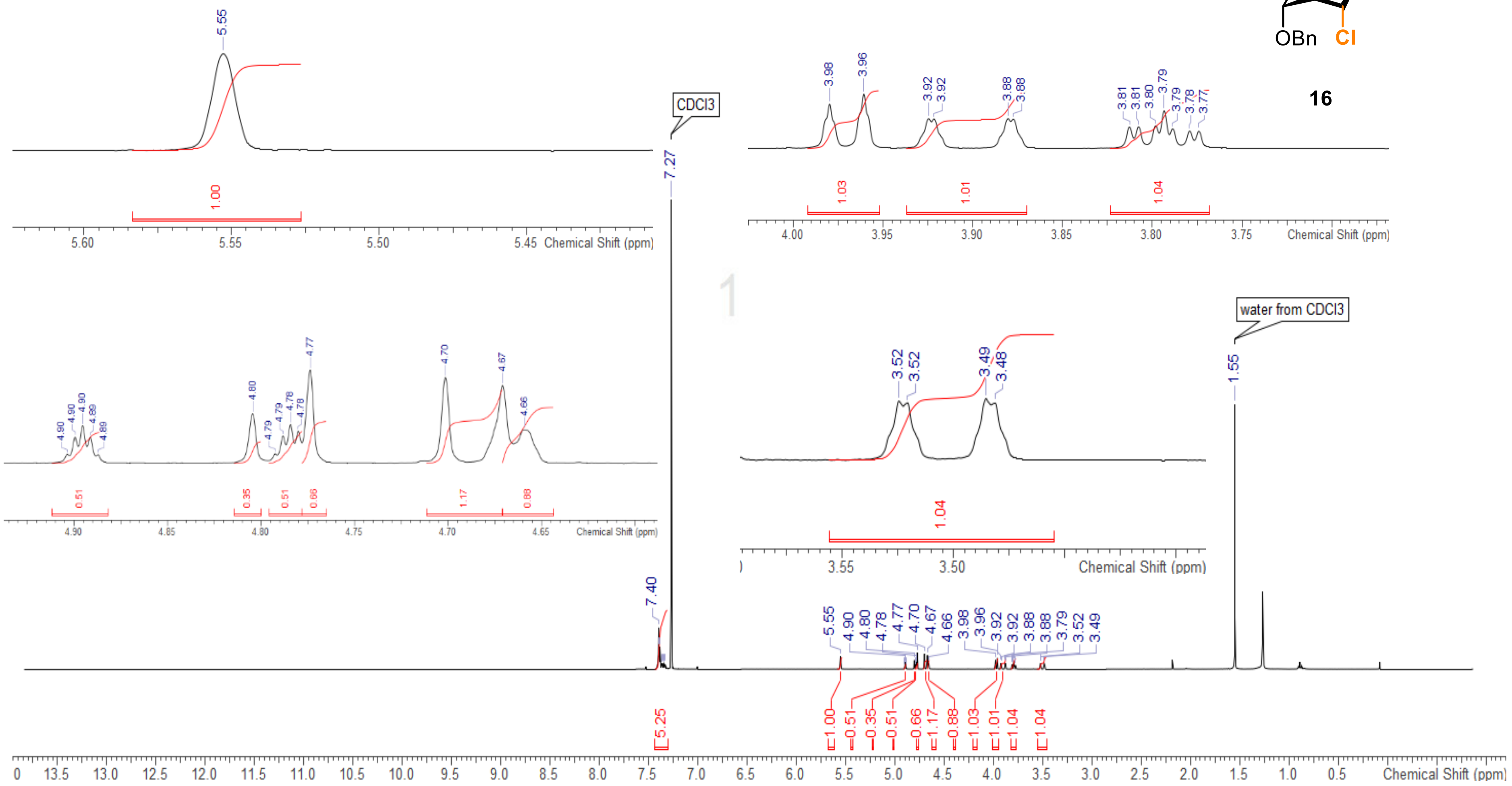

5.3.2 ^{19}F NMR (377 MHz, $CDCl_3$)

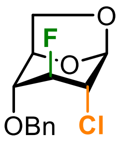

16

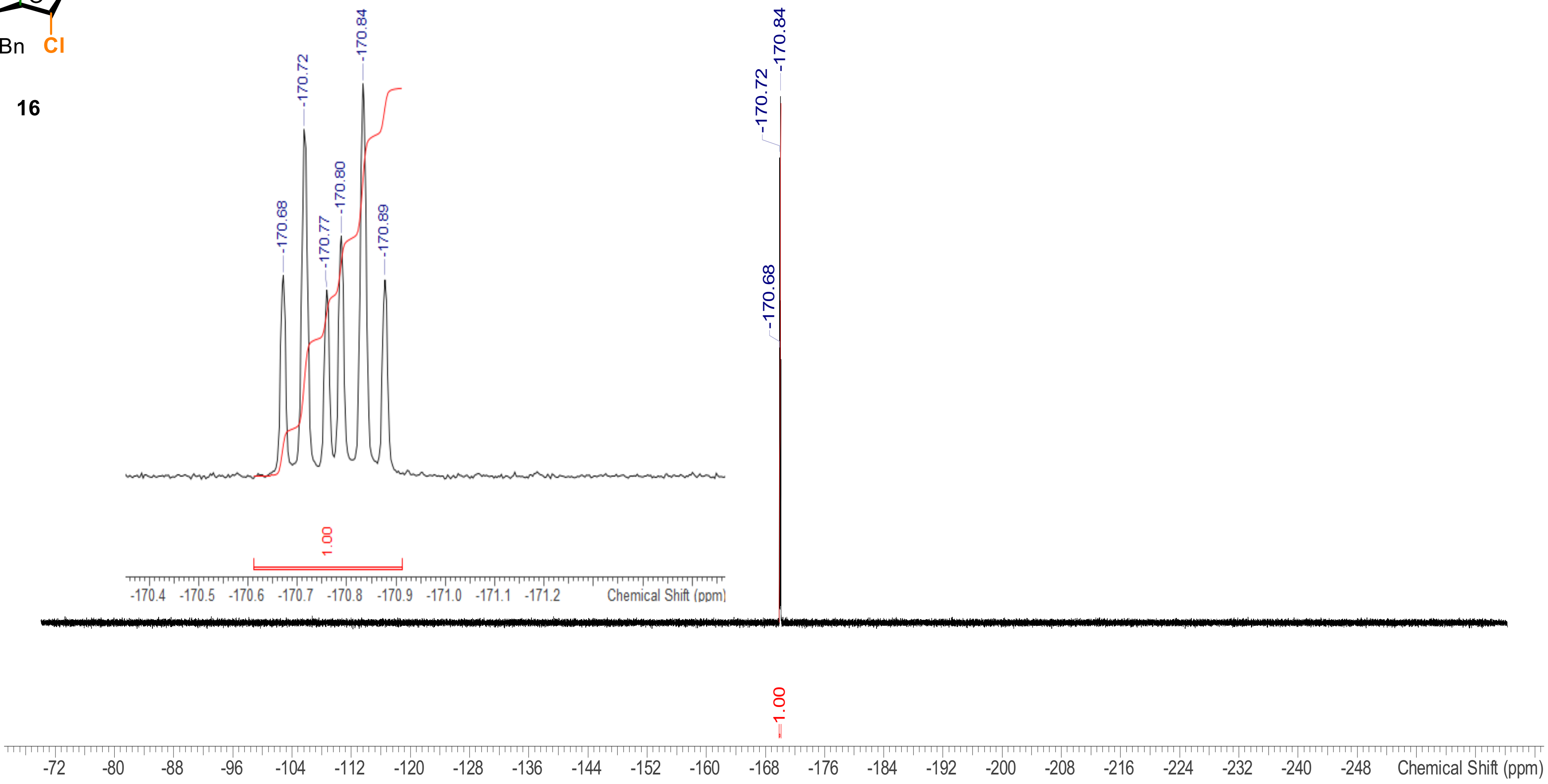

5.3.3 $^{19}F\{^{1}H\}$ NMR (471 MHz, $CDCl_3$)

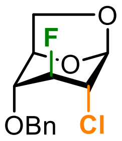

16

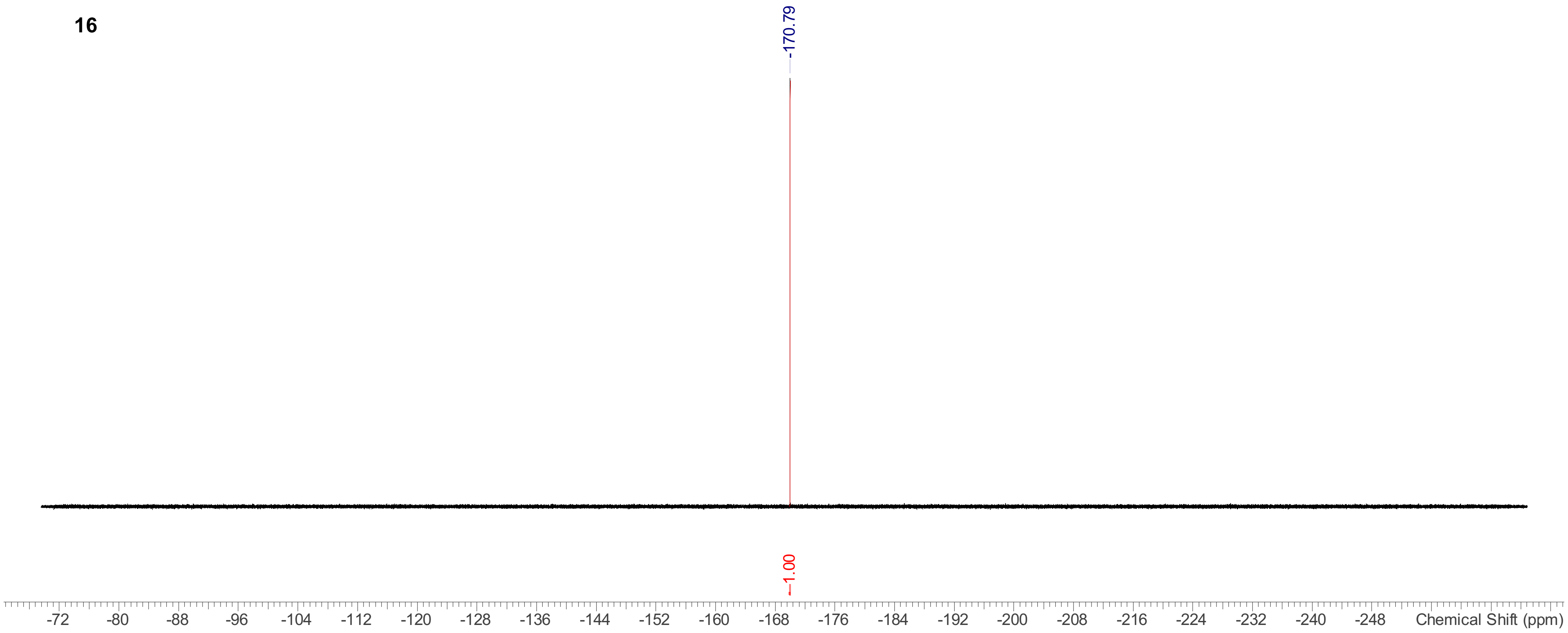

5.3.4 $^{13}C\{^{1}H\}$ NMR + APT (101 MHz, $CDCl_3$)

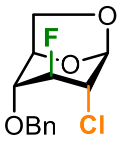

16

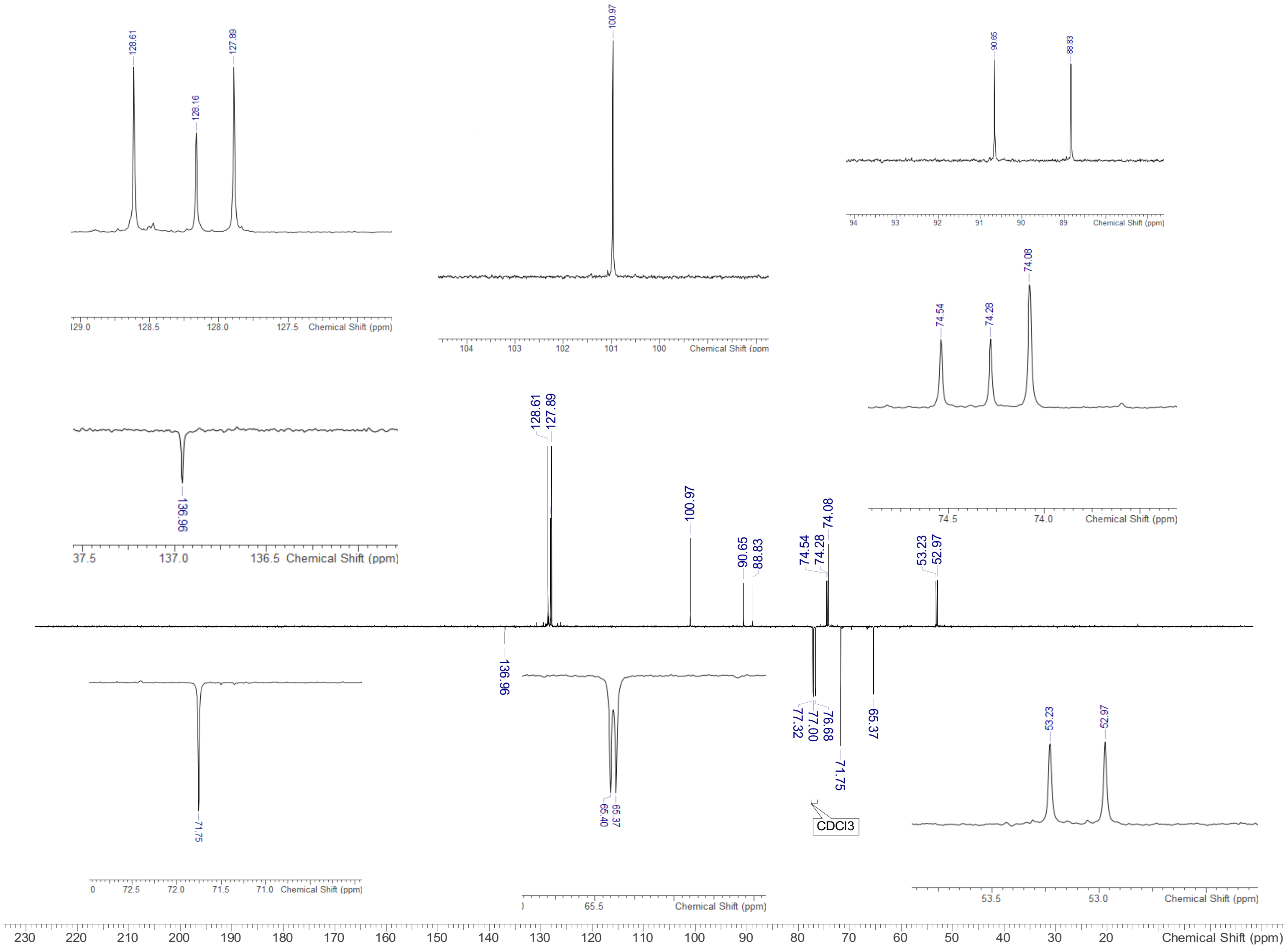

5.4 NMR spectra of 2,3-dideoxy-2-chloro-3-fluoro-D-glucose (9)

5.4.1 ^{1}H NMR (400 MHz, acetone-d_6)

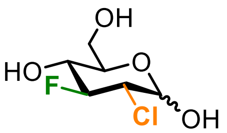

9

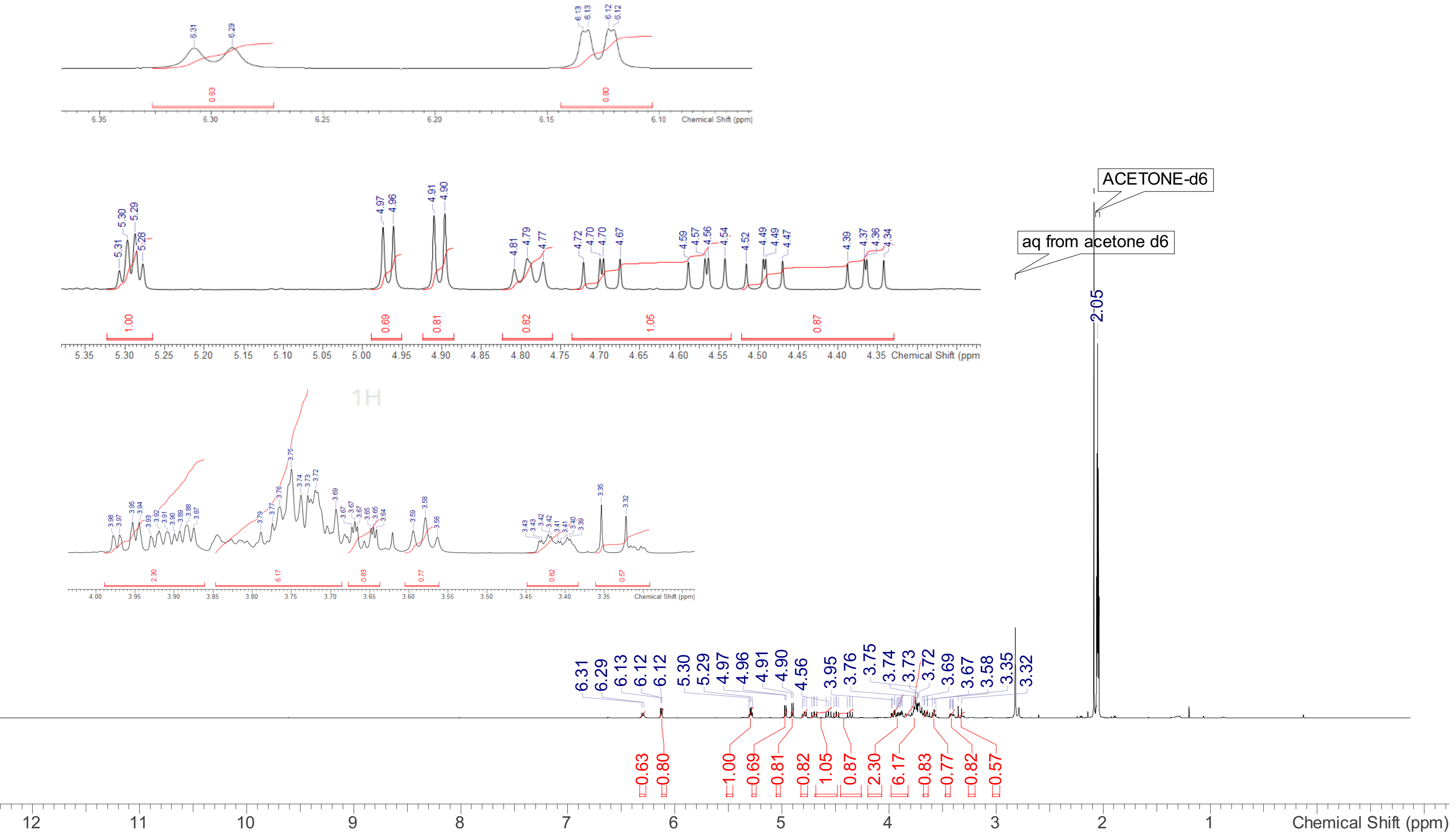

5.4.2 ^{19}F NMR (377 MHz, acetone-d_6)

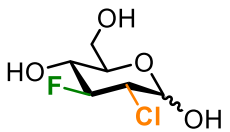

9

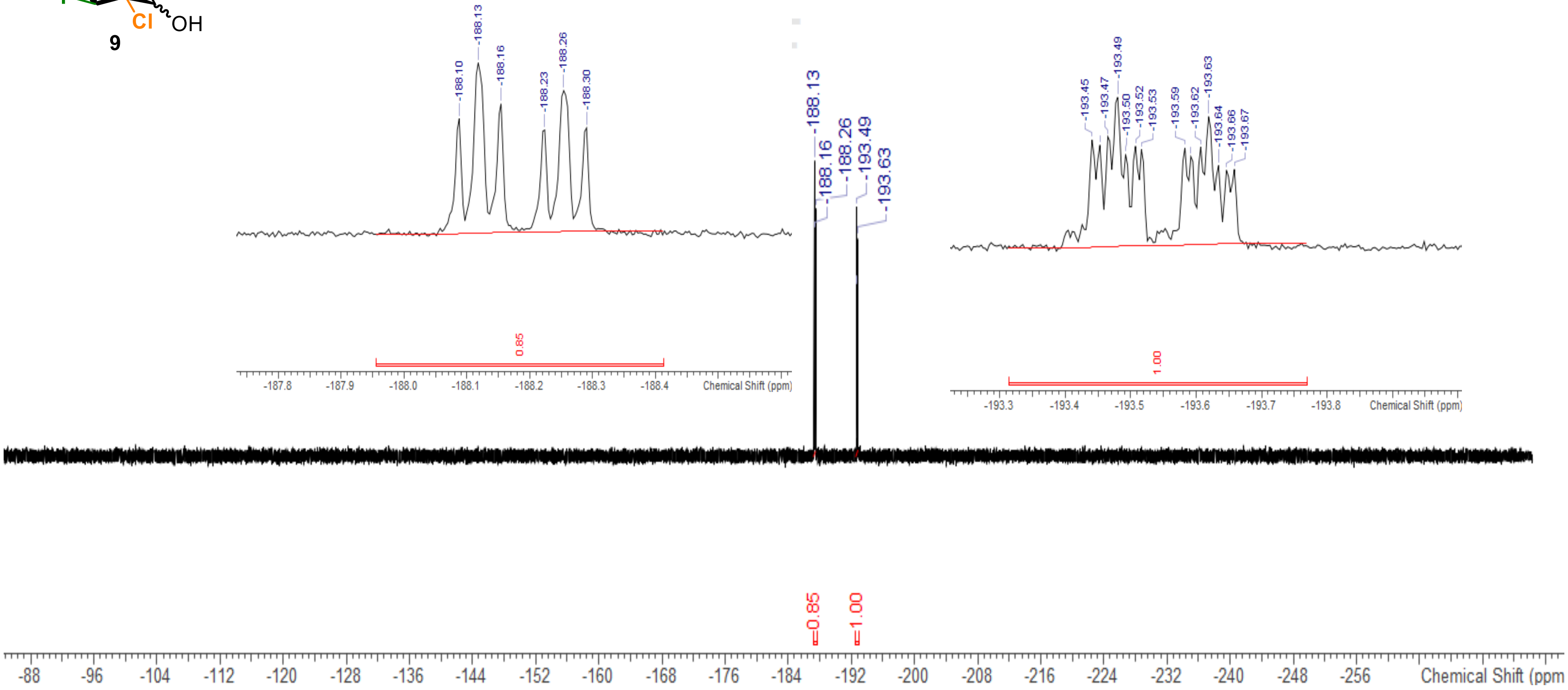

5.4.3 $^{19}F\{^{1}H\}$ NMR (471 MHz, acetone-d_6)

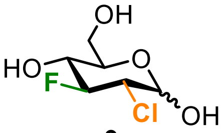

9

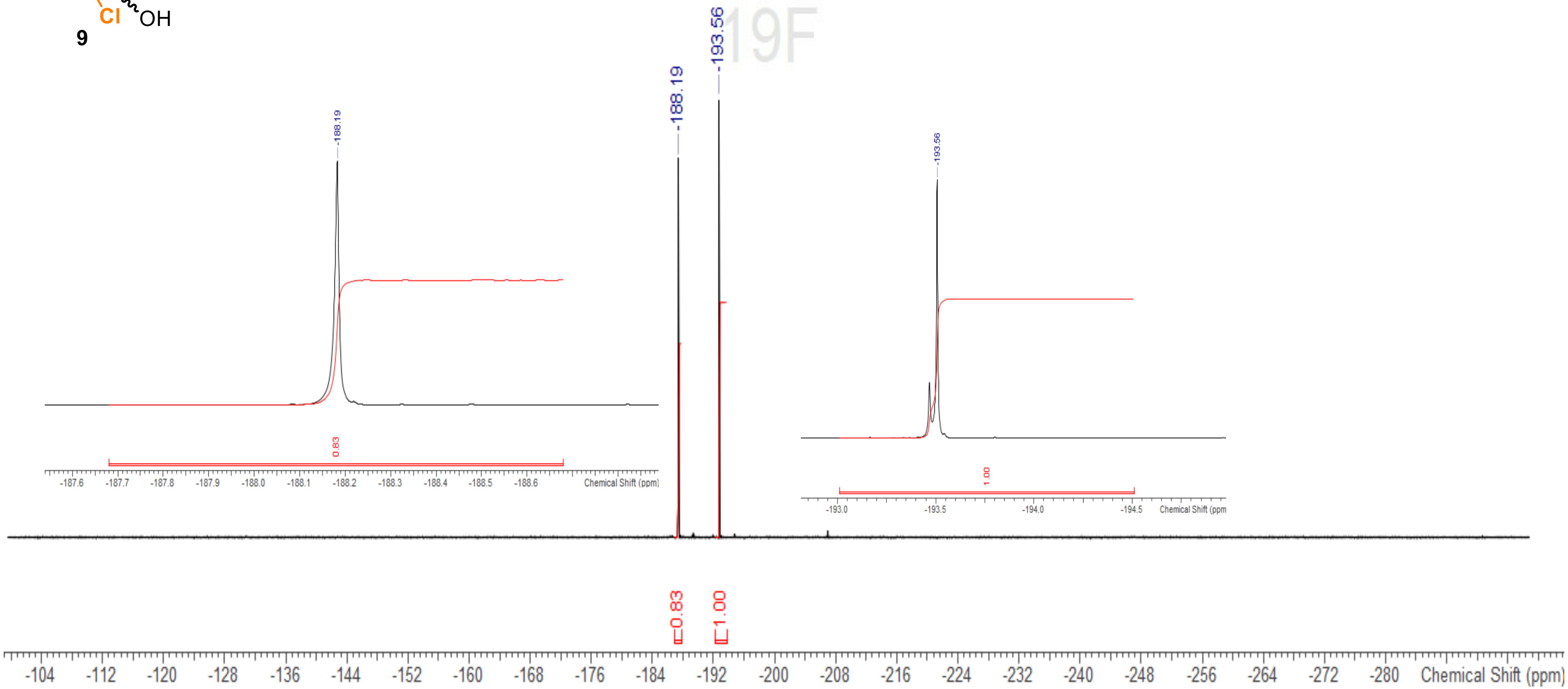

5.4.4 $^{13}C\{^{1}H\}$ NMR (101 MHz, acetone-d_6)

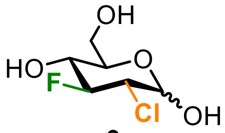

9

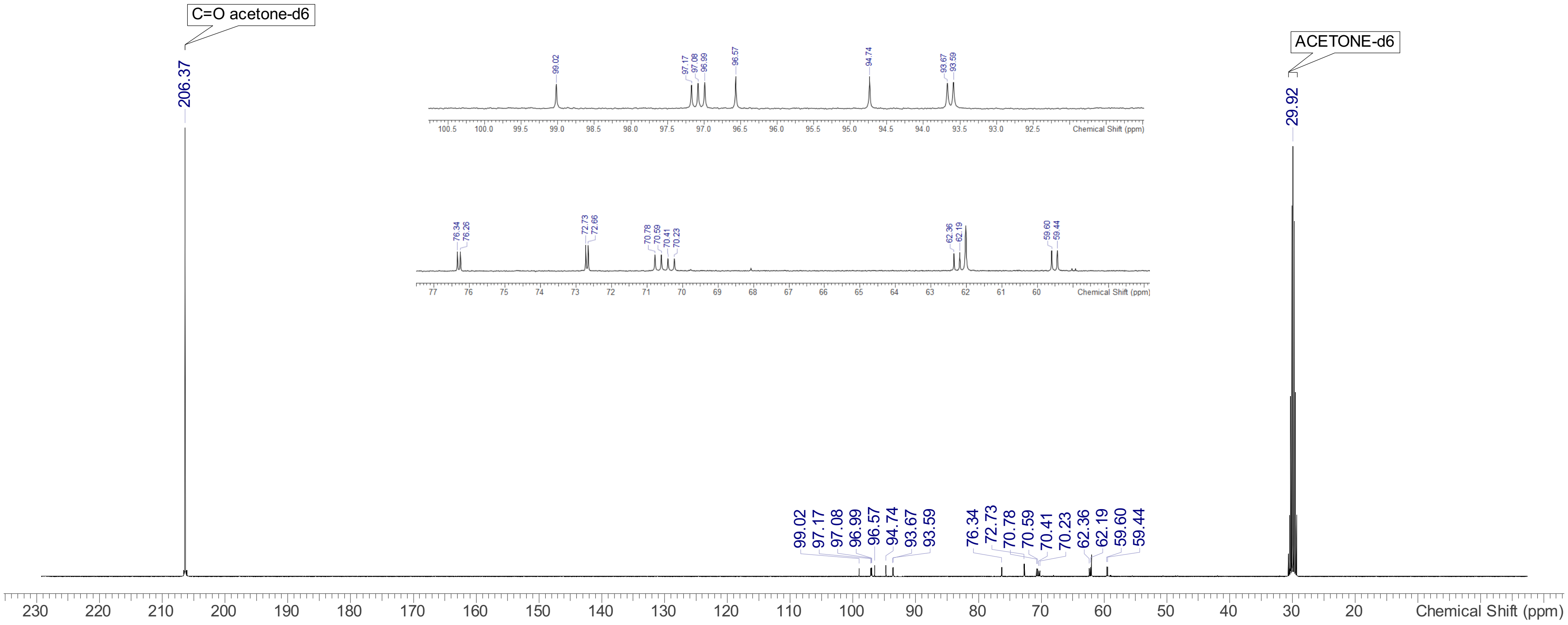

5.5 NMR spectra of 1,6-anhydro-2-*O*-benzyl-4-deoxy-4-chloro-β-D-glucopyranoside (19)

5.5.1 ^{1}H NMR (400 MHz, $CDCl_3$)

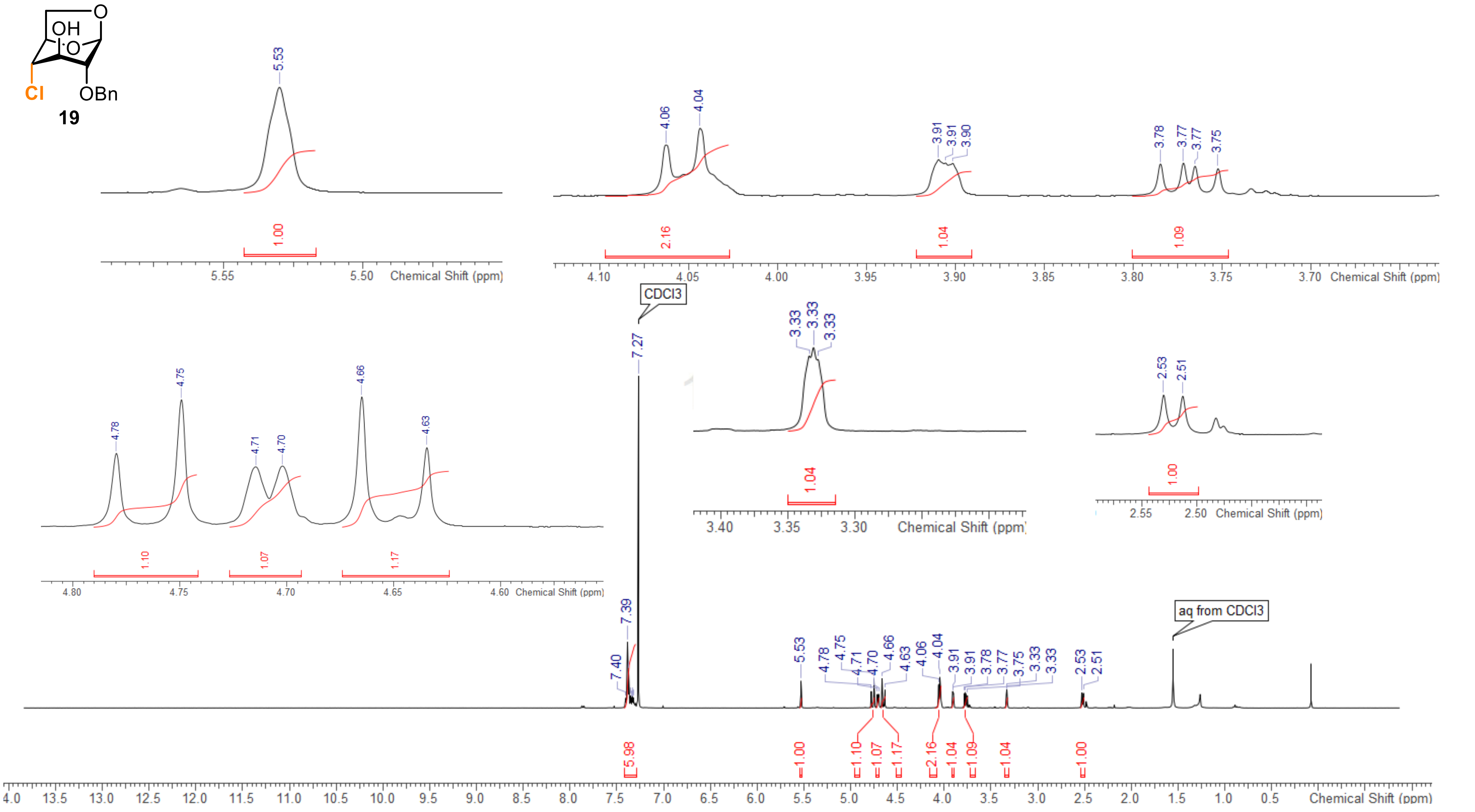

5.5.2 $^{13}C\{^{1}H\}$ NMR + APT (101 MHz, $CDCl_3$)

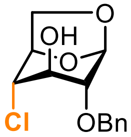

19

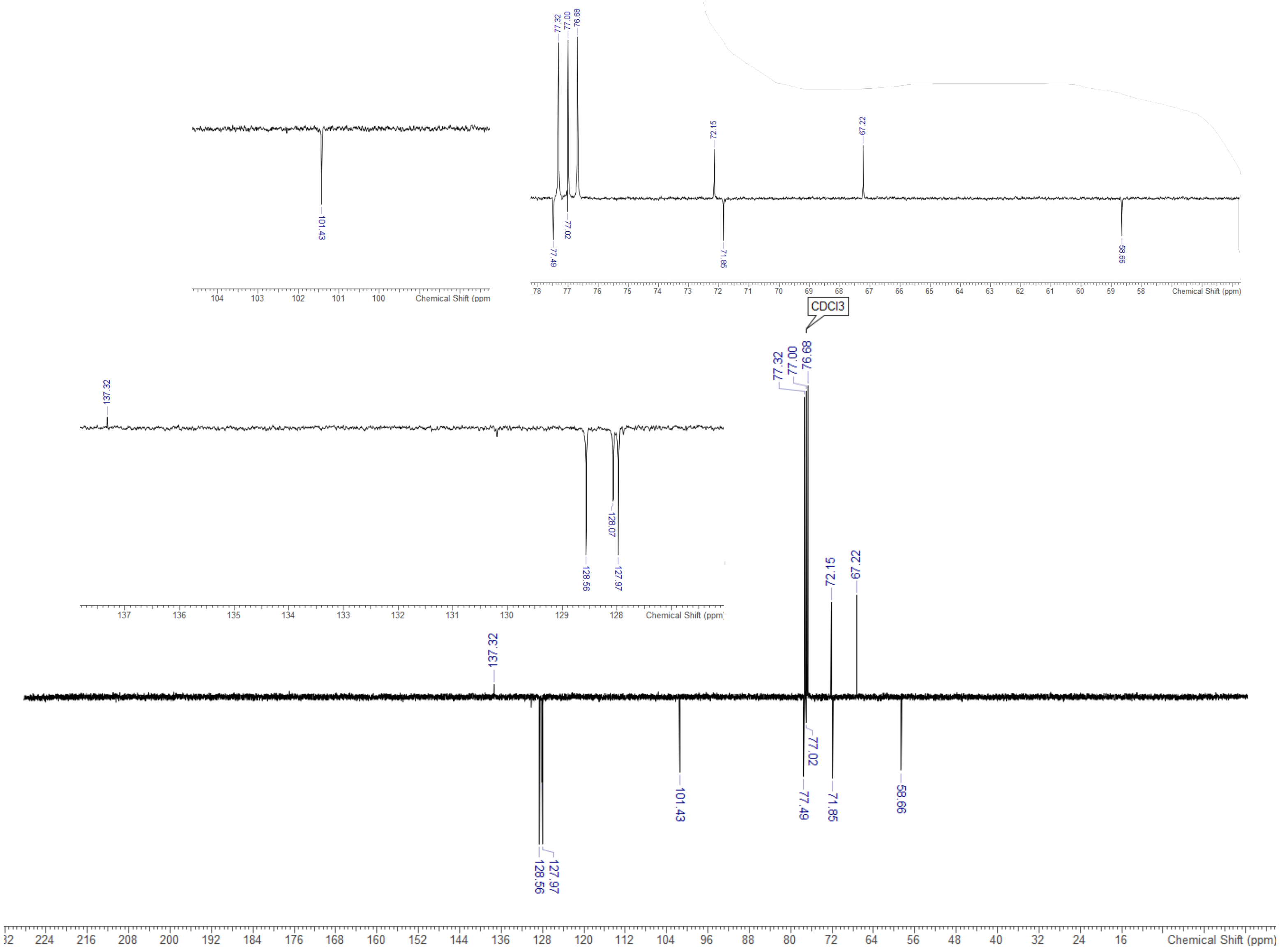

5.6 NMR spectra of 1,6-anhydro-2-*O*-benzyl-3,4-dideoxy-4-chloro-3-fluoro-β-D-glucopyranoside (20)

5.6.1 ^{1}H NMR (400 MHz, $CDCl_3$)

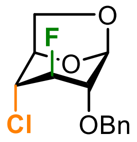

20

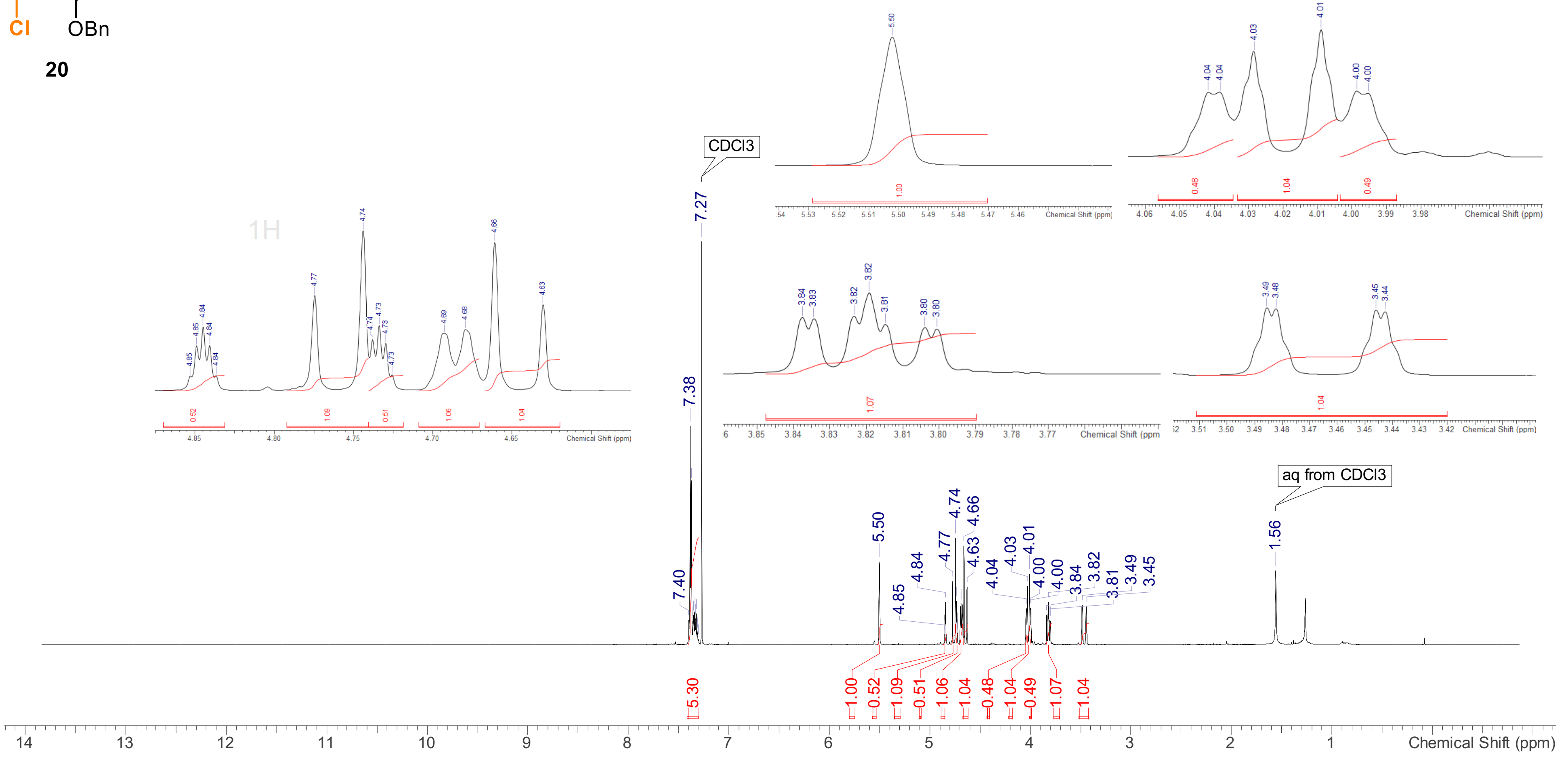

5.6.2 ^{19}F NMR (377 MHz, $CDCl_3$)

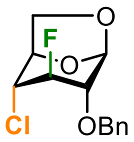

20

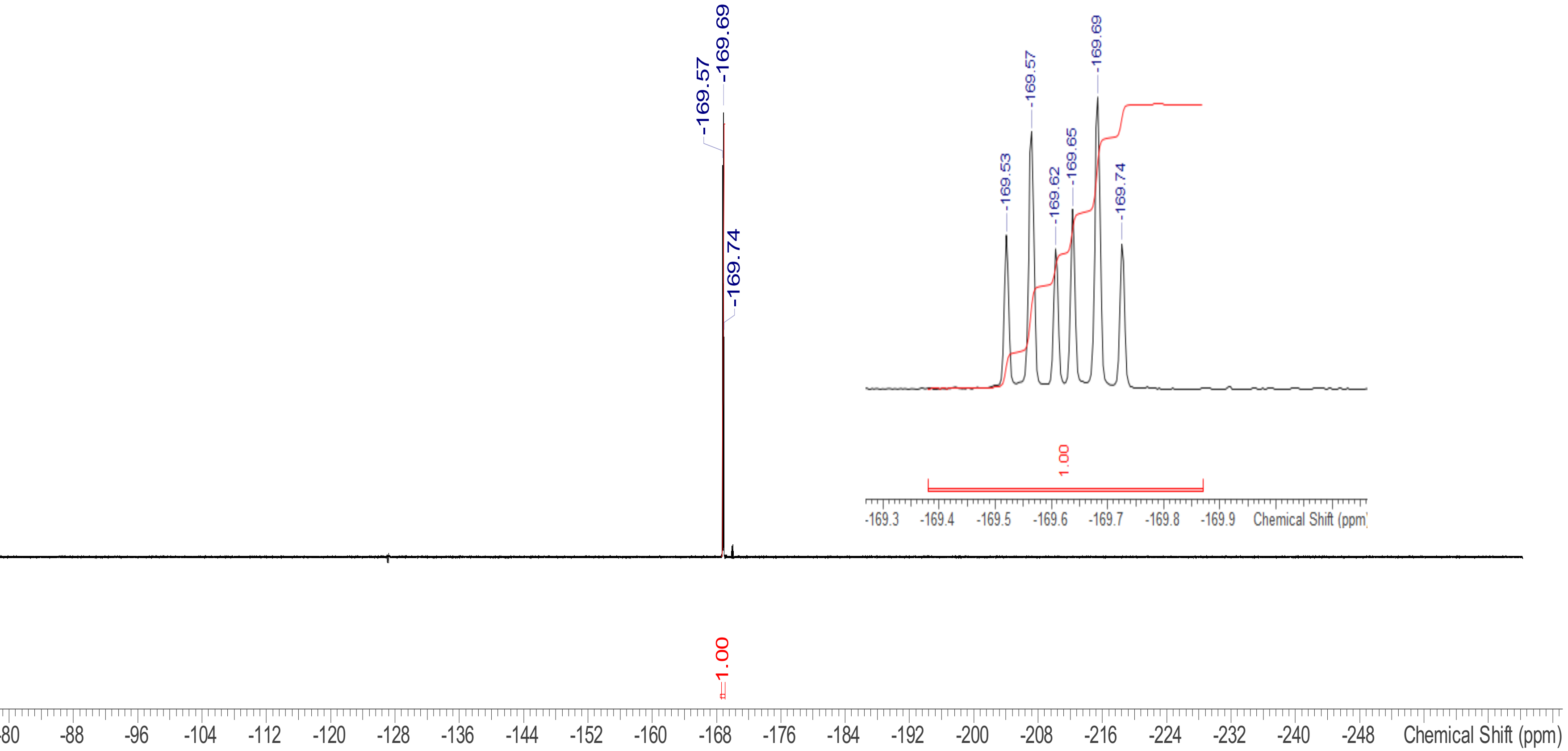

5.6.3 $^{19}F\{^{1}H\}$ NMR (471 MHz, $CDCl_3$)

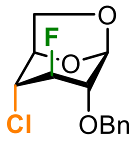

20

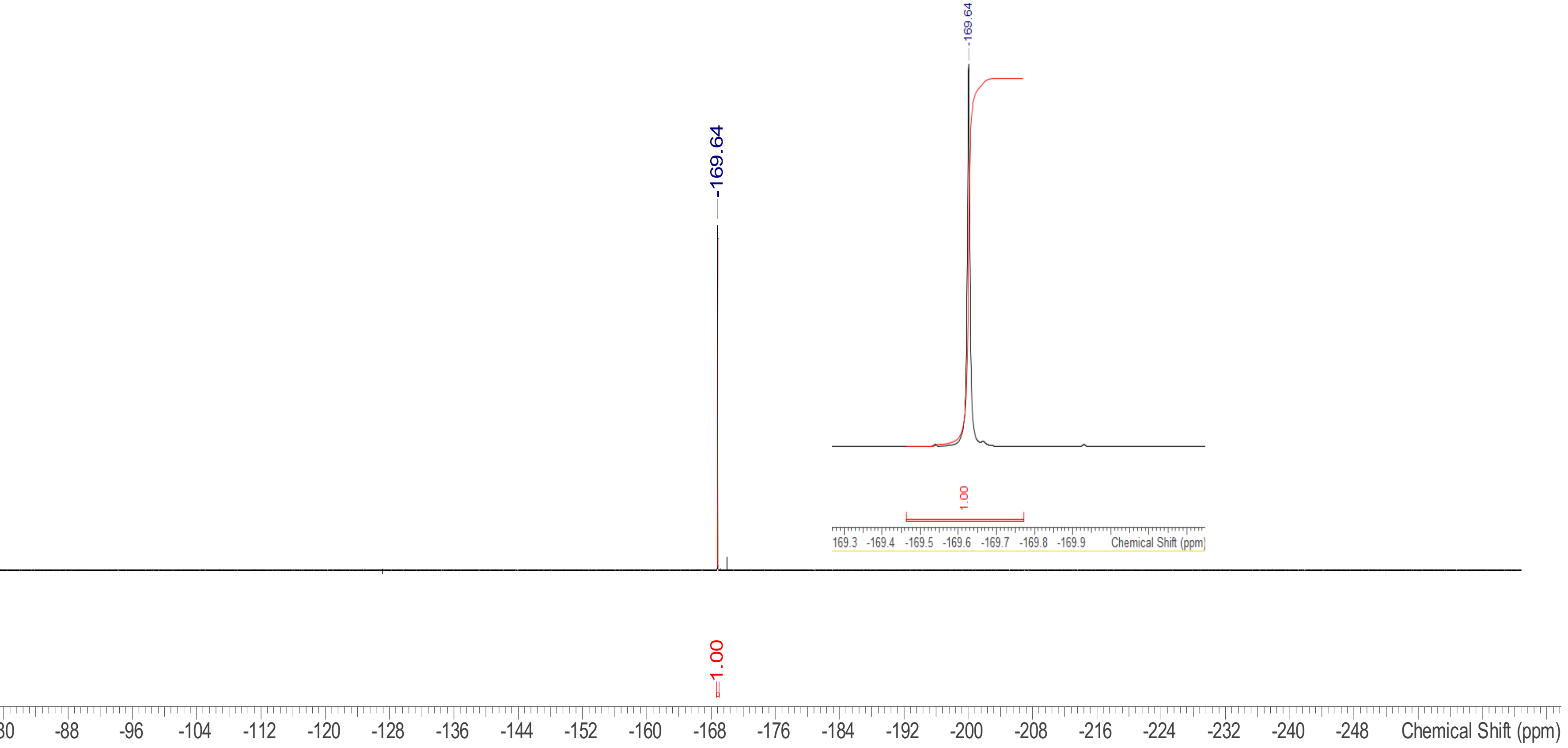

5.6.4 $^{13}C\{^1H\}$ NMR + APT (101 MHz, $CDCl_3$)

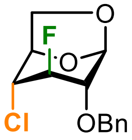

20

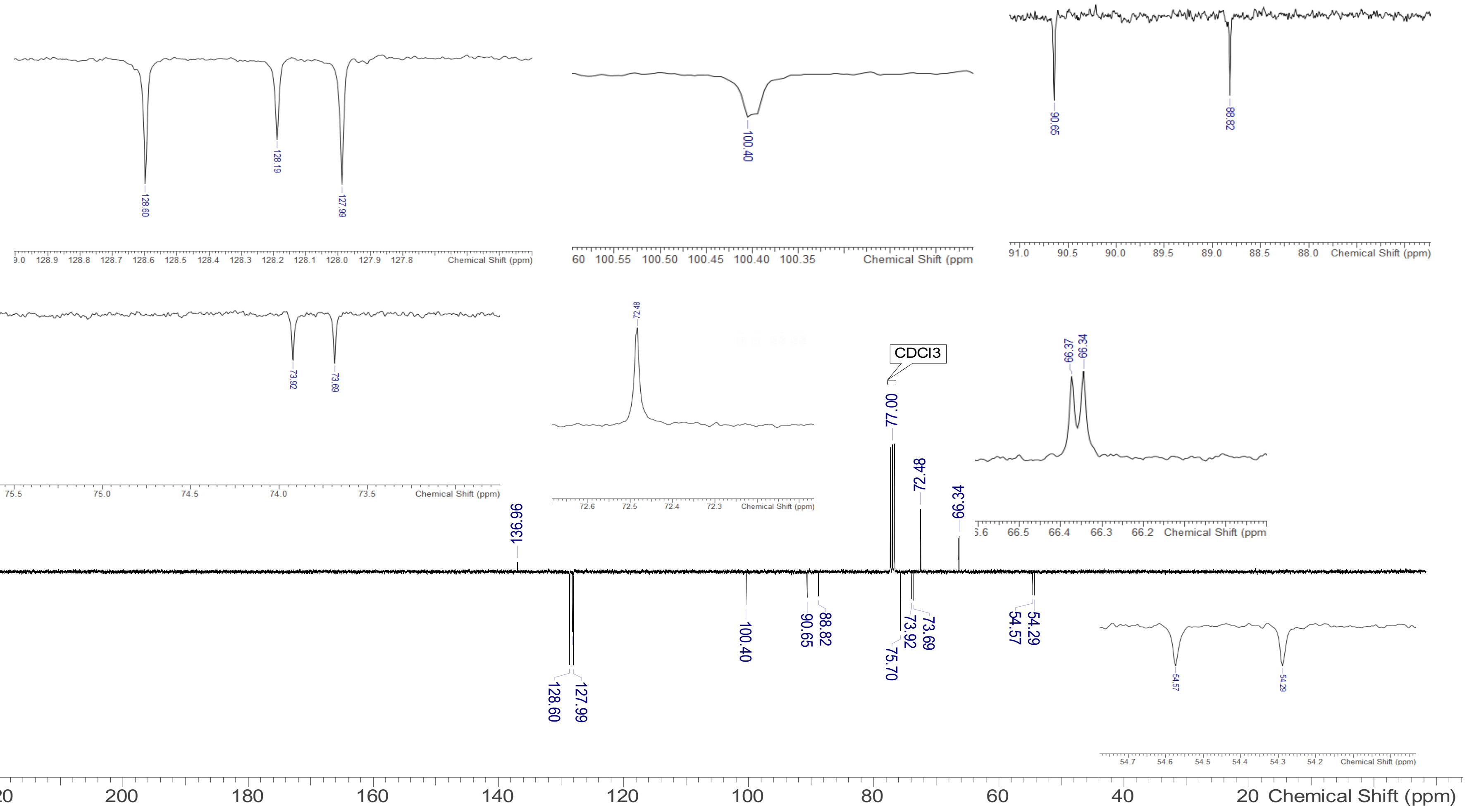

5.7 NMR spectra of 3,4-dideoxy-4-chloro-3-fluoro-D-glucose (11)

5.7.1 ^{1}H NMR (400 MHz, acetone-d_6)

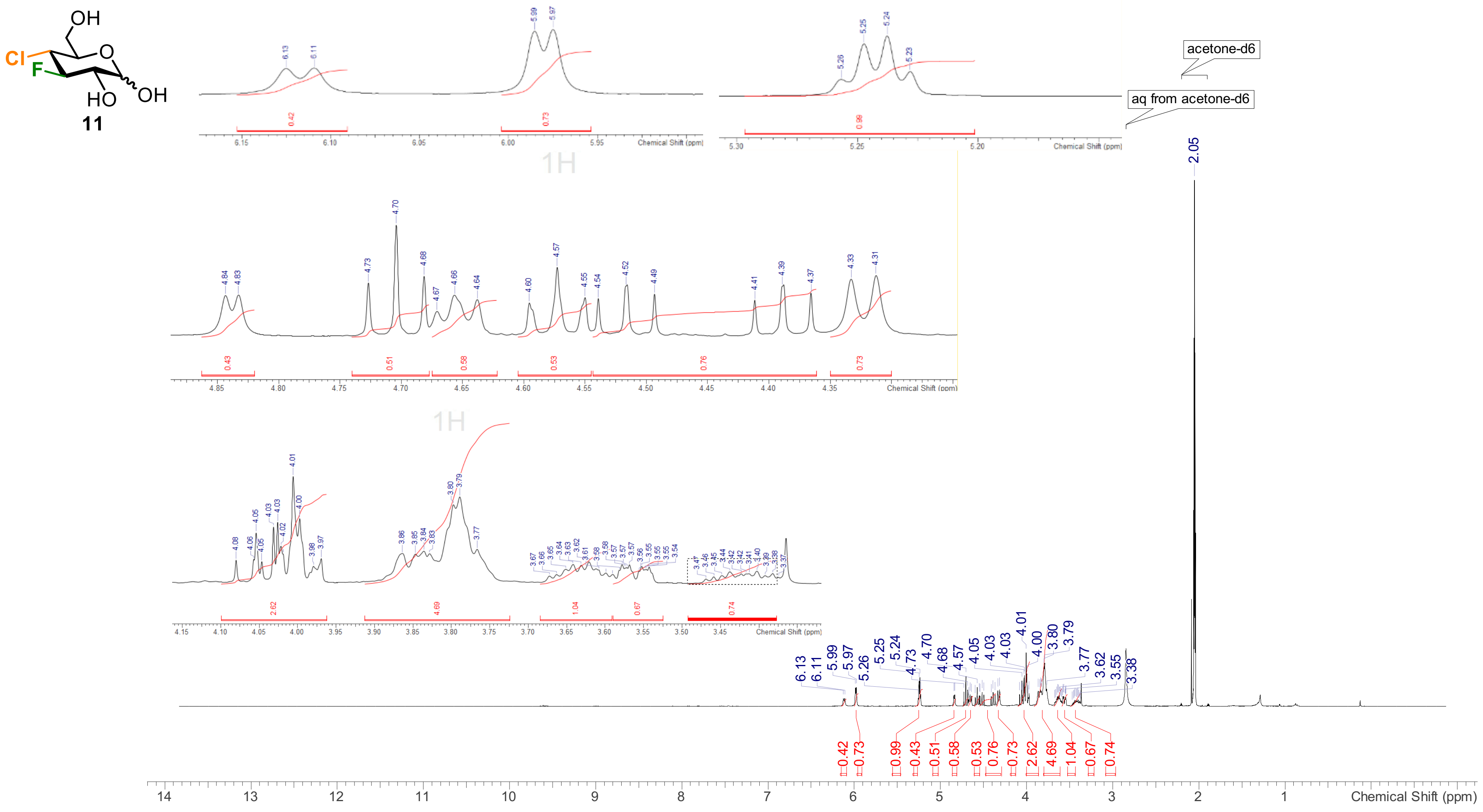

5.7.2 ^{19}F NMR (471 MHz, acetone-d_6)

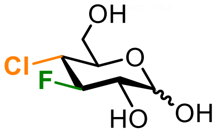

11

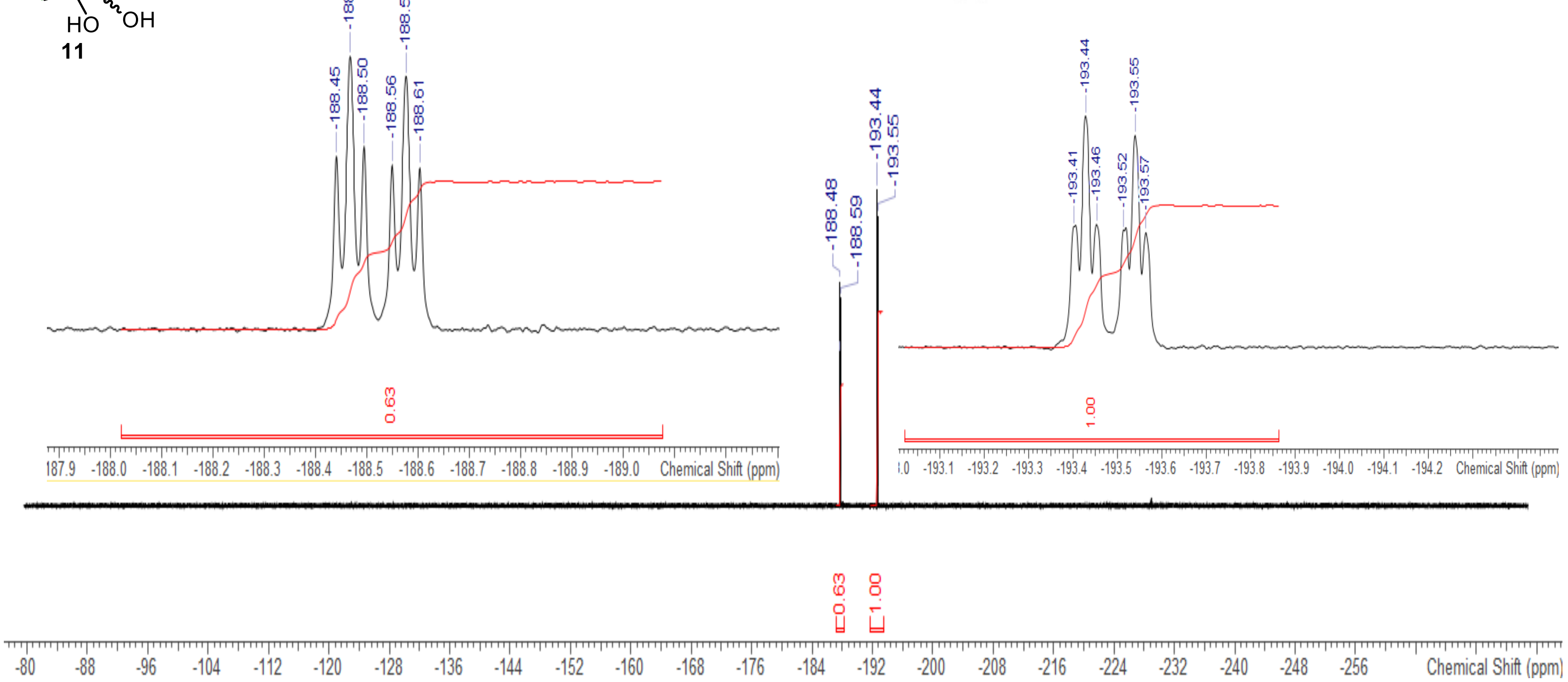

5.7.3 $^{19}F\{^{1}H\}$ NMR (471 MHz, acetone-d_6)

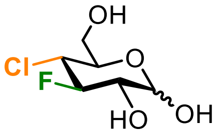

11

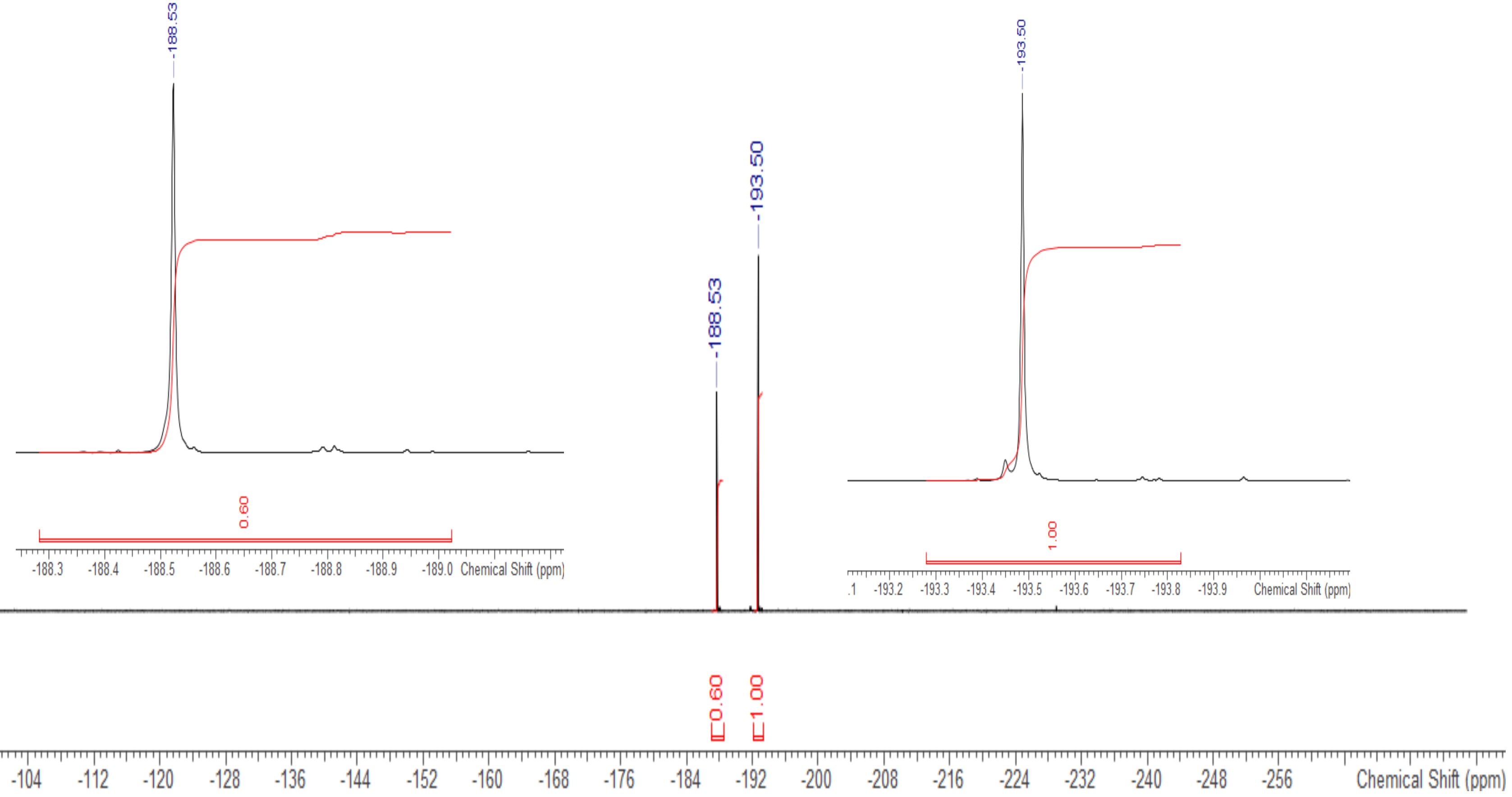

5.7.4 $^{13}C\{^{1}H\}$ NMR + APT (101 MHz, acetone-d_6)

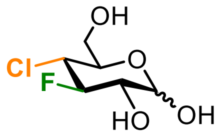

11

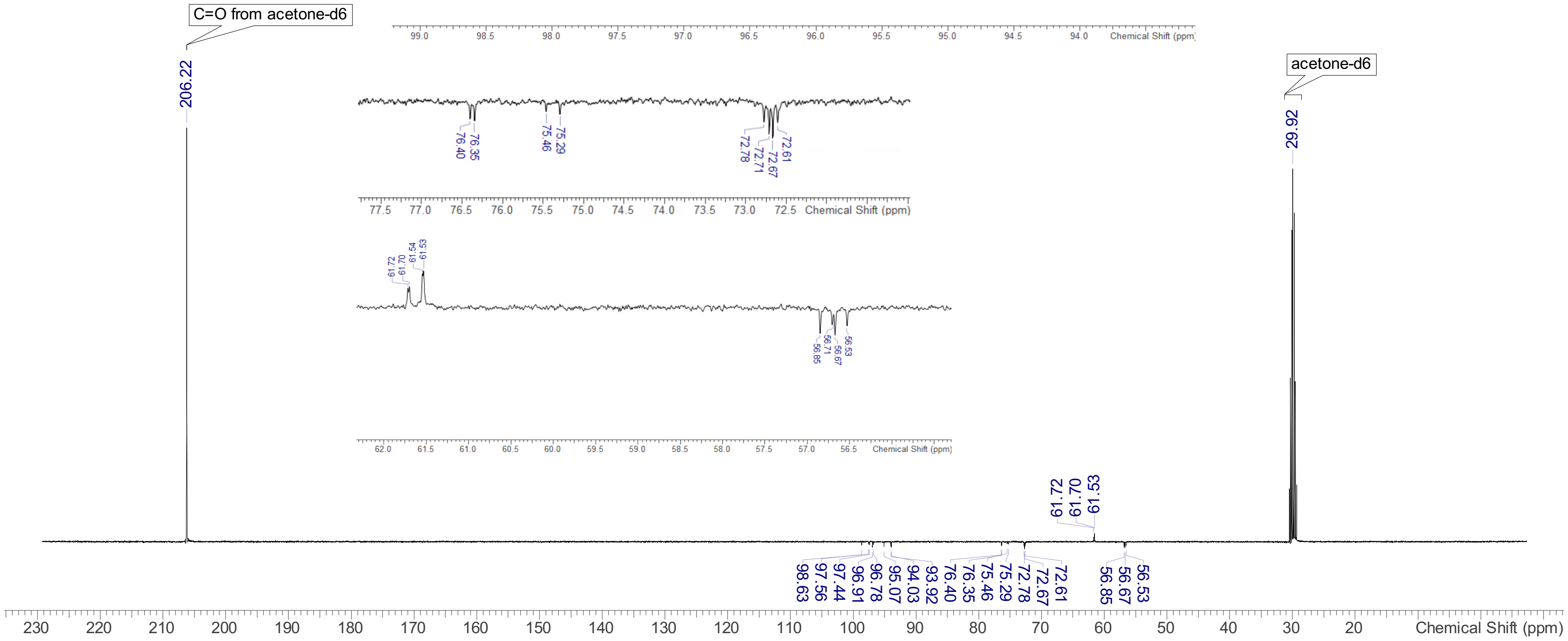

6 References

1. D. E. Wheatley, C. Q. Fontenelle, R. Kuppala, R. Szpera, E. L. Briggs, J. B. Vendeville, N. J. Wells, M. E. Light and B. Linclau, *J. Org. Chem.*, 2021, **86**, 7725-7756.

2. A. S. Sofian and C. Kuan Lee, *J. Carbohyd. Chem.*, 2003, **22**, 185-206.

3. T. B. Grindley, G. J. Reimer, J. Karlovec, R. G. Brown and M. Anderson, *Can. J. Chem.*, 1987, **65**, 1065-1071.

4. M. Oikawa, T. Shintaku, H. Sekljic, K. Fukase and S. Kusumoto, *Bull. Chem. Soc. Jpn.*, 1999, **72**, 1857-1867.

5. G. Procter and D. Genin, *Carbohydr. Res.*, 1990, **202**, 81-92.

6. M. Cerny, I. Buben and J. Pacak, *Collect. Czech. Chem. Commun.*, 1963, **28**, 1569-1578.

7. H. Paulsen, B. Schröder, H. Böttcher and R. Hohlweg, *Chem. Ber.*, 1981, **114**, 322-332.

8. Z. Wang, B. F. Jeffries, H. R. Felstead, N. J. Wells, E. Chiarparin and B. Linclau, *J. Vis. Exp.,* 2019, **143***, e58567*.

9. B. Linclau, Z. Wang, G. Compain, V. Paumelle, C. Q. Fontenelle, N. Wells and A. Weymouth-Wilson, *Angew. Chem. Int. Ed. Engl.*, 2016, **55**, 674-678.
